# Study protocol for a pilot randomized controlled trial on the feasibility and preliminary efficacy of an integrated psychoeducational intervention for transition-age youths in acute psychiatric settings

**DOI:** 10.3389/fpsyt.2026.1768016

**Published:** 2026-02-13

**Authors:** Giulio Longo, Laura Orsolini, Alessandra Premici, Umberto Volpe

**Affiliations:** Unit of Clinical Psychiatry, Department of Clinical Neurosciences/DIMSC, Polytechnic University of Marche, Ancona, Italy

**Keywords:** acute settings, adolescence, adolescents, affective disorders, emotional disorders, psychoeducation, transition psychiatry

## Abstract

**Introduction:**

Psychoeducation is a psychosocial intervention aimed at providing knowledge, cognitive and communication strategies to improve illness awareness and coping skills in patients with psychiatric disorders. Adolescence is a critical phase in psychopathological development. Integrating psychoeducational interventions among youngsters, since hospital clinical practice during mental illness onset, can significantly impact on the global functioning and long-term prognosis. This study aims to assess the feasibility and efficacy of a psychoeducational group intervention to reduce emotional dysregulation and affective symptomatology in a sample of youths hospitalized at the Transitional Psychiatry ward, Marche University Hospital, Ancona, Italy.

**Methods:**

Participants (aged 15-24) will attend weekly thematic modules led by psychiatrists, psychologists and trainees. The control group will receive the treatment as usual. The modules included in the intervention will address the following topics: emotions, anxiety, psychotic experiences, addictions, sleep, therapies, communication and social skills. Participants will be assessed at baseline, at the end of the intervention and at 3-, 6- and 12 months post-randomization.

**Results:**

We expect to observe a significant improvement in the emotional regulation and in the secondary outcomes (mental health literacy, internalized stigma, social functioning, rehospitalization, and coping strategies). Satisfaction questionnaires will also be administered at the end of each session to identify any changes to the intervention, adapting it to patient satisfaction.

**Conclusions:**

The protocol proposes a transdiagnostic and multimodal psychoeducational model aimed at a youth population with high clinical complexity, with potential benefits in terms of secondary prevention, therapeutic continuity and reduction of relapses. Intervention from the acute stages of the illness could ensure greater improvement in the subsequent period, while also establishing a stronger therapeutic alliance.

## 1 Introduction

The term “psychoeducation” etymologically derives by the Greek word “*psykho*-” (or “*psykhē*”, personified as Psykhē, the beloved of Eros, meaning “*mind*”) and the Latin term “*educationem*” (i.e., “training”) (1). Hence, the “psychoeducation” indicates a specific way of training, information and education about the mental, psychological, cognitive and emotional dimensions, with a specific focus on related attitudes, skills and behaviors (1). Indeed, the historical roots of psychoeducation was dated in the early 18th and 19th century writings of Philippe Pinel (1745-1826), Johann H. Pestalozzi (1746-1827), Jean M. Itard (1774-1838), Samuel G. Howe (1801-1876) and Dorothe Dix (1802-1887). These authors described the use of educational methods to provide therapeutic services and assistance to the physically and mentally disabled patients (1). However, despite these very early contributions, the first use of the word psychoeducation appeared only in 1941 within the chapter entitled “The Psychoeducational Clinic” by Brian E. Tomlinson (2). In the context of childhood and adolescence, modern psychoeducational approaches were developed in response to the problems of juvenile delinquency, which increased as a result of the industrial revolution and large-scale immigration (1). At the forefront, between the mid-1940s and the 1960s, Fritz Redl (1902-1988) and his former student David Wineman (1916-1995) developed the “Life Space Interview”, a series of related strategies to help young people reflect on and learn from important interactions and crises. In 2010, in the first edition of “Psychoeducation in Mental Health,” Walsh defined psychoeducation as a series of individual, family, and group interventions. He specified that these interventions focussed on: a) educating participants about life’s challenges; b) helping participants developing social supports and increasing resources to manage these challenges; and, c) developing coping skills. Interventions should also aim to increase emotional support, reduce participants’ sense of stigma, modify participants’ attitudes and beliefs about their own mental disorder, identify and explore feelings about the problem, develop problem-solving skills, and implement crisis intervention skills (3).

Nowadays, psychoeducation is indeed an umbrella term typically used to capture the basic concepts of providing information to patients, either as part of a therapeutic intervention (e.g., psychoeducation about emotions before introducing a mood meter or behavioral activation techniques as part of the treatment for mood disorders) or as the intervention strategy itself (e.g., group psychoeducation) (4). Providing such tools and information to adolescent patients could help them to promptly identify and properly manage the psychopathological trajectory of a mental condition both in prodromal phase and/or at the very early onset stage, while preventing their clinical course and potentially improving the illness prognosis. Psychoeducation is overly utilized in several settings and clinical contexts such as in the prevention and intervention programs, despite it is universally employed in cognitive-behavioural therapy (CBT) in which it provides information with the aim to increase patients’ knowledge about their presenting mental health issues and diagnosis, eliciting their case formulation, and providing a clear rationale for the treatment approach and implementation, and also by facilitating the normalization, alliance-building, demystification process as well as facilitating the treatment engagement and adherence (5–9). Therefore, psychoeducation could potentially reduce negative feelings related to mental health disorders, such as internalized stigma, feelings of shame, and increase the sense of possibility (including hope, optimism, and relief) which can hinder treatment implementation (5, 7).

Despite the growing interest in the application of psychoeducation in adolescents and young people, the available scientific literature is still limited and fragmented. This lack of evidence makes it difficult to define solid and validated guidelines for the use of psychoeducation with adolescents, especially in more complex contexts such as acute psychiatric settings. Within this context, it has been already suggested the need to develop an age-sensitive psychoeducational intervention, able to adequately address specific adolescents’ developmental needs, by promoting a greater emotional awareness, as well as encouraging adherence to treatment pathways (10–14). Therefore, the primary aim of the present study protocol was to assess the feasibility and the efficacy of a pilot psychoeducational group intervention in a sample of adolescents and young adults with a diagnosis of emotional and/or affective disorders hospitalized within our Transition Psychiatry acute psychiatric ward, in terms of reduction of dysregulated emotional and affective symptomatology in the post-intervention and in the follow-up period. The feasibility will be assessed considering recruitment and retention rates and acceptability of the intervention, while the efficacy will be evaluated through the evaluation of emotional dysregulation pre- and post-intervention. Secondary outcomes included the evaluation of the efficacy of the intervention on the following dimensions: a) improvement of mental health literacy; b) reduction of internalized stigma; c) improvement of psychosocial functioning; d) reduction of relapses and hospitalizations during the 12-months follow-up period; e) improvement of coping strategies and expressed emotions.

## 2 Methods

A pilot single-centre randomized controlled intervention study was developed to be carried out at the Transition Psychiatry ward (specifically addressed to adolescents and young adults aged 15-24), Unit of Clinical Psychiatry, Department of Neurosciences/DIMSC, University Hospital of Marche Region within the Polytechnic University of Marche, Ancona, Italy. The randomization procedure will be performed using IBM Statistical Package for Social Sciences software, version 26 (SPSS, Chicago, Illinois, USA). For each participant, a random number will be generated using the RV.UNIFORM(0,1) function, which produces values distributed uniformly between 0 and 1. The cases will be then sorted according to the random number obtained and will be assigned to the two experimental groups in a 1:1 ratio. The allocation sequence will be generated by an independent researcher not involved in recruitment or intervention delivery, in order to ensure concealed allocation and minimize possible bias. The entire procedure will be documented to ensure replicability and methodological transparency.

Patients referred to the inpatient Transition Psychiatry unit will be invited to participate in the study if they meet the following inclusion criteria: a) diagnosis of an emotional and/or affective Disorder (i.e., Major Depressive Disorder, Bipolar Disorder, Cyclothymia, Persistent Depressive Disorder, Seasonal Affective Disorder, Disruptive Mood Dysregulation Disorder, Other Specified Depressive Disorder, Unspecified Depressive Disorder, Other Specified Bipolar and Related Disorder, Unspecified Bipolar and Related Disorder, Unspecified Mood Disorder, Oppositional Defiant Disorder, Intermittent Explosive Disorder, Conduct Disorder, Other Specified Disruptive, Impulse-Control, and Conduct Disorder, Unspecified Disruptive, Impulse-Control, and Conduct Disorder) according to the Diagnostic and Statistical Manual of Mental Disorders, Fifth Edition text revised (DSM-5-TR) (15); b) aged 15–24 years old; c) admitted to the acute unit of Transition Psychiatry and expressed the willingness to participate to all the psychoeducational program also in the post-discharge sessions; d) agreement to the study participation during the 12-months follow-up period; e) ability to provide written informed consent (their parents in case they are less than 18 years old). This broad inclusion strategy was adopted to reflect the heterogeneous and often overlapping nature of early psychopathological manifestations during the transition age. In particular, the use of DSM-5-TR diagnoses should consider a dimensional and transdiagnostic framework, because symptom presentations in youths frequently evolve over time and may not yet conform to stable categorical boundaries. Anyways, if a patient is admitted to our department without a prior diagnosis, study inclusion is based on the preliminary diagnosis formulated at admission to our ward. Exclusion criteria will include: a) presence of an intellectual disability and/or mental retardation and/or difficulty in understanding Italian language and/or learning and/or moderate-to-severe cognitive deficits (absolute contraindication); b) patients with current uncontrollable psychomotor agitation and/or current drug and/or alcohol intoxication which may interfere with attention and/or concentration during the psychoeducational sessions (relative contraindication); c) the presence of an acute psychiatric symptomatology which may impact the level of consciousness and/or induce a temporary alteration of cognitive ability (relative contraindication). All included participants will continue to receive the treatments usually provided (e.g., regular psychiatric visits, pharmacological treatments, and psychotherapy). The study will be carried out in accordance with globally accepted standards of good practice and in agreement with the Declaration of Helsinki and with local regulations. An informed consent form will be signed by their parents after full description of study protocol. The study investigators will ensure that all mental health professionals involved in the study are qualified and informed about the protocol, interventions, and trial-related duties. An ethical approval request has been submitted (ID study: 4696) to our local Institutional Ethical Committee of Marche Region and will be obtained before starting with the recruitment.

## 3 Interventions

### 3.1 Experimental intervention

The experimental intervention will consist of techniques derived from the classical Falloon psychoeducational intervention and from the adaptations by CBT approach, both adapted for the treatment of adolescents and young adults affected by emotional and/or affective disorders in an acute psychiatric setting. The experimental intervention will be administered in a daily group context of 1-1.5 hours each within the acute psychiatric settings (during the hospitalization phase). The adoption of a daily psychoeducational intervention during hospitalization was motivated by the need to deliver a comprehensive intervention within the limited timeframe preceding discharge, considering the low length of stay in our ward. If the patient is discharged before the end of the procedure, patients can conclude the procedure in person or via teleconference. The experimental intervention will consist of the following phases: 1) engagement of the patient; 2) individual assessment; 3) informative sessions provided within the group context; 4) communication skills sessions provided within the group context; 5) problem-solving skills sessions provided within the group context; 6) social skills sessions provided within the group context. The informative sessions will deal with the following thematic modulus: 1) Primary and secondary emotions; 2) Anxiety and Sadness; 3) Unusual experiences; 4) Drugs and addictions; 5) Sleep and Biological Rhythms; 6) Medications (**Table 1**).

**Table 1:** Characteristics of each module of the experimental intervention.

Module	Description
Primary and secondary emotions 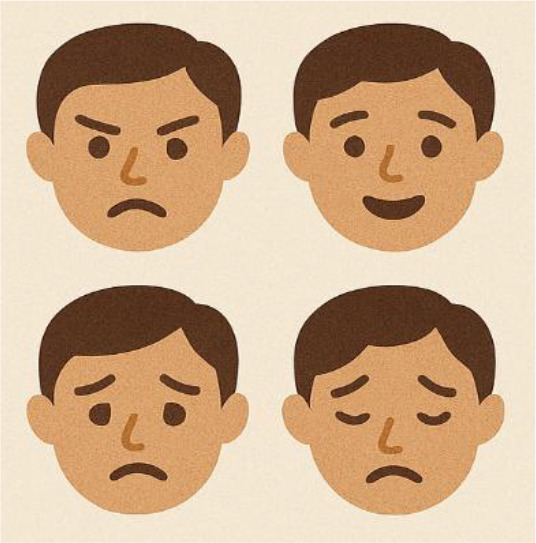	It provides information on Ekman’s five primary emotions, contextualizing them through facial expressions and teaching their recognition, including emotions’ body experiences. Patients also will learn the ABC model (16, 17) for managing unpleasant emotions and related thoughts.
Anxiety and Sadness 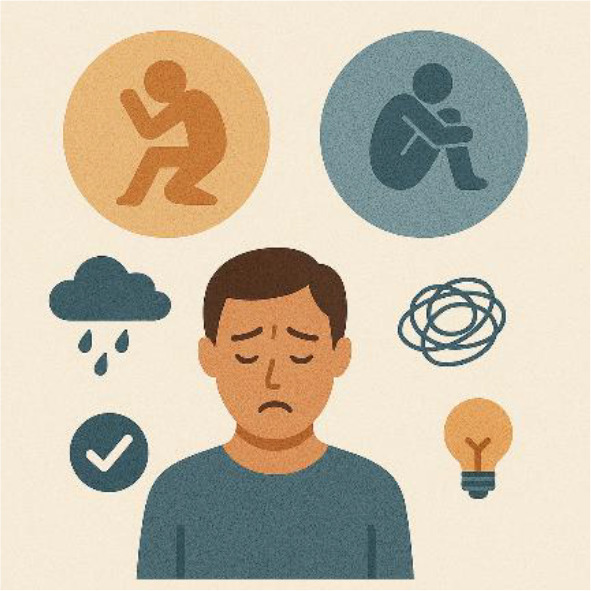	It focuses on the differences between normal and pathological forms of these emotions, identifying triggering situations and body experience of anxiety and sadness. It will also teach behaviors that can reduce vulnerability to anxiety, along with strategies to manage both emotions effectively.
Unusual experiences 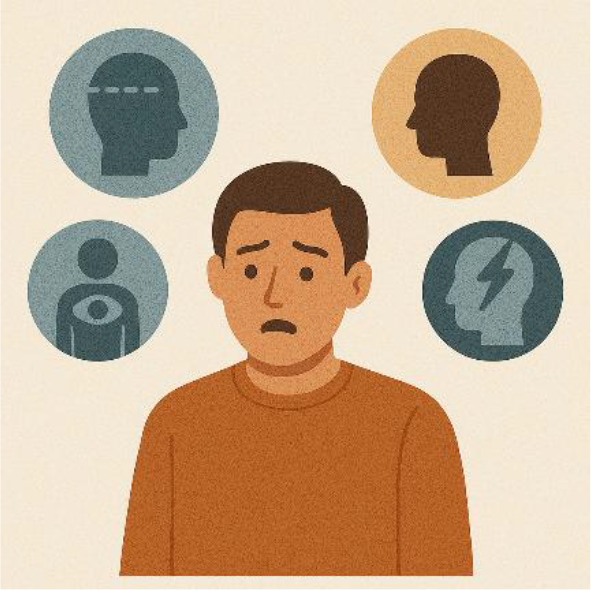	It provides information regarding the manifestation of different “unusual experiences” that patients may develop like dissociation, derealization, depersonalization or hallucinations.
Drugs and addictions 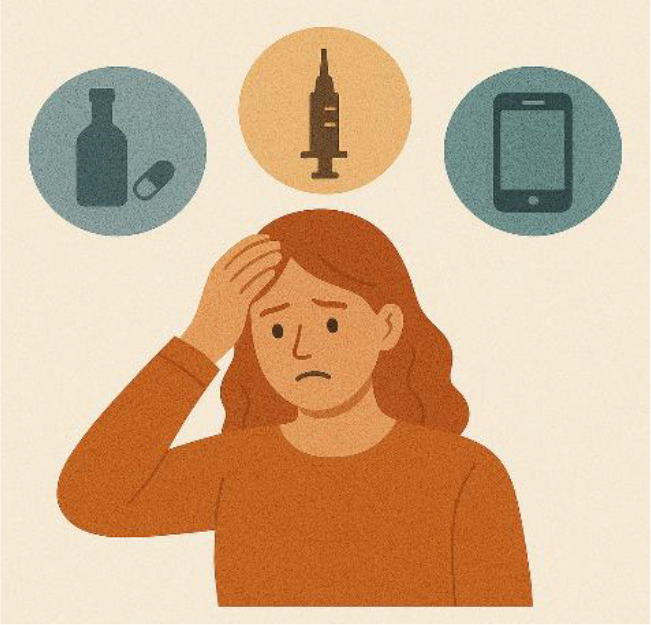	It aims to raise awareness about substances and their associated risks, offering alternative coping strategies to substance use and addressing possible behavioral addictions, particularly digital ones.
Sleep and Biological Rhythms 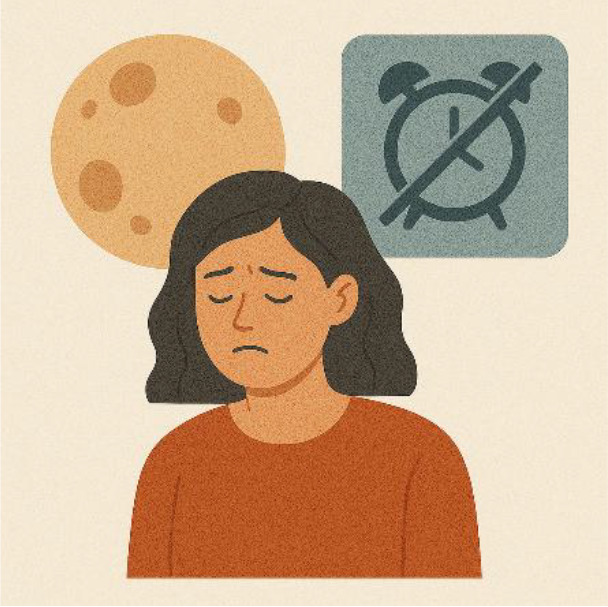	It provides information about sleep mechanisms, proper sleep hygiene, factors influencing sleep quality (both positively and negatively), and strategies to improve rest.
Medications 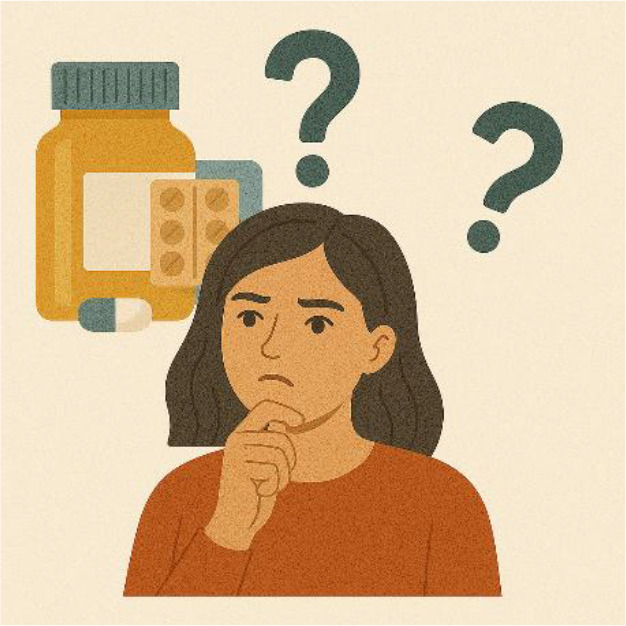	Available pharmacological and non-pharmacological treatments for mental disorders, indications, side effects and strategies to cope with them, treatment duration, risk factors for relapse, and effects related to abrupt discontinuation of the therapy.

All images were generated using AI.

The sessions will take place each day for a total of 12 sessions for a period of around 2 weeks. Between sessions, participants will be invited to fill out dedicated practical exercises (“homeworks”). Each session will be organized into three phases: a) the first phase will be dedicated to clarifications and questions regarding the topics previously discussed; b) the second phase will focus on the main topics of the session; and, c) in the final phase, the key points addressed during the session, will be summarized with the assignment of target homeworks to all group members. A dedicated training material will be provided to all staff members in order to guarantee the fidelity of administration of all sessions of the experimental intervention, after providing a formal training by an experienced trained clinician (**Supplementary Material 1**). At the end of each session, all participants will complete a satisfaction questionnaire in order to improve and adapt the intervention based on participants’ feedback. **Supplementary Material 2** shows the satisfaction questionnaire that has been specifically developed. At the end of each session, participants will receive informative leaflets and cards summarizing the key-points addressed during the session (**Supplementary Material 3**).

### 3.2 Measurements, assessment time-points and tools

**Figure 1:**
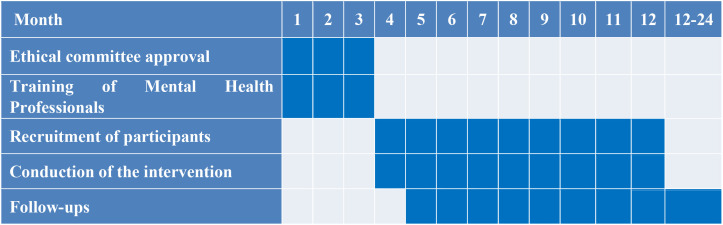
Timeline of the study.

Before the administration of the study, during the first three months of the study the following tasks have been planned: a) the formal achievement of EC approval; b) the training of mental health professionals in the experimental interventions and in the use of assessment tools; c) organizing the implementation of the interventions. The recruitment of participants will take place between month 4 and month 12. The participants will be randomly assigned by the principal investigator (P.I.) only after the receipt of written informed consent by their parents (if aged less than 18 years old). Assessment will be performed between month 4 and month 24. **Figure 1** illustrates the timeline of the study.

At the baseline will be collected socio-demographic (e.g., sex, age, educational level, occupational and living condition) and clinical characteristics (e.g., age of onset, illness duration, number of affective and/or emotional episodes and previous hospitalizations, age at first hospitalization, comorbid physical and/or mental disorder, family psychiatry history, suicidality, drug and/or alcohol use current and/or previous, current and/or previous pharmacological and/or psychosocial and/or psychotherapy interventions) of all eligible participants through validated assessment tools. Researchers who administer assessment tools, will be blinded to patient allocation. All patients will be assessed at the following time points: a) at T0 (baseline), at least 1 day before the start of the intervention; b) at T1 (post-intervention); c) at T2 (follow-up at 1 months after completion of intervention); d) at T3 (follow-up at 3 months after completion of intervention); and, e) at T4 (follow-up at 6 months after completion of intervention); f) at T5 (follow-up at 12 months after completion of intervention). During the assessment, whereas the participant feels distressed or exhausted, the assessment will be postponed in another moment in a range of 2–3 days.

At baseline, participants will be administered the following assessment tools: Difficulties in Emotion Regulation Scale (DERS) (18), Brief Psychiatric Rating Scale (BPRS) (19), Clinical Global Impression Scale - Severity (CGI-S) (20), Coping Orientation to Problems Experienced-New Italian Version (COPE-NVI) (21), Global Assessment of Functioning (GAF) (22), Internalized Stigma of Mental Illness Inventory (ISMI) (23), Mental Health Knowledge Schedule (MAKS) (24) and the Montreal Cognitive Assessment (MoCA) (25) (**Tables 2**, **3**).

**Table 2:** Timeline of the assessment administration.

Assessment	T0	T1	T2	T3	T4	T5
DERS	X	X	X	X	X	X
BPRS	X	X	X	X	X	X
CGI-S	X	X	X	X	X	X
CGI-I		X	X	X	X	X
COPE-NVI	X	X	X	X	X	X
GAF	X	X	X	X	X	X
ISMI	X	X	X	X	X	X
MASK	X	X	X	X	X	X
MOCA	X					

T0, baseline; T1, end of the intervention; T2, 1 month after the end of the intervention; T3, 3 month after the end of the intervention; T4, 6 month after the end of the intervention; T5, 12 month after the end of the intervention; DERS, Difficulties in Emotion Regulation Scale; BPRS, Brief Psychiatric Rating Scale; CGI-S, Clinical Global Impression Scale - Severity; CGI-I, Clinical Global Impression Scale - Improvement; COPE-NVI, Coping Orientation to Problems Experienced-New Italian Version, GAF, Global Assessment of Functioning; ISMI, Internalized Stigma of Mental Illness Inventory; MAKS, Mental Health Knowledge Schedule; MOCA, Montreal Cognitive Assessment (MoCA).

**Table 3:** Characteristics of the assessment used in the study.

Assessment	Description
DERS (18)	The DERS is a widely used scale for assessing difficulties in emotion regulation. It consists of 36 self-report items on a 5-point Likert scale, with responses ranging from 1 to 5, from “almost never” to “almost always”. Total scores range from 36 to 180, with higher scores indicating greater problems with emotion regulation. The Italian version identifies the following subscales: non-acceptance of emotional responses, difficulty engaging in distracting behaviours, difficulty controlling impulses, lack of emotional awareness, limited access to emotion regulation strategies, lack of emotional clarity. This scale will be used to assess the impact of the intervention on emotion regulation skills.
BPRS (19)	The BPRS is the most widely used inventory of psychiatric symptoms for assessing disease severity, particularly for evaluating outcomes in psychopharmacological and psychosocial clinical trials. The test consists of a semi-structured interview with 24 items, with a severity score of 7 points, completed by the interviewer. Scores below 30 indicate mild symptoms, while scores between 31 and 41 indicate moderate symptoms and scores above 42 indicate severe symptoms. This scale will be used to assess whether the intervention has an effect in reducing the severity of symptoms.
CGI-S and CGI-I (20)	The CGI has two components: the CGI-S, which rates illness severity, and the CGI-I, which rates change from the initiation (baseline) of treatment. The CGI-S is a scale that assesses the severity of illness by asking the evaluator a question: ‘Based on your experience with this type of patient, how ill, from a psychological point of view, is the patient at this time?’ The degree of illness consists of seven levels: 1 = normal, not ill at all; 2 = only marginally ill; 3 = mildly ill; 4 = moderately ill; 5 = significantly ill; 6 = severely ill; 7 = among the most severely ill patients. This assessment is based on symptoms, behaviour and functioning observed and reported over the last seven days. It is clear that symptoms and behaviour can fluctuate over the course of a week; the score should reflect the average level of severity over the seven days. The CGI-I is similarly simple in its format. Each time the patient is visited after a treatment initialization, the clinician compares the patient’s overall clinical condition to the one-week period just prior to the initiation of the intervention. This scale will be used to assess whether the intervention has an effect in reducing disease severity.
COPE-NVI (21)	The COPE-NVI is a 28-item self-report questionnaire designed to measure effective and ineffective coping strategies in response to a stressful life event (21). In this questionnaire, patients must indicate the frequency with which they use a series of coping strategies during stressful/challenging events, using a 4-point Likert scale (ranging from “I don’t usually do this = 1” to “I almost always do this = 4”). It consists of five basic scales: a) social support (referring to seeking comfort); b) avoidance strategies (denial, substance use, detachment from reality); c) positive attitude (acceptance, containment and positive reinterpretation); d) problem-focused orientation (active and strategic attitude); e) transcendent orientation (religion and lack of humour). This scale will be used to assess whether the patient develops more adaptive coping strategies as a result of the psychoeducational programme.
GAF (22)	The GAF index is a scale introduced in DSM-IV, used by mental health specialists to subjectively assess an individual’s social, occupational and psychological functioning in response to different life problems. Scores range from 100 (extremely high functioning) to 1 (severely impaired), divided into 10 levels. This scale will be used to assess the impact of psychoeducational intervention on patients’ functioning.
ISMI (23)	The ISMI scale is a 29-item questionnaire measuring self-stigma among persons with psychiatric disorders, developed in collaboration with people with mental disorders. Each item is scored from 1 (disagree) to 4 (strongly agree). This scale will be used to evaluate the fluctuations of self-stigma after the psychoeducation intervention.
MASK (24)	The Mental Health Knowledge Schedule (MAKS) is a standardised tool developed to measure the level of mental health knowledge in the general population. The scale is composed of 12 items, divided into a section on general knowledge (help seeking, recognition, support, employment, treatment, and recovery) and one dedicated to knowledge of mental illness conditions, with responses on a 5-point Likert scale. The MAKS is widely used in prevention programmes and anti-stigma campaigns as a summary indicator of mental health literacy, given its brevity and ease of administration. It will be used to assess mental health literacy after the intervention.
MOCA (25)	The Montreal Cognitive Assessment (MoCA) is a brief neuropsychological screening tool designed for the early identification of cognitive impairment and initial cognitive deficits in major neurodegenerative and cerebrovascular diseases. The test assesses multiple cognitive domains (executive functions, attention, memory, language, visuospatial skills and orientation) through a heterogeneous battery of tasks. The maximum score is 30, with a commonly adopted cut-off of 26/30, and with a correction of +1 point for subjects with ≤ 12 years of schooling. This test will be used to assess the initial cognitive level of participants in the study, trying to identify any subthreshold cognitive impairment. It will not be used as an outcome measure.

DERS, Difficulties in Emotion Regulation Scale; BPRS, Brief Psychiatric Rating Scale; CGI-S, Clinical Global Impression Scale - Severity; CGI-I, Clinical Global Impression Scale - Improvement; COPE-NVI, Coping Orientation to Problems Experienced-New Italian Version, GAF, Global Assessment of Functioning; ISMI, Internalized Stigma of Mental Illness Inventory; MAKS, Mental Health Knowledge Schedule; MOCA, Montreal Cognitive Assessment (MoCA).

During the follow-up phases, participants will be administered the following assessment tools: DERS, BPRS, Clinical Global Impression Scale - Improvement (CGI-I) (20), CGI-S, COPE-NVI, GAF, ISMI, MAKS (**Tables 2**, **3**).

### 3.3 Control intervention

The control group will receive an educational 6-session intervention, daily administered, on the following topics: a) healthy lifestyle (diet and nutrition); b) stress management; c) regulation of circadian rhythms; d) management of medication side effects (**Table 4**). The control group intervention will be administered with interacting sessions lasting 60–90 minutes provided within the group context. At the end of each session, participants will receive informative leaflets and cards summarizing the key-points addressed during the session. A dedicated training material will be provided to all staff members in order to guarantee the fidelity of administration of all sessions of the control intervention.

**Table 4:** Characteristics of each module of the control intervention.

Module	Description
Healthy lifestyle (diet and nutrition) 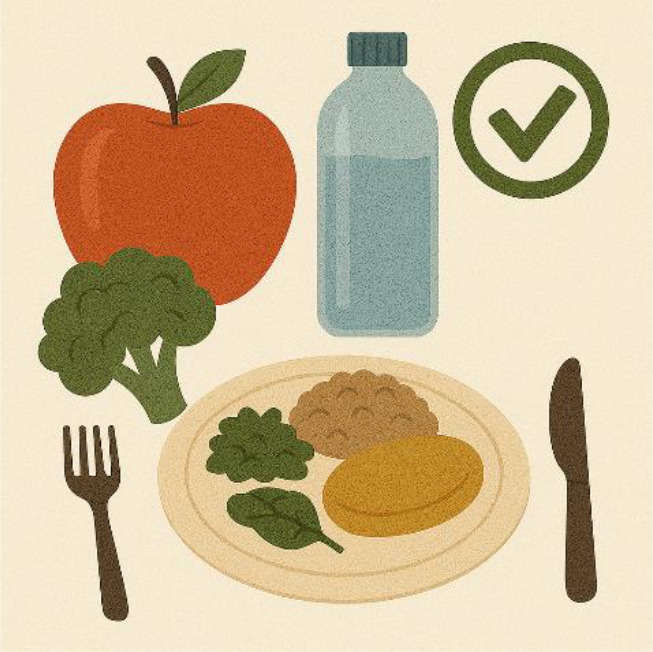	The module emphasises the importance of a varied and balanced diet for maintaining energy levels, a healthy weight and general well-being. It includes practical advice on calorie distribution throughout the day, regular consumption of fruit and vegetables, choosing whole grains and staying hydrated.
Healthy lifestyle (physical activity) 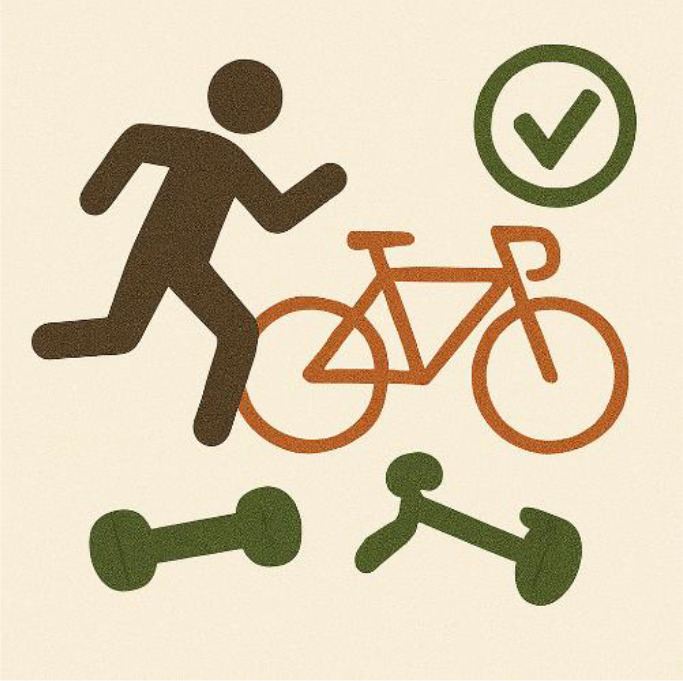	The module promotes daily exercise as a means of improving sleep, mood, weight and quality of life. Simple ways to increase the amount of time spent being active and reduce sedentary behaviour are suggested.
Stress management 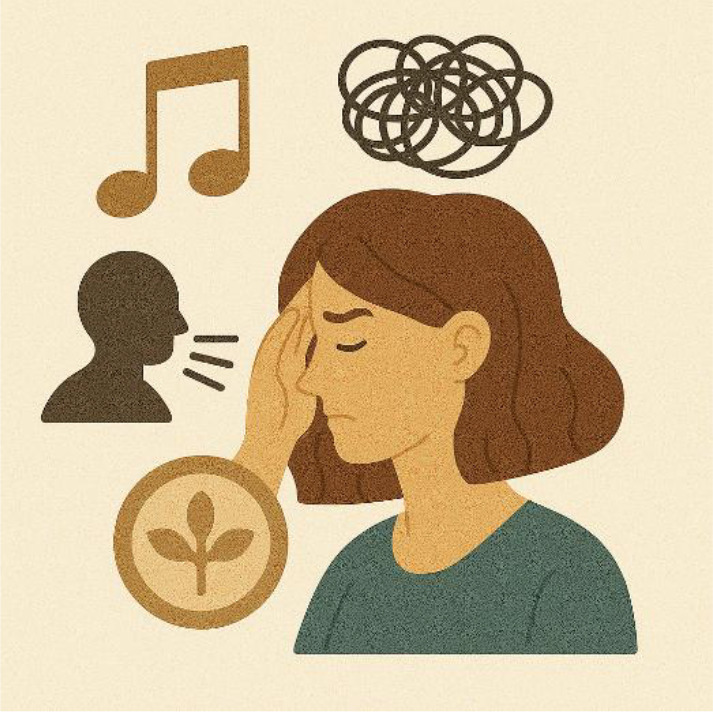	The module helps to recognise physical, emotional and cognitive manifestations of stress and suggests immediate management exercises (breathing, muscle relaxation, music). The aim is to reduce the impact of stress in everyday life and prevent relapses.
Regulation of circadian rhythms 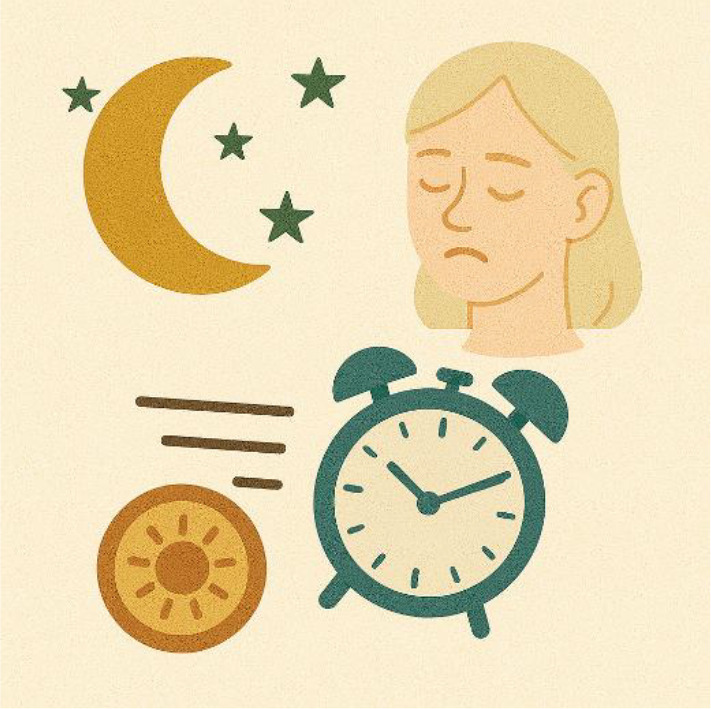	The module explores the role of sleep in maintaining mental and physical health, linking insomnia and irregular sleep to irritability, reduced concentration and physical problems. It provides environmental, dietary and behavioural guidelines to promote a healthy sleep-wake cycle.
Management of medication side effects 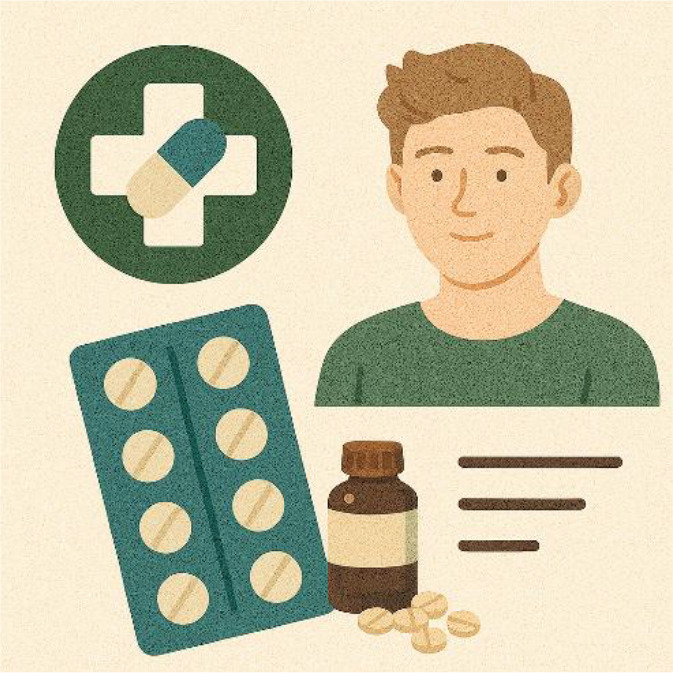	The module clarifies that mental disorders benefit most from a combination of pharmacological treatment and psychotherapy. It reduces prejudices about drugs, explains the main categories used and emphasises the importance of therapeutic adherence to prevent relapses.

All images were generated using AI.

## 4 Training of mental health professionals

All mental health professionals involved in the study protocol will be adequately trained in an *ad hoc* 4-day training course about the use of the experimental and control interventions. During the training, learning material explaining how to conduct the experimental and control interventions will be provided to all mental health professionals. In addition, specific learning role-play sessions will be organized to test both interventions in all trainers, under the supervision of a specialized and experienced trained clinician. All mental health professionals who will be involved in the assessment phase of the study protocol will be adequately trained and instructed about all assessment tools and instruments, before the start of the intervention.

## 5 Statistical analysis

Sample size has been calculated using the Statistical Software G*Power version 3.1. (Franz, Universitat Kiel, Germany). Based on the primary objective of the study, in order to detect any difference in the mean scores of the subscales of the DERS between the control and the experimental groups, by keeping a statistical power of 0.95 to identify an effect size of 0.8 (α=0.05, two-tailed) and taking into consideration all variables to be entered in the t-test. A total sample size of 84 was established to be reached for the present study (42 participants for each group). Considering the longitudinal follow-up and the clinical instability of the study population, attrition and missing data are expected. Analyses will be conducted on available data, with attrition rates systematically reported. Given the pilot nature of the trial, results will be interpreted with a focus on feasibility and preliminary efficacy. The IBM Statistical Package for Social Sciences software, version 26 (SPSS, Chicago, Illinois, USA), will be used for the data analysis. Descriptive analyses will be performed to describe the socio-demographic characteristics of the sample and to assess the normality of the data. Categorical variables will be summarized as frequency (n) and percentage (%), while continuous variables will be summarized as mean and standard deviation (SD). Categorical variables will also be compared using the χ^2^-test and *post-hoc* tests. To summarize the data and test the differences based on categorical analysis, parametric tests (t-test or ANOVA) or non-parametric tests (Kruskal-Wallis test or Mann-Whitney U test) will be used, depending on the normality of the data. To compare longitudinal data, the paired t-test or Wilcoxon test will be used, again based on normality. Finally, correlation and regression analyses (univariate/multivariate, linear/logistic) will be conducted, including socio-demographic and clinical variables and the total scores and subscales of the various scales, to explore any relationships between the different factors. The statistical significance cut-off will be set at p <0.05.

## 6 Discussion

This protocol describes a randomised pilot clinical trial aimed at assessing the feasibility and efficacy of an integrated psychoeducational intervention for adolescents and young adults with emotional and affective disorders recruited at an acute Transition Psychiatry hospital setting. The implementation of structured and standardised interventions in acute psychiatric settings may offer substantial advantages in terms of treatment adherence, illness awareness, mental health literacy and, more broadly, clinical course and prognosis. Rather than acting on primary prevention, which is no longer feasible once hospitalisation has occurred, such interventions could contribute to improve patients’ capacity to recognise early signs of relapse, increase insight and support continuity of care after discharge. Moreover, the possibility of engaging individuals directly during hospital admission allows for timely monitoring of psychopathological trajectories that are often characterised by clinical instability, high relapse risk, recurrent service utilisation and significant psychosocial impairment. In this perspective, psychoeducation represents a highly promising approach, offering knowledge, cognitive strategies, and relational tools essential for promoting awareness, empowerment, and greater adherence to treatment (9, 26–29). International literature has highlighted how psychoeducational interventions, both individual and group-based, can improve mental health literacy, reduce internalised stigma, increase emotional skills, and implement more adaptive coping strategies (5, 6, 8, 9, 30–32). These dimensions represent key clinical targets, since poor illness awareness, maladaptive emotional regulation, and high internalised stigma have been consistently associated with impaired treatment adherence, increased relapse risk, disengagement from services, and poorer functional recovery. Strengthening these domains during the acute phase is therefore not merely an educational objective but a prognostic intervention.

However, there is still a significant lack of studies focusing on adolescents and young adults, particularly in acute settings. This is probably due to the fact that many mental health systems are clearly divided into pediatric services (childhood/adolescence) and adult services, without an intermediate “adolescence-to-adulthood transition” area that takes into account the clinical and developmental characteristics typical of young people (33–36). In addition, young people often hesitate to seek help for mental health problems due to stigma (social and self-stigmatization), shame, or fear of judgment (33–36). At the same time, services are often inadequate or not designed for “transition and flexibility” as a youth-centered model would require, resources are limited with difficulties in funding and maintaining dedicated units, and there is a lack of continuity between hospital settings and community services (33–36). Our protocol may help to fill this gap by proposing an integrated and systematic model that is not limited to the transmission of information but includes communication, social and problem-solving skills, which are particularly relevant in a vulnerable developmental phase such as adolescence. One of the main strengths of this study is the multimodal and transdiagnostic approach adopted. The use of thematic modules on emotions, anxiety, psychotic experiences, addictions, sleep and therapies allows us to intervene on clinical dimensions commonly manifested in youth emotional and affective disorders, supporting the growing trend towards a more dimensional approach towards youth psychopathology (37–40). Furthermore, the continuation of the intervention (in-person or via teleconference) even after discharge promotes continuity of care, a crucial element in preventing relapses and consolidating the therapeutic alliance. The study design, which includes a comparison with a control group receiving the usual treatment and a specific educational activity, will allow us to assess whether the experimental approach has added value compared to basic health information alone. This approach will not only verify the efficacy of the intervention but also identify which specific psychopathological dimensions are most influenced by psychoeducation.

This study has some potential limitations. First, it is a pilot trial with a relatively small sample recruited from a single centre, factors that could potentially limit the generalisability of the results. However, if the results confirm the expected efficacy, the protocol may serve as a model that can be replicated in other hospital and community settings, helping in increasing evidence at its support, with significant implications for the secondary and tertiary prevention of adolescent psychiatric disorders. At the same time, the diagnostic heterogeneity of participants could lead to an increased variability in outcomes and make it difficult to interpret results. However, this reflects the clinical reality of patients admitted to our ward in this age group, which cannot be excluded. Accordingly, the primary aims of this study are to evaluate feasibility and preliminary efficacy, rather than disorder-specific treatment effects. Furthermore, the target population, consisting of adolescents and young adults in the acute phase, may experience levels of emotional instability that could affect their consistent participation in sessions and completion of follow-up assessments. At the same time, systematic feedback from participants, collected through satisfaction questionnaires, will allow the programme to be further refined to better meet the needs of young users, improving the intervention and promoting patients engagement. A further limitation concerns the possible confounding effect of concomitant pharmacological and psychotherapeutic treatments, which are difficult to control in a real clinical setting. Although we recognize that in a natural clinical setting, complete control of concomitant treatments cannot be guaranteed, measures will be taken (detailed recording of treatment regimens, statistical control, and subgroup analysis) to minimize variability caused by treatment changes and to strengthen the reliability of causal inferences in this protocol.

However, such limitations are common in adolescent psychiatry research and do not preclude the usefulness of a pilot study for exploratory purposes. The early integration of psychoeducation, social skills and problem-solving strategies may help to modify the developmental trajectory of emotional disorders and has the potential to reduce recurrent hospitalisations, improving quality of life and promoting a greater sense of personal competence. Finally, this study aims to test an innovative and rigorously structured psychoeducational intervention, which could potentially represent a valuable complementary therapeutic option in acute psychiatric services for adolescents. The adoption of an integrated and participatory approach is a significant step towards the development of more effective, personalised treatment pathways aimed at preventing chronicity in the early stages of mental illness in young people.
