# Supplementary material for: Study protocol for a pilot randomized controlled trial on the feasibility and preliminary efficacy of an integrated psychoeducational intervention for transition-age youths in acute psychiatric settings
Source: Front Psychiatry. 2026 Feb 13;17:1768016. doi: 10.3389/fpsyt.2026.1768016 (PMC12946135; doi:10.3389/fpsyt.2026.1768016)
Supplement: Supplementary file 1 [file DataSheet1.pdf]

AZIENDA OSPEDALIERO-UNIVERSITARIA
DELLE MARCHE
UNIVERSITÀ POLITECNICA DELLE MARCHE
OSPEDALI RIUNITI di ANCONA
DIPARTIMENTO DI SCIENZE NEUROLOGICHE
CLINICA di PSICHIATRIA

Modulo 1
"Emozioni di base"

Guida per gli operatori

All'interno della guida il *corsivo* viene utilizzato per indicare quello che l'operatore deve pronunciare.

Le emozioni sono fenomeni complessi e transitori che coinvolgono la psiche e il corpo e determinano comportamenti funzionali o disfunzionali. Le emozioni accadono in risposta ad eventi esterni o interni, in quanto possono essere scatenate sia da particolari situazioni che dalla produzione di pensieri e ricordi.

Lo psicologo statunitense Paul Ekman [1] ha definito le cosiddette emozioni di base (o "primarie"), che si manifestano in tutti gli esseri umani, indipendentemente dal contesto socio-culturale di appartenenza.

Le emozioni primarie sono: gioia, tristezza, rabbia, disgusto, paura. La combinazione delle emozioni primarie genera emozioni secondarie, fra le quali riconosciamo la vergogna, la delusione, il rimorso, la speranza e molte altre.

Tutte le emozioni hanno un correlato nella mimica facciale e nell'espressività, permettendoci, in tal modo, di comunicare all'esterno il nostro stato d'animo attuale.

Tutte le emozioni necessitano di essere adeguatamente regolate, per non determinare eccessiva sofferenza o comportamenti disfunzionali e maladattivi.

Le persone che presentano difficoltà nella regolazione emotiva, nel momento in cui esperiscono emozioni percepite come poco tollerabili, possono mettere in atto comportamenti non adattivi come ad esempio gesti autolesivi o aggressivi, autodiretti o eterodiretti.

Spesso può accadere che, durante l'adolescenza, si manifesti una difficoltà nel riconoscere le emozioni e nel limitarne il loro impatto, con la conseguenza di aumentare le probabilità di agire in modo maladattivo.

Quindi, nell'incontro odierno, ci si focalizzerà sul riconoscimento delle emozioni primarie e sulla loro funzione. Verrà, inoltre, introdotto il modello "ABC" di Ellis, che sarà discusso con

1

i partecipanti, anche alla luce delle proprie esperienze personali. Tale modello è utile per aiutare il paziente a prendere coscienza di come si sviluppano i propri vissuti emotivi, a partire da un evento che accade nel "qui ed ora".

Gli **obiettivi** dell'incontro sono i seguenti:

1. imparare a riconoscere le emozioni
2. saper collegare le emozioni ai pensieri corrispondenti
3. saper leggere le esperienze emotive personali tramite il modello "ABC"

L'operatore, inoltre, deve favorire l'intervento di tutti i membri del gruppo durante l'incontro, in un'ottica attiva e partecipata.
Al fine di incoraggiare gli interventi e le riflessioni di tutti i partecipanti, sarà necessario che l'operatore mantenga, durante tutta la durata degli incontri, un linguaggio semplice e chiaro. Fondamentale è coinvolgere tutti i partecipanti con domande dirette.

Durante l'incontro può accadere che vengano toccati argomenti sensibili. Il conduttore deve cercare di evitare discussioni dai toni accesi, cercando di presentare gli argomenti con toni pacati, accoglienti e tranquilli.

La durata degli incontri varia dai 60 ai 90 minuti, sulla base delle domande poste dai partecipanti.

Al termine degli incontri il conduttore, aiutato dal co-conduttore, consegnerà l'homework ai partecipanti e risponderà ad eventuali richieste.

Slide 1
Introduzione dell'intervento

Benvenuti al primo incontro!
Questo ciclo di incontri è pensato per dare un'idea delle principali tematiche psicologiche che possono esserci utili a capire meglio cosa ci accade e come gestire in maniera funzionale alcune situazioni critiche.

Condurre un giro di presentazione di tutti i partecipanti.

Slide 2
Le emozioni di base

Prima di cominciare, facciamo questa premessa: questi incontri non sono le lezioni frontali che avete imparato a conoscere a scuola, ma momenti di confronto, facilitati da noi conduttori.

È importantissimo, pertanto, che ognuno di voi si senta libero di dire la propria opinione ed apportare un contributo alle riflessioni che faremo insieme.

In particolare, in questo primo incontro, capiremo insieme cosa sono le emozioni, qual è la loro funzione e come si esprimono in ognuno di noi.

Slide 3
Brainstorming

Sapete cos'è un brainstorming? (favorire gli interventi)

Un brainstorming è una sorta di intervista di gruppo, nella quale viene sfruttato il gioco creativo dell'associazione di idee. La finalità è far emergere diverse possibili alternative, in vista della soluzione di un problema o di una scelta da compiere.

In questo caso, facciamo un brainstorming su questa domanda:

Cos'è, per te, un'emozione?

In questa fase iniziale è importante favorire gli interventi, ribadendo che non esistono risposte giuste o sbagliate e che tutte le riflessioni o le parole-chiave sono utili alla riflessione collettiva.

Dopo aver raccolto le riflessioni di tutti (appuntandole su una lavagna), *proviamo a riflettere insieme sulla definizione scientifica del concetto di emozione.*

Slide 4
Definizione

Le emozioni sono stati mentali e fisiologici associati ad uno stimolo interno o esterno che producono una modificazione psicologica, fisiologica, motoria ed espressiva.

Che ne pensate? (favorire gli interventi)

Slide 5
Definizione

Il riconoscimento e la regolazione delle emozioni, giocano un ruolo importante per la nostra Salute.

Slide 6
Caratteristiche

Le emozioni compaiono prima del linguaggio e del pensiero come forma di comunicazione (pensate al pianto di un neonato affamato).

Esse hanno una funzione adattiva, sono cioè fondamentali per la nostra sopravvivenza (pensate ad una persona sul ciglio della strada che sta per attraversare le strisce pedonali senza un minimo di ansia...che fine farebbe?)

Slide 7
Caratteristiche

Le emozioni di base o primarie vengono esperite indipendentemente dal contesto sociale e culturale, mentre l'espressione dell'emozione, varia in base al contesto culturale.

Le emozioni, inoltre, hanno componenti neurobiologiche che si attivano in risposta ad uno stimolo.

Slide 8
Le emozioni di base

Ora vediamo nello specifico quali sono le cosiddette emozioni primarie (o di base):
gioia, tristezza, rabbia, paura, disgusto.

Avete visto il film "Inside Out"? (favorire gli interventi)

Slide 9
Inside out (2015)

Vediamo ora uno spezzone del film "Inside Out", che ci chiarisce il ruolo e l'importanza di ogni emozione.

Slide 10
Gioia

La gioia ci segnala che uno scopo è stato raggiunto, che abbiamo ottenuto qualcosa.
Vi ricordate l'ultima volta in cui avete provato gioia/felicità? (favorire gli interventi)

Slide 11
Tristezza

La tristezza, al contrario, ci segnala che abbiamo perso qualcosa o fallito in un obiettivo.
Vi torna?

Provate a pensare all'ultima volta in cui avete provato tristezza (favorire gli interventi)

Slide 12
Rabbia

La rabbia ci segnala che abbiamo subito un danno o un torto, oppure che un nostro importante obiettivo presenta un ostacolo che, al momento, non ci permette di raggiungerlo.

Slide 13
Paura/Ansia

Paura e ansia...si dicono "emozioni sorelle".

Proviamo a pensare come mai siano simili, cosa ci stanno dicendo?

Quale potrebbe essere la differenza tra le due?

La paura riguarda un'attivazione che ci segnala un pericolo reale che abbiamo davanti, mentre l'ansia, ci segnala una minaccia che potrebbe essere anche solo immaginata e/o futura.

Se sto assistendo ad una scossa di terremoto quale delle due emozioni proverò? (paura)
Se invece penso che domani non riuscirò a parlare durante l'interrogazione? (ansia)

Slide 14
Disgusto

Questa emozione, molto importante per la nostra evoluzione, ci segnala oggetti, situazioni, o sostanze potenzialmente dannosi per la nostra sopravvivenza.

Quando si attiva il vostro disgusto? (Favorire gli interventi)

Slide 15
Emozioni piacevoli e spiacevoli

E' importante ricordare che non esistono emozioni positive o negative.
E' naturale sperimentare tutte le emozioni, sia piacevoli che spiacevoli.

Ricorda che:

- *tutte le emozioni sono utili*
- *ci guidano nell'esplorazione dell'ambiente e nelle decisioni*
- *informano gli altri sui nostri stati interni*

Slide 16
Emozioni piacevoli e spiacevoli

Quali di queste emozioni sono piacevoli da sperimentare e quali spiacevoli, per voi? (favorire gli interventi)

Slide 17
Componenti dell'emozione

Come dicevamo all'inizio, le emozioni sono formate da diverse componenti, vediamole ora nel dettaglio:

- *cognitiva (pensiero)*
- *fisiologica (attivano il corpo)*
- *motivazionale (spingono all'azione)*
- *espressivo-motoria (attivano l'espressività facciale e i movimenti corporei)*
- *esperienziale (sono riferite ad una situazione/contesto specifici)*

Slide 18
Sistema limbico

Questa immagine vi da un'idea della collocazione del sistema limbico, un complesso di strutture encefaliche che possiede un ruolo chiave nelle reazioni emotive, nelle risposte comportamentali, nei processi di memoria e nell'olfatto.

Slide 19
Giochiamo con le emozioni di base

Ora ci mettiamo in gioco!

- *Ognuno di voi pescherà una "carta".*
- *In ogni carta è indicata un'emozione di base ed ognuno di voi dovrà interpretarla, senza parlare, attraverso l'espressività, i movimenti corporei e la postura.*
- *Gli altri dovranno indovinare di quale emozione si tratta.*

Pronti? (distribuire le carte)

Slide 20
Funzione delle emozioni

Potrebbe essere utile considerare le emozioni come delle "notifiche" del nostro cellulare.

Aprendole, troveremo dei messaggi!

In particolare, le emozioni ci segnalano:

- *se una situazione desiderata o temuta si realizza*
- *ci informano sull'andamento del nostro rapporto con l'ambiente rispetto ai nostri scopi*
- *ci dicono se c'è qualcosa da cambiare o da mantenere (valore adattivo delle emozioni).*

Slide 21
Il modello "ABC"

Ora esploriamo insieme un modello che ci aiuta a fare chiarezza su ciò che viviamo.

Questo modello è come un "mobile" con dei cassetti, che ci aiuta a fare "ordine" nelle nostre esperienze.

Il modello "ABC" è semplice ed intuitivo e ci aiuta a comprendere quale legame c'è tra i nostri pensieri e le nostre emozioni, a partire dagli eventi e dalle situazioni che viviamo quotidianamente.

Vediamolo nel dettaglio.

Slide 22
Il modello "ABC"

- *A sta per evento attivante (activating event) ovvero la situazione che stiamo vivendo nel "qui ed ora".*
- *B sta per credenze (beliefs) ovvero i pensieri che ci passano per la testa durante una*

situazione specifica.

- *C sta per conseguenze (consequences) ovvero le emozioni che proviamo e i comportamenti che mettiamo in atto, in reazione all'emozione che stiamo sperimentando.*

Slide 23
Il modello "ABC"

Vediamo ora le 3 domande da porsi per completare un ABC...

- *Qual era la situazione nella quale il problema si è manifestato?* (TROVARE A)
- *Come mi sono sentito/a?* (TROVARE C)
- *Quali frasi mi sono passate per la testa in quel momento? Cosa mi sono detto/a? Qual è stato il primo pensiero?* (TROVARE B)

Slide 24
Il modello "ABC" (esempio)

Vediamo ora degli esempi pratici, letti attraverso il modello "ABC".

- *La prof mi chiama alla lavagna*
- *Se non so rispondere, farò una figuraccia*
- *Ansia*

Slide 25

Il modello "ABC" (esempio)

- *Un compagno a cui ho prestato dei soldi, non ha intenzione di ridarmeli*
- *Si è approfittato della mia gentilezza!*
- *Rabbia*

(stimolare la riflessione soggettiva su ogni ABC indicato in tabella)

Slide 26
Il modello ABC

Da cosa dipende, allora, l'emozione? (favorire gli interventi)

Slide 27

Modello ABC

Le nostre emozioni non dipendono tanto da quello che ci accade, dalle situazioni o dagli eventi, quanto più dai nostri ***pensieri,*** *dalle frasi che ci diciamo mentre viviamo una specifica situazione attivante.*

*Le nostre emozioni dipendono, quindi, dall'****interpretazione*** *che noi facciamo dell'evento e non dall'evento in sé.*

Slide 28
Homework

Durante la settimana, prova a completare l'ABC con alcuni episodi che ritenete particolarmente attivanti, indicando:

*a) l'****episodio o la situazione*** *in cui ti trovavi*
*b) l'****emozione*** *che hai provato*
c) ***il pensiero*** *che ti è passato per la testa*

(consegnare le schede di lavoro, rispondendo ad eventuali richieste)

Modulo 2
"Ansia e tristezza"

Guida per gli operatori

Le emozioni sono fenomeni complessi e transitori che coinvolgono la psiche e il corpo e determinano comportamenti funzionali o disfunzionali.
Le emozioni si verificano in risposta ad eventi interni o esterni, perché possono essere scaturite da situazioni reali, ma anche da pensieri e ricordi.

Tutte le emozioni hanno un correlato nella mimica facciale e nell'espressività, permettendoci, in tal modo, di comunicare all'esterno il nostro stato d'animo attuale.

Tutte le emozioni, necessitano di essere adeguatamente regolate, per non determinare eccessiva sofferenza o comportamenti disfunzionali. Tra queste troviamo l'ansia e la tristezza, emozioni fisiologiche aventi una specifica funzione adattiva, che possono divenire problematiche, se non adeguatamente regolate. Le persone che presentano difficoltà nella regolazione emotiva di ansia e tristezza, infatti, possono mettere in atto comportamenti non adattivi.

Spesso può accadere che, da adolescenti, si manifesti una difficoltà nel riconoscere le emozioni e nel limitarne il loro impatto, con la conseguenza di aumentare le probabilità di agire in modo impulsivo o disfunzionale.

Dunque, nel seguente incontro, si comprenderà la funzione delle emozioni di ansia e tristezza e verranno forniti gli strumenti per riconoscere quando esse si manifestano in modo fisiologico o patologico.

Gli obiettivi dell'incontro sono i seguenti:
1. imparare a riconoscere ansia e tristezza
2. saper collegare ansia e tristezza ai pensieri corrispondenti
3. comprendere quando ansia e tristezza diventano patologiche o disfunzionali
4. saper leggere le esperienze emotive personali di ansia e tristezza tramite il modello "ABC".

Slide 1

Benvenuti al secondo incontro!

Nel modulo precedente ci siamo dedicati alle emozioni di base, abbiamo esplorato quali sono le loro funzioni ed introdotto il modello "ABC".

Ricordate?

Slide 2
Ansia e tristezza

Oggi ci occuperemo, nello specifico, di due emozioni primarie: ansia e tristezza.

Slide 3
Definizione (recap)

Abbiamo già definito la scorsa volta le emozioni come stati affettivi intensi, di breve durata, che possono avere una causa interna o esterna, un contenuto cognitivo e comportamentale ed hanno la funzione di riorientare l'attenzione.

Slide 4
Componenti (recap)

Le componenti delle emozioni sono:
- *situazioni attivanti*
- *credenze (pensieri)*
- *vissuto corporeo*
- *predisposizione all'azione*
- *vantaggio evolutivo (sono utili)*
- *inizio, durata e fase di attenuazione*

Slide 5
Brainstorming (ansia)

Condividiamo tutto ciò che ci viene in mente sul concetto di "Ansia" (immagini, canzoni, colori, ricordi, parole, situazioni, esperienze…)

Appuntare le riflessioni che emergono dal brainstorming su una lavagna.

Slide 6
Ansia e paura

Ansia e paura si definiscono emozioni "sorelle", ma mentre nella paura l'oggetto temuto è realmente davanti a noi (o l'evento si sta verificando in quel momento), nell'ansia si anticipa una minaccia ipotetica.

Pensate ad un terremoto in corso, quale sarà la vostra emozione? (paura)

Pensate ora alla possibilità che possiate essere interrogati a scuola, quando non siete molto preparati. Che emozione potrete provare in questo caso? (ansia)

Slide 7
Funzione dell'ansia

La funzione dell'ansia è quindi quella di anticipare una possibile minaccia e preparare il corpo e la mente a reagire ad essa.

Pensate ad una persona che sta aspettando l'autobus per un importante colloquio di lavoro. Se l'autobus è in ritardo, come potrebbe sentirsi?

Questi sono alcuni esempi di pensieri che possono elicitare l'ansia:

Riuscirò a parlare all'interrogazione?
Prenderò l'autobus in tempo?
Mi inviteranno alla festa?

Slide 8
Funzione dell'ansia

La funzione dell'ansia è quella di preparare corpo e mente alla risposta di attacco o fuga e di aumentare lo stato di vigilanza sulla potenziale minaccia in atto.

Slide 9
La curva dell'ansia

Abbiamo sottolineato, nel modulo precedente, che tutte le emozioni sane hanno un inizio, un picco massimo ed una fase di attenuazione.

Dal grafico, potete notare che anche l'ansia si comporta come una curva gaussiana e che, una discreta attivazione ansiosa, è utile e necessaria per prepararsi adeguatamente alla performance o al compito da svolgere.

Slide 10
Sintomi dell'ansia

Ognuno può pensare a come si sente quando è in ansia (favorire gli interventi)
Cosa sentite nel corpo?

I sintomi dell'ansia sono i seguenti:
- *irrequietezza, sentirsi tesi, con i "nervi a fior di pelle"*
- *facile affaticamento*
- *difficoltà di concentrazione (o vuoti di memoria)*
- *irritabilità*
- *tensione muscolare*
- *alterazioni del sonno*
- *tachicardia*
- *sudori*

Slide 11
Disturbi d'ansia

Quando, secondo voi, l'ansia diventa un problema? (favorire gli interventi)

Slide 12
Disturbi d'ansia

L'ansia diventa un problema quando:
- *inizia ad interferire con le nostre attività quotidiane*
- *ci impedisce di raggiungere i nostri obiettivi.*
- *crea sintomi psico-fisici che durano nel tempo*

Esempio: vorrei uscire con gli amici, ma l'ansia mi costringe a rimanere a casa.

Slide 13
Disturbi d'ansia

Quando si diventa eccessivamente ansiosi, accade inoltre che la capacità di risolvere problemi diminuisce, perchè l'ansia riduce la capacità di pensare ed agire in modo lucido e ragionevole.

Slide 14
Brainstorming (tristezza)

Ora passiamo alla seconda emozione di cui vogliamo occuparci oggi.

Condividiamo tutto ciò che ci viene in mente sul concetto di "Tristezza".
(immagini, canzoni, colori, ricordi, parole, situazioni, esperienze…)

Slide 15
Funzione della tristezza
- *permette all'organismo di ritrovare il proprio equilibrio*
- *protegge la persona nei momenti di maggiore vulnerabilità*

Slide 16
Funzione della tristezza
- *permette di recuperare le forze fisiche o psicologiche*
- *richiama l'attenzione sull'accudimento*

(ad esempio: quando qualcuno vicino a noi piange, siamo tentati di avvicinarci e fornire supporto)

Slide 17
Inside out

Ora vediamo uno spezzone del film "Inside Out" che sottolinea l'importanza della tristezza.

Slide 18
Disturbi depressivi

Quando allora, secondo voi, la tristezza diventa un problema? (favorire gli interventi)

Slide 19
Disturbi depressivi

I sintomi della depressione sono i seguenti:

- *umore depresso per la maggior parte del giorno, quasi tutti i giorni*
- *perdita di interesse o piacere per le attività che prima si trovavano piacevoli*
- *significativo cambiamento nel peso (perdita o aumento)*
- *insonnia o ipersonnia*

Slide 20
Disturbi depressivi

- *agitazione o rallentamento psicomotorio*
- *faticabilità o mancanza di energia*
- *sentimenti di autosvalutazione o di colpa eccessivi o inappropriati*
- *ridotta capacità di pensare o concentrarsi*
- *pensieri ricorrenti di morte*

Slide 21
Disturbi depressivi

In termini semplici, potremmo dire che la *depressione è una tristezza profonda protratta nel tempo, caratterizzata da sentimenti e pensieri autosvalutanti, mancanza di speranza e senso di vuoto.*

Slide 22
Sintomi depressivi

Come si manifesta, invece, la depressione nella nostra mente?

I sintomi cognitivi della depressione sono i seguenti:

- *demotivazione*
- *pensieri negativi*
- *problemi di concentrazione*
- *difficoltà di memoria*
- *visione pessimistica della realtà*
- *senso di scarso valore e di fallimento*

Alcuni pensieri tipici della depressione possono essere: sono un fallito/a, non valgo niente, non guarirò mai, è tutta colpa mia, niente sarà più come prima".

Slide 23
Indicatori di emozioni problematiche

Come già anticipato all'interno del primo modulo, possiamo considerare alcuni indicatori per comprendere quando le nostre emozioni necessitano di un intervento di tipo medico.

Gli indicatori di emozioni problematiche sono i seguenti:

- *intensità*
- *sproporzione rispetto alla situazione*
- *impedimento nel vivere la solita quotidianità*
- *lunga durata*

Slide 24
Homework

Prima di salutarci, vogliamo continuare ad allenarci con le cose apprese.

Durante la settimana, prova a completare l'ABC con alcuni episodi che ritieni particolarmente attivanti e segna nelle tre colonne:

a) l'episodio o la situazione in cui ti trovavi

b) l'emozione che hai provato (ansia o tristezza)

c) il pensiero che ti è passato per la testa

(consegnare la scheda di lavoro e rispondere ad eventuali richieste)

Intervento Psicoeducativo

Modulo 3

“Esperienze insolite”

Guida per gli operatori

All’interno della guida il *corsivo* viene utilizzato per indicare quello che l’operatore deve pronunciare.

Slide 1

Introduzione all’incontro

Oggi parleremo delle cosiddette esperienze insolite. Nel corso di una psicosi si sperimenta una alterazione del giudizio di realtà, eventualmente associati alla presenza di idee deliranti o allucinazioni. Andremo a sviscerare che cosa significa questi termini e come riconoscerli.

Per non fare confusione, cercherò di seguire una scaletta degli argomenti da trattare. Alla fine di questo incontro, se vi sono punti importanti di cui non abbiamo parlato, li vedremo insieme.

Prima di cominciare vorrei chiedervi se avete domande da fare sugli argomenti di cui abbiamo parlato nello scorso incontro.

Volete chiedermi qualcosa prima di cominciare?

Sollecitare l’intervento di tutti.

Invitare a fare domande.

Rispondere ad eventuali domande o, se non si conosce la risposta, prendere appunti, assicurando i presenti che se ne parlerà la prossima volta.

Slide 2

Obiettivi dell’incontro

Presentare gli obiettivi dell’incontro.

Gli obiettivi dell’incontro saranno:

1. *Imparare cosa sono le «esperienze insolite»,*
2. *Imparare a riconoscerle,*
3. *Imparare come far fronte a queste «esperienze».*

Slide 3

Esempio di "Esperienze insolite"

Descrivere e commentare il caso clinico.

Slide 4

Dissociazione

Con dissociazione si intende un meccanismo di difesa con cui alcuni elementi dei processi psichici rimangono "disconnessi" o separati dal restante sistema psicologico dell'individuo. Si verifica durante i disturbi d'ansia, i disturbi dell'umore, a seguito di traumi.

Vi è mai capitato di provare questa sensazione?

Sollecitare l'intervento di tutti.

Invitare a fare domande.

Slide 5

Derealizzazione

La derealizzazione si caratterizza per il sentimento di irrealtà, come se «nulla fosse reale». Si verifica durante i disturbi d'ansia ed i disturbi dell'umore.

Vi è mai capitato di provare questa sensazione?

Sollecitare l'intervento di tutti.

Invitare a fare domande.

Slide 6

Depersonalizzazione

La depersonalizzazione si caratterizza per una perdita della percezione di sé stessi. Può inoltre capitare che il paziente veda sé stesso dall'alto o dall'esterno mentre svolge le attività quotidiane. Si verifica durante i disturbi d'ansia ed i disturbi dell'umore.

Vi è mai capitato di provare questa sensazione?

Sollecitare l'intervento di tutti.

Invitare a fare domande.

Slide 7

Sintomi degli episodi psicotici

I disturbi psicotici rappresentano una delle grandi classi delle patologie psichiatriche. La schizofrenia ha rappresentato da sempre la malattia regina di questa branca della medicina. La schizofrenia però non è l'unico disturbo facente parte di questa classe, ma sono presenti altri disturbi: disturbo delirante, disturbo schizofreniforme, bouffe delirante e il disturbo schizoaffettivo. Si caratterizzano per la presenza dei seguenti sintomi:

- *Sintomi positivi: ideazione delirante, allucinazioni (principalmente di tipo uditivo), alterazioni della forma del pensiero,*
- *Sintomi negativi: affettività appiattita, ritiro sociale, difficoltà relazionali.*

La presenza di sintomi di carattere psicotico però non indica necessariamente la presenza di un disturbo psicotico, i sintomi psicotici possono infatti essere rinvenuti nel contesto dei disturbi dell'umore o nei disturbi di personalità, ad esempio si possono avere episodi depressivi con sintomi psicotici o durante le riacutizzazioni del disturbo bipolare.

Qualcuno di voi soffre di questo tipo di disturbo? Accetteresti di condividere le tue esperienze?

Rispondere ad eventuali domande o, se non si conosce la risposta, prendere appunti, assicurando i presenti che se ne parlerà la prossima volta.

Slide 8

Allucinazioni e illusioni

Qualcuno di voi saprebbe dirmi che cos'è un'allucinazione? Le avete mai sperimentate?

È importante sottolineare che le allucinazioni sono dei fenomeni abbastanza frequenti, anche nella popolazione "sana". Se uno dei pazienti acconsente a condividere un suo vissuto durante il gruppo, il conduttore deve mantenere un atteggiamento "normalizzante", per incoraggiare anche gli altri a raccontare loro esperienze. Si potrebbe dire: "Succede a tutti di sentire delle voci, rumori, suoni che in realtà non provengono realmente dall'esterno. Questo succede principalmente nei momenti di stanchezza, preoccupazione ma soprattutto durante le fasi di dormiveglia. Ad esempio, capita di sentirsi chiamati per nome quando si cammina soli in una strada buia. Tale atteggiamento dovrebbe essere comunque mantenuto se nessuno dei pazienti accetta di condividere la sua esperienza.

Rispondere ad eventuali domande o, se non si conosce la risposta, prendere appunti, assicurando i presenti che se ne parlerà la prossima volta.

Le allucinazioni sono delle alterazione della percezione della realtà esterna. Si tratta della percezione di qualcosa non presente nell'ambiente, sperimentabile mediante uno dei cinque sensi (raramente possono essere più sensi). In genere nel contesto delle malattie psichiatriche, le allucinazioni più frequenti sono quelle uditive, si parla infatti spesso di voci.

Ciò che differenzia un'allucinazione da una illusione è la presenza dell'oggetto esterno. Nelle illusioni, infatti, è la percezione di qualcosa reale ad essere alterata. Ad esempio, un oggetto può essere percepito più grande o più piccolo del normale.

Se uno dei pazienti acconsentisse a descrivere la sua esperienza con le voci, si potrebbe chiedere:

- *Che cosa ti dicevano le voci?*
- *Si trattava di un uomo o di una donna?*
- *Come ti sentivi in quel momento?*
- *Quando hai sentito le voci, dove ti trovavi? Con chi eri? Cosa facevi?*
- *Le hai sentite più volte? In quali altri contesti?*
- *Ci sono momenti in cui peggiorano? Quando sei sotto stress?*
- *Quando invece migliorano?*
- *Che fai quando senti le voci?*
- *Secondo te erano voci reali o originano dalla tua mente?*
- *Ti è mai capitato di pensare le stesse cose che ti dicono le voci?*

Slide 9

Allucinazioni uditive

Non tutte le allucinazioni sono patologiche. In particolare, esistono le allucinazioni ipnagogiche ed ipnopompiche, che si verificano rispettivamente durante la fase dell'addormentamento e durante il risveglio. Esistono anche le allucinazioni che si verificano durante l'uso di sostanze, le quali però svaniscono una volta che l'effetto della droga termina. Le allucinazioni uditive sono un indice di profonda sofferenza psicologica e tendono a peggiorare sotto stress. Lo stress è infatti una fattore precipitante per questo tipo di sintomatologia. A volte sono un modo per far fronte alla solitudine o all'isolamento prolungato. Succede infatti che persone isolate da lungo tempo, possano iniziare a esperire questo tipo di allucinazioni.

Slide 10

Allucinazioni uditive

Distrarsi aiuta a ridurre le presenza di tali dispercezioni. Ridurre o evitare i fattori stressanti, infatti, aiuta a prevenire il peggioramento di questa sintomatologia. È fondamentale parlare con qualcuno di cui ci si fida, questo aiuta ad attenuare la presenza delle voci e a riconoscerle come manifestazione derivante dal nostro pensiero.

Vi è mai successo di avere notare questi cambiamenti nella presenza delle allucinazioni?

Rispondere ad eventuali domande o, se non si conosce la risposta, prendere appunti, assicurando i presenti che se ne parlerà la prossima volta.

Sulla base di quello che abbiamo detto finora, qual è lo scopo di ridurre le voci? Quali potrebbero essere le strategie da attuare?

Sollecitare l'intervento di tutti.

Invitare a fare domande.

Rispondere ad eventuali domande o, se non si conosce la risposta, prendere appunti, assicurando i presenti che se ne parlerà la prossima volta.

Ridurre le voci è fondamentale per ridurre il dolore che queste provocano e favorire la comunicazione con gli altri.

Slide 11

Come ridurre le voci?

Le strategie che possiamo attuare per la ridurre le voci sono:

1. Distrarsi ed impegnarsi in attività piacevoli (canticchiare, parlare con te stesso, ascoltare musica, pregare, meditare, usare un mantra, dipingere, camminare all'aria aperta, chiamare un amico, fare esercizio fisico, usare un cd di rilassamento, fare yoga, fare un bagno caldo, chiamare il proprio psichiatra, frequentare un centro diurno, guardare la tv, fare un cruciverba o un puzzle, giocare ad un gioco al computer, provare un nuovo hobby),

2. Scrivere o disegnare le voci, dialogare con le voci, focalizzarsi su quelle positive,

3. Dare alle voci uno spazio di dieci minuti in uno specifico orario ogni giorno,

4. Ricorda a te stesso che nessun altro può sentire la voce, usa una spiegazione normalizzante

5. Usa risposte razionali per ridurre la rabbia,

6. Elencare le prove contro il contenuto delle voci,

7. Riconoscere i fattori stressanti per poterli evitare o prevenire,

8. Assumere correttamente la terapia farmacologica,

9. Rimanere in contatto con lo psichiatra.

Slide 12

Delirio o idea delirante

Cosa si intende secondo voi con il termine delirio?

Sollecitare l'intervento di tutti.

Invitare a fare domande.

Con il termine delirio si intende un'idea o una convinzione completamente falsa con cui nessun altro può essere d'accordo e a cui qualcuno crede fermamente.

Vi è mai capitato di avere alcune di queste idee strane? Cosa pensavi che stesse succedendo?

Rispondere ad eventuali domande o, se non si conosce la risposta, prendere appunti, assicurando i presenti che se ne parlerà la prossima volta.

Se uno dei pazienti ha dato il consenso a descrivere cosa gli è successo, fargli raccontare l'evento. Si potrebbe iniziare dicendo: "Parlando con i medici, XX ha riferito di alcune situazioni spiacevoli che lo fanno e lo hanno fatto soffrire. Ha accettato di condividere con voi queste cose, per approfondirle, capire quanto lo fanno stare male e cosa si può fare per affrontare il problema. Siamo molto grati a XX per queste informazioni". Successivamente si potrebbe aggiungere: "Noi medici, parlando del suo problema, ci siamo resi conto di non aver capito bene se quello che accade e che altera il suo rapporto con l'altro sia reale oppure origini da un malinteso; perciò, sarebbe utile che parlassimo di questo per approfondire l'argomento e ascoltare la vostra opinione". In questa fase, che ha il ruolo di "normalizzare" queste esperienze non si dovrebbe intervenire con domande di approfondimento e si evita anche che gli altri degenti possano dare giudizi o fare domande: eventuali domande sono da rimandare ad un successivo momento. La normalizzazione è fondamentale per incoraggiare tutti i pazienti a non spaventarsi ed aiutarli a comunicare. Ad esempio, "XX ci ha riferito che, stressato da una serie di eventi della sua vita, esagerando i segnali provenienti dall'esterno, ha pensato che i vicini di casa volessero ucciderlo. In realtà è abbastanza frequente che ognuno di noi, sotto stress, trasformi le sue paure in realtà: questo si può definire come pensiero psicotico".

Slide 13

Esempi di delirio

Ecco alcuni esempi di pensiero delirante:

- *Pensare che una forza o un'altra persona controlli i suoi pensieri e/o le azioni,*
- *Pensare che ciò che vede o legge nasconda un messaggio segreto indirizzato a lui,*
- *Pensare di essere un persona speciale molto diversa da quella che è o di avere capacità veramente straordinarie.*

Discutere su queste esperienze.

Sollecitare l'intervento di tutti.

Invitare a fare domande.

Il conduttore chiede a ogni ricoverato di porre domande di approfondimento al paziente che ha descritto la sua esperienza per ricondurre l'esperienza psicotica a un modello di disturbo psicologico e di malfunzionamento mentale basato sul concetto di vulnerabili allo stress.

Il ruolo del conduttore è quello di facilitare, senza interpretare, indirizzare o far trasparire le proprie opinioni al riguardo, domande su:

- contesto;
- situazioni che peggiorano o migliorano il pensiero psicotico;
- stato d'animo che precede l'insorgenza del pensiero psicotico;
- conseguenze emotive del fenomeno;
- azioni che il paziente compie per gestire questo problema.

Le domande pertinenti vengono sottoposte direttamente all'attenzione del paziente; quelle incomplete, che si avvicinano agli obiettivi del lavoro, sono completate o riformulate e successivamente rivolte al paziente. Se il lavoro svolto è insufficiente, cioè le risposte ottenute sono vaghe, non pertinenti, poco utili a chiarire il "processo" del pensiero psicotico e/o la relazione con gli eventi che favoriscono tale fenomeno, il conduttore o il co-conduttore tendono far emergere risposte relative ai seguenti interrogativi:

- quando esattamente il paziente ha messo a fuoco il pensiero in questione?
- in quel momento, che cosa stava accadendo nella vita del paziente, ovvero quali fattori stressanti erano in atto?
- qual era lo stato d'animo del paziente prima che maturasse il pensiero, cioè il paziente si sentiva preoccupato, spaventato, confuso, triste, invidioso, deluso eccetera?
- da quali fatti, esattamente, il paziente ha capito quello che ha raccontato?
- si possono fare ipotesi alternative per spiegare quello che è accaduto? se ad esempio paziente ha capito dallo sguardo arrabbiato del vicino che questi lo avrebbe ucciso, è possibile spiegare lo sguardo arrabbiato con fatti indipendenti dal paziente (ad esempio vicino era arrabbiato con la propria moglie)?
- il paziente ne ha parlato a qualcuno, ha chiesto consigli o pareri?

In questa fase il conduttore, riproponendo il racconto del paziente e utilizzando le stesse parole (per esempio: «Ero sicuro che il vicino di casa volesse uccidermi») invita a turno ogni ricoverato a spiegare perché una persona si convince della veridicità del tanto che la sua vita ne viene completamente trasformata, fino al punto di essere accompagnata in ospedale per cure non richieste dallo stesso paziente. Le risposte che ci attendiamo sono:

- XX è molto vulnerabile allo stress;
- XX ha una malattia/disturbo che si manifesta con questo sintomo;

- la personalità, il carattere di XX, il modo in cui funziona la sua mente, lo portano a convincersi di cose che non esistono.

Slide 14

Cause dello sviluppo di episodi psicotici

Sulla base di quello che abbiamo detto finora, quali sono le cause dello sviluppo di sintomi psicotici?

Sollecitare l'intervento di tutti.

Invitare a fare domande.

Le principali cause che possono portare allo sviluppo di sintomi psicotici sono: un forte stress e l'uso di sostanze stupefacenti.

Slide 15

Strategie per affrontare il problema

Come possiamo affrontare il problema?

Sollecitare l'intervento di tutti.

Invitare a fare domande.

Le risposte sono ovvie e in linea con quelle già descritte per le allucinazioni:

- *farmaci antipsicotici per favorire quelle condizioni mentali che consentono di restituire al paziente la capacità di riconoscere le proprie paure distinguendole dalla realtà (ricordiamo che il termine psicosi nel nostro lavoro è utilizzato per indicare la perdita di tale capacità della mente);*
- *contatto continuo con lo psichiatra per stabilire e verificare comportamenti efficaci;*
- *riconoscimento dei segni precoci di crisi; riconoscimento e gestione dello stress.*

Slide 16

Homework

Spiegare il compito da svolgere entro la successiva seduta.

Prima di lasciarci, volete chiedermi qualcosa? Ci sono cose non chiare? Volete una copia del materiale che ho usato in questo incontro?

Sollecitare l'intervento di tutti.

Rispondere ad eventuali domande.

Ringraziare i presenti per la loro partecipazione, cercando di mettere in evidenza il contributo di ognuno.

Intervento Psicoeducativo

Modulo 4

"Dipendenze"

Guida per gli operatori

All'interno della guida il *corsivo* viene utilizzato per indicare quello che l'operatore deve pronunciare.

Slide 1

Introduzione all'incontro

Oggi parleremo delle varie forme di dipendenza, passando da quelle più conosciute alle nuove forme di dipendenza.

Per non fare confusione, cercherò di seguire una scaletta degli argomenti da trattare. Alla fine di questo incontro, se vi sono punti importanti di cui non abbiamo parlato, li vedremo insieme.

Prima di cominciare vorrei chiedervi se avete domande da fare sugli argomenti di cui abbiamo parlato nello scorso incontro.

Volete chiedermi qualcosa prima di cominciare?

Sollecitare l'intervento di tutti.

Rispondere ad eventuali domande o, se non si conosce la risposta, prendere appunti, assicurando i presenti che se ne parlerà la prossima volta.

Slide 2

Obiettivi dell'incontro

Presentare gli obiettivi dell'incontro.

Gli obiettivi dell'incontro saranno:

1. *Migliorare la consapevolezza sulle sostanze e sui rischi legati al loro utilizzo,*
2. *Imparare strategie alternative all'utilizzo della sostanza quando se ne sente il bisogno,*
3. *Acquisire consapevolezza sui danni legati all'uso eccessivo dei dispositivi digitali.*

Slide 3

Definizione di sostanza

Secondo voi cosa significa il termine sostanza o droga?

Sollecitare l'intervento di tutti.

Invitare a fare domande.

Slide 4

Definizione di sostanza

Con il termine droga si intendono tutte quelle sostanze (legali o illegali) che hanno la capacità di modificare lo stato di coscienza, il comportamento, le emozioni, i pensieri e, allo stesso tempo, di creare condotte di dipendenza o abuso.

Slide 5

Perché si fa uso delle sostanze?

Secondo voi perché si fa uso delle sostanze?

Sollecitare l'intervento di tutti.

Invitare a fare domande.

Slide 6

Perché si fa uso delle sostanze?

I motivi per cui inizialmente una persona può decidere di assumere una sostanza psicotropa possono essere tutti riconducibili a una qualche forma di piacere o all'evitamento di un disagio. In particolare, i motivi possono essere: rilassarsi, desiderare una gradevole eccitazione, svagarsi un po', disinibirsi e magari darsi coraggio, sentirsi maggiormente prestante, creativo o efficiente. Qualcuno potrebbe invece ricercare un temporaneo sollievo dall'ansia, dalla tristezza, dalla rabbia o da altri sentimenti indesiderati. Provare piacere è una capacità innata, mediata del cervello e comune agli umani. Così quando ad esempio mangi la torta che prediligi, quando raggiungi con impegno e sacrificio un obiettivo per te molto importante si attivano delle aree del cervello che costituiscono il cosiddetto della gratificazione e del piacere. Le cellule nervose di questo sistema rilasciano dopamina. Le sostanze psicotrope fanno invece un "trucco chimico", possono azionare questo meccanismo determinando il rilascio (o rallentando il riassorbimento) della dopamina nel sistema della gratificazione. In questo caso però, la disponibilità di dopamina sarà notevolmente aumentata e quindi sarà anche più intensa la stimolazione dei circuiti cerebrali.

Slide 7

Tipo di sostanze

Quali sostanze conoscete? Elencatele.

Sollecitare l'intervento di tutti.

Invitare a fare domande.

Slide 8

Tipo di sostanze

Le sostanze più utilizzate sono: Alcol, Cannabinoidi, Cocaina, Eroina ed altri oppiodi, Stimolanti (MDMA, anfetamine etc.), Allucinogeni (LSD, psilocibina), Caffè, Tabacco, Sostanze sintetiche.

Vi aspettavate di trovare sostanze "legali" come il caffè e l'alcol?

Sollecitare l'intervento di tutti.

Invitare a fare domande.

L'alcol genera una depressione a medio termine, portando anche ad un aumento delle quote ansiose, una destrutturazione del sonno, un discontrollo degli impulsi, un deterioramento cognitivo ed una slatentizzazione di sintomi psicotici. La cannabis genere quella che viene chiamata sindrome amotivazionale con conseguente apatia, depressione, alterazione del sonno, abulia, astenia. Può inoltre provocare uno switch maniacale nei pazienti bipolari o causare episodi psicotici. La cocaina, oltre ad avere degli effetti devastanti come l'eroina sul piano fisico, porta ad un aumento di ansia, aggressività, sintomi psicotici, deterioramento cognitivo, switch maniacali, alterazioni del sonno. L'eroina ha un quadro sovrapponibile a quello della cocaina come effetti a lungo termine. Gli allucinogeni e le droghe di sintesi possono causare l'insorgenza di episodi psicotici ed eventualmente indurre uno switch maniacale. I sintomi allucinatori inoltre possono persistere per lunghissimo periodo, generando dei fenomeni detti "flashback". Il caffè ha come effetto quello di alterare il ritmo del sonno, elemento fondamentale per una buona salute mentale. Può anche incrementare l'ansia e indurre attacchi di panico. Il tabacco non ha grandi rischi sul piano psichiatrico, ma sono soprattutto di natura fisica.

Le modalità comunicative del conduttore non devono assumere una connotazione "morale", né devono avere connotati paternalistici o atteggiamento da poliziotto. È necessario insistere in maniera esplicita che tali sostanze non devono essere assunte per motivi strettamente medici e liberi da aspetti morali.

Qualcuno di voi ha mai assunto queste sostanze? Hanno peggiorato i sintomi? Cosa avete provato?

Invitare a fare domande.

Slide 9

Quando insorge la dipendenza?

Secondo voi, quando si diventa dipendenti?

Sollecitare l'intervento di tutti.

Invitare a fare domande.

Slide 10

Quando insorge la dipendenza?

Il piacere è una qualità emotiva che ci motiva a ripetere ciò che lo ha procurato. Sembra infatti che, nel corso dell'evoluzione della specie, la selezione naturale abbia fatto emergere il piacere come meccanismo per incentivare comportamenti importanti per la sopravvivenza (come ad esempio mangiare, riprodursi, raggiungere un obiettivo). Il piacere e l'assunzione indotto droghe funge da incentivo (rinforzo) per le assunzioni successive, favorendo il ripetersi del consumo. Tuttavia, le conseguenze possono essere deleterie per la vita e col tempo possono ridurre, paradossalmente, la possibilità di provare piacere. Questo meccanismo determina (per motivi genetici, di genere, biologici, psicologici e ambientali) l'instaurarsi della dipendenza. Se l'assunzione delle sostanze continua, il cervello riduce la sua sensibilità alla dopamina quando la concentrazione diventa eccessiva: di conseguenza se ne produce meno, rendendo meno piacevoli tutte quelle attività che prima erano considerate gratificanti e piacevoli. L'unico modo per aumentare i livelli di dopamina sarà assumere la sostanze psicoattiva. Questo spinge la persona ad un progressivo disinteresse verso tutte le attività piacevoli della vita, portando allo sviluppo di sintomi depressivi. A questo si associa il fenomeno di tolleranza, per cui è necessario assumere dosi sempre maggiori della droga per ottenere lo stesso effetto. Tutto questo porta poi a problematiche familiari, lavorative, sociali, finanziarie, legali e mediche, che a loro volta spingono alla nuova assunzione di sostanza (generando così un circolo vizioso).

Slide 11

L'astinenza

Nel tempo, il piacere della sostanza tende a diminuire per effetto della tolleranza mentre potrebbero presentarsi delle sensazioni fisiche indesiderate (ad esempio nausea, tremori, mal di testa, sudorazione profusa) e delle emozioni spiacevoli (come ansia, nervosismo, depressione) se le droghe o l'alcol non sono assunte costantemente. Sono i sintomi astinenziali: effetti fastidiosi o dolorosi che possono rappresentare un ulteriore stimolo al consumo della sostanza nel tentativo di riceverne sollievo.

Commentare con i degenti la tabella con i sintomi astinenziali.

Slide 12

Ricadute nell'uso

Con il progressivo uso la corteccia prefrontale (deputata a modulare ed inibire le emozioni ed i comportamenti impulsivi) viene alterata, facilitando gli automatismi e l'impulsività. Le sostanze, inoltre, possono attivare delle aspettative, le quali determinano l'insorgenza di craving. L'autocontrollo verrà così annullato da una serie di pensieri che giustificheranno e/o minimizzeranno l'idea di assumere la sostanza (ad esempio "Questa è l'ultima", "ogni tanto ci può stare"). Viene inoltre compromessa la capacità di orientare e mantenere l'attenzione, valutare razionalmente i vantaggi e gli svantaggi di una scelta, organizzare pensieri e attività ed, infine, di prendere decisioni.

Slide 13

Far fronte al problema della dipendenza

Le tecniche da utilizzare per affrontare il problema delle dipendenze sono le seguenti: Ripetersi frequentemente quali sono gli svantaggi fisici, sociali e psicologici del bere, Frequentare gruppi di supporto, Chiedere aiuto a qualcuno di cui ci si fida in caso di difficoltà, Rivolgersi ad uno psichiatra e/o psicologo.

Qualcuno di voi ci ha mai provato?

Invitare a fare domande.

Slide 14

Dipendenze comportamentali

Esistono però anche dipendenze sul piano comportamentale.

Sapreste elencarmele?

Sollecitare l'intervento di tutti.

Invitare a fare domande.

Slide 15

Dipendenze comportamentali

Le dipendenze comportamentali sono: gioco d'azzardo, shopping compulsivo, dipendenze digitali, da lavoro, sessuale, da esercizio fisico.

Qualcuno di voi pensa di avere alcune di queste dipendenze? In particolare, per le dipendenze digitali?

Sollecitare l'intervento di tutti.

Invitare a fare domande.

Slide 16

Dipendenze tecnologiche

Le dipendenze digitali stanno diventando sempre più frequenti negli anni. L'uso dei vari dispositivi diventa una dipendenza quando questo altera il funzionamento della persona.

Slide 17

Dipendenze tecnologiche

Secondo voi quando le dipendenze tecnologiche diventano una dipendenza? Fate degli esempi.

Sollecitare l'intervento di tutti.

Invitare a fare domande.

Riassumere quanto detto dai presenti.

Slide 12

Homework

Per la successiva sessione provate ad individuare una situazione specifica e stabilire cosa ti piace e cosa ti spaventa del comportamento di dipendenza.

Prima di lasciarci, volete chiedermi qualcosa? Ci sono cose non chiare? Volete una copia del materiale che ho usato in questo incontro?

Sollecitare l'intervento di tutti

Rispondere ad eventuali domande

Ringraziare i presenti per la loro partecipazione, cercando di mettere in evidenza il contributo di ognuno.

AZIENDA OSPEDALIERO-UNIVERSITARIA
DELLE MARCHE
UNIVERSITÀ POLITECNICA DELLE MARCHE
OSPEDALI RIUNITI di ANCONA
DIPARTIMENTO DI SCIENZE NEUROLOGICHE
CLINICA di PSICHIATRIA

Modulo 5

"Il sonno"

Guida per gli operatori

Il sonno viene definito come uno stato comportamentale reversibile, caratterizzato da isolamento percettivo e assenza di responsività agli stimoli ambientali ma, al tempo stesso, un complesso insieme di processi fisiologici e comportamentali.

In base a misure polisonnografiche, il sonno è stato diviso nelle categorie REM e NREM, o sonno ad onde lente. I cicli del sonno sono detti così per via della loro associazione con la presenza (REM) o assenza (NREM) di rapidi movimenti oculari.

Il riposo, inoltre, influisce sul mantenimento dell'equilibrio psico-emotivo della persona, stabilizzando il tono dell'umore e riducendo i livelli di ansia e stress.

La maggior parte dei ricercatori sostiene che la principale funzione del sonno sia quella di promuovere lo sviluppo cerebrale e l'apprendimento. Durante questa fase, infatti, il cervello elimina le sostanze di scarto attraverso il sistema linfatico, il quale pompa liquor nei tessuti cerebrali, ripulendoli dalle proteine tossiche accumulate durante il giorno.

Numerose sono state, negli ultimi anni, le ricerche relative al sonno, le quali hanno portato alla comprensione di quale sia la sua importanza e quella relativa alla cura dei disturbi legati a questo processo fisiologico.

Tali disturbi sembrano avere un impatto enorme sull'efficienza mentale e, in generale, sulla qualità della vita delle persone.

Gli obiettivi dell'incontro sono i seguenti:

1. conoscere le definizioni e le funzioni del sonno
2. conoscere le regole di igiene del sonno
3. saper monitorare il proprio riposo notturno tramite il diario del sonno

Slide 1
Bentrovati. Oggi dedicheremo questo incontro al tema del sonno.

Slide 2
Il sonno

Il sonno è un processo del quale prenderci cura, in quanto impatta direttamente sulla nostra salute psico-fisica. L'obiettivo dell'incontro di oggi è comprendere le sue funzioni e le regole base per una corretta igiene del sonno.

Iniziamo…

Slide 3
Brainstorming

Condividiamo tutto ciò che ci viene in mente sul concetto di Sonno (favorire gli interventi e appuntarli su una lavagna).

(parole chiave, concetti, musica, colori, esperienze personali…)

Slide 4
Definizioni

Il concetto del sonno è molto difficile da definire in maniera univoca.

Gli studiosi, infatti, ne danno definizioni diverse, in base al focus della sua funzione:

- *è una necessità fisiologica, definito come stato di riposo contrapposto alla veglia*
- *riguarda una periodica sospensione dello stato di coscienza, durante la quale l'organismo recupera energia*
- *stato di riposo fisico e psichico, caratterizzato dal distaccamento temporaneo della coscienza e della volontà, dal rallentamento delle funzioni neurovegetative e dall'interruzione parziale dei rapporti sensomotori del soggetto con l'ambiente.*

Slide 5
Definizioni

Il sonno può sembrare, apparentemente, un momento di "stand-by" che coinvolge corpo e mente. E' stato dimostrato scientificamente che esso, al contrario, è un processo attivo, che coinvolge l'interazione di diverse componenti del sistema nervoso centrale e autonomo.

Slide 6
Video

Ora vediamo insieme un breve video sulla funzione del sonno.

Quale curiosità vi ha colpiti? (favorire gli interventi)

Slide 7
Teorie sul sonno

Secondo questa teoria, il sonno permette di "pulire" le sinapsi (punto di contatto tra i neuroni) da una sorta di "sporcizia" delle informazioni, al fine di migliorare il segnale che passa da un neurone all'altro.

Slide 8
Teorie sul sonno

Un'ulteriore teoria collega il sonno all'apprendimento.

Il sonno permette di consolidare i concetti appresi durante il giorno e di eliminare le informazioni meno utili. Inoltre, permette di trasferire alcune informazioni contenute

nella memoria a breve termine (MBT) nella memoria a lungo termine (MLT).

Slide 9
Teorie sul sonno

Il sonno, attraverso il sistema linfatico, permetterebbe, inoltre, di eliminare alcune sostanze di scarto situate nello spazio tra un neurone e l'altro.

Slide 10
Teorie sul sonno

L'ultima teoria che vogliamo prendere in considerazione oggi è la teoria "sleep to forget, sleep to remember", la quale sostiene che il sonno sarebbe utile, ciclo dopo ciclo, a ridurre l'intensità delle emozioni spiacevoli.

Inoltre, a lungo termine, ci aiuterebbe a ricordare se l'esito di una determinata situazione è stato positivo o negativo, al fine di orientare le nostre scelte future verso quelle più vantaggiose.

Slide 11
Fasi del sonno

L'architettura del sonno presenta fasi cicliche ben definite.

Il sonno è compreso in *5 fasi principali divise in fasi NON REM e fasi REM, coinvolte nel nostro riposo notturno: addormentamento, sonno leggero, sonno profondo, sonno profondo effettivo, sonno REM. Ogni notte concludiamo dai 4 ai 6 cicli ed ognuno di questi può durare dai 90 ai 120 minuti.*

Slide 12

Ora riflettiamo su noi stessi.

Quali benefici ha per voi un sonno di qualità? (favorire gli interventi)

Slide 13
Igiene del sonno

Ora vediamo alcuni accorgimenti che possono aiutarci a mantenere un sonno

efficace e riposante.

Ne conoscete già alcuni? (favorire gli interventi)

Slides 13-25
Igiene del sonno

- *Non passare troppo tempo a letto. Stare a letto per un tempo eccessivamente lungo porta ad avere un sonno superficiale e meno riposante.*
- *Il tempo che trascorri a letto dovrebbe quasi corrispondere alla quantità di tempo in cui in realtà stai dormendo.*
- *Alzati alla stessa ora ogni giorno, sette giorni alla settimana. Questo aiuta ad impostare il tuo orologio biologico e diminuisce la probabilità di insonnia.*
- *L'esercizio quotidiano può aiutare a rendere il sonno più profondo.*
- *Non fare movimento fisico nelle 4 ore che precedono il sonno.*
- *Rendi la tua camera confortevole e priva di rumori e/o luci fastidiose.*
- *Mantieni la temperatura dell'ambiente moderata.*
- *Una stanza troppo calda o troppo fredda può disturbare il tuo sonno.*
- *Consuma pasti regolari e non andare a dormire affamato. Uno spuntino leggero prima di coricarsi può aiutare a dormire. Evita cibi grassi o pesanti.*
- *Cerca di evitare di bere molti liquidi la sera per ridurre al minimo il bisogno di viaggi notturni in bagno.*
- *Evita l'alcool la sera. Il sonno che ottieni quando sei sotto effetto di sostanze alcoliche è frammentato.*
- *Elimina o riduci anche le bevande che contengono caffeina, poiché hanno un effetto stimolante.*
- *Usa la camera da letto solo per il sonno. Non leggere o mangiare a letto.*
- *Non guardare l'orologio; farlo ti renderà solo più frustrato se hai difficoltà a dormire.*
- *Metti l'orologio sotto il letto o giralo in modo da non vederlo.*
- *Evita i sonnellini diurni. Stare svegli durante il giorno ti aiuta a dormire meglio la notte.*
- *Se sei ancora completamente sveglio 15-20 minuti circa dopo esserti messo a letto, alzati per fare qualcosa di diverso come leggere un libro noioso. Non impegnarti in attività stimolanti e mantieni bassi i livelli di luce. Torna a letto solo quando ti senti davvero assonnato.*
- *Quando possibile, elimina l'esposizione alla luce artificiale brillante (luci ambientali, schermi di computer, cellulare) nelle 2 ore prima di andare a dormire. La visione della TV va bene se fatta a distanza di sicurezza dallo*

schermo.

Slide 27
Igiene del sonno (tecnologia)

La luce brillante degli schermi (ad es. smartphone, pc…) stimola l'attività del cervello ed altera il regolare ciclo di sonno-veglia, non consentendo al corpo di andare adeguatamente verso l'addormentamento.

La luce artificiale emanata da cellulare, tablet e PC, ostacola la produzione di melatonina e fa "credere" al cervello che sia ancora giorno, non consentendogli di "staccare la spina".

Slides 28-29
Igiene del sonno

Cerca di non fumare quando hai problemi a dormire, la nicotina ha un effetto uno stimolante

Non portare i tuoi problemi a letto. Prenditi uno spazio per lavorare su situazioni da risolvere o per pianificare le attività del giorno successivo.

Slide 30
Homework

Durante la settimana, ti chiediamo di monitorare il riposo notturno attraverso il diario del sonno, all'interno della quale appunterai:

- *tempo impiegato per addormentarti*
- *a che ora ti sei alzato*
- *il numero di volte in cui ti sei svegliato durante la notte*
- *quanto è stato riposante il tuo sonno (0-10)*
- *cosa può aver disturbato il tuo sonno*

(Consegnare la scheda di lavoro e rispondere ad eventuali richieste)

Intervento Psicoeducativo

Modulo 6

“Le Cure”

Guida per gli operatori

All’interno della guida il *corsivo* viene utilizzato per indicare quello che l’operatore deve pronunciare.

Slide 1

Introduzione all’incontro

Oggi parleremo dei trattamenti che utilizzati nel contesto delle patologie psichiatriche, della loro utilità e dei loro effetti collaterali. Anche su questo argomento, è molto importante che tutti voi e partecipiate attivamente a questo incontro.

Per non fare confusione, cercherò di seguire una scaletta degli argomenti da trattare. Alla fine di questo incontro, se vi sono punti importanti di cui non abbiamo parlato, li vedremo insieme.

Prima di cominciare vorrei chiedervi se avete domande da fare sugli argomenti di cui abbiamo parlato nello scorso incontro.

Volete chiedermi qualcosa prima di cominciare?

Sollecitare l’intervento di tutti.

Rispondere ad eventuali domande o, se non si conosce la risposta, prendere appunti, assicurando i presenti che se ne parlerà la prossima volta.

Slide 2

Obiettivi dell’incontro

Presentare gli obiettivi dell’incontro.

Gli obiettivi dell’incontro saranno:

1. *migliorare la consapevolezza sull’uso dei farmaci, sui benefici e sugli effetti collaterali*
2. *apprendere il modo corretto di prendere i farmaci per ottimizzare i benefici*
3. *individuare strategie per gestire gli effetti collaterali*

Qualcuno (medico di base o psichiatra) vi ha mai spiegato l’importanza della terapia? Qualcuno (medico di base o psichiatra) vi ha mai spiegato come assumere al meglio i farmaci?

Sollecitare l'intervento di tutti.

Slide 3

I tipi di interventi psichiatrici

Le terapie attualmente in uso nel contesto psichiatrico sono le seguenti: Psicoterapia, Terapia farmacologica, ECT, TMS, fototerapia, VNS, stimolazione intracranica, Terapie di tipo «ludico» (es. musicoterapia, art-therapy etc.).

Fornire una breve presentazione e spiegazione delle varie tipologie di terapie, definendo anche le indicazioni in cui vengono utilizzate.

Quante di queste terapie conoscevate? Quali avete utilizzato?

Invitare a fare domande.

Sollecitare l'intervento di tutti.

Slide 4

Psicoterapia e terapia farmacologica

Fornire una breve presentazione e spiegazione delle varie tipologie di terapie, definendo anche le indicazioni in cui vengono utilizzate.

Il trattamento dei disturbi psichiatrici giova particolarmente di un trattamento combinato tra psicoterapia e terapia farmacologica. Non tutti gli approcci psicoterapici sono scientificamente validi. Quelli con maggiori evidenze scientifiche sono rappresentati dal cognitivo-comportamentale, sistemico-relazionale ed interpersonale. Allo stesso tempo la sola psicoterapia non è adatta al trattamento di disturbi psichiatrici quali disturbi psicotici o disturbi bipolare, patologie che hanno necessariamente bisogno di una terapia. La combinazione di entrambi questi interventi terapeutici è sinergica, in quanto lo psicofarmaco può aiutare a stabilizzare il farmaco consentendo allo psicoterapeuta di agire. Allo stesso tempo, la psicoterapia induce un miglioramento della compliance al farmaco e consolida il miglioramento ottenuto farmacologicamente.

Quanti di voi sono stati in carico ad uno psicoterapeuta? Che tipo di orientamento terapeutico aveva?

Sollecitare l'intervento di tutti.

Slide 5

Le tipologie di psicofarmaci

Quali sono gli effetti degli psicofarmaci secondo voi?

Invitare a fare domande.

Sollecitare l'intervento di tutti.

Gli psicofarmaci possono essere classificati in quattro principali categorie: gli antipsicotici, che vanno ad agire sui sintomi psicotici (già trattati nel modulo 3), gli antidepressivi, che migliorano l'umore, gli ansiolitici, il cui ruolo è quello di ridurre la sintomatologia ansiosa e l'agitazione, ed infine i regolatori dell'umore, i quali aiutano a ridurre le oscillazioni del tono dell'umore. È fondamentale però non fossilizzarsi sui nomi, in quanto farmaci di una specifica classe possono avere degli effetti appartenenti ad altre categorie. Ad esempio, la quetiapina a seconda del dosaggio può essere ansiolitico, regolatore dell'umore e antipsicotico.

Role play: A questo punto si invitano i pazienti in modo scherzoso a "costruirsi la terapia", dicendo ad esempio: "Adesso vogliamo proporvi un gioco; ognuno di voi, tenendo conto di quello che c'è scritto sulla slide, si scelga i farmaci che vanno bene per lui". È opportuno che uno dei due conduttori sia in grado di controllare se la terapia scelta corrisponda a quella che il paziente sta facendo. Si elogiano le risposte corrette, dando ad esempio un "voto" alto. Sull'altra lavagna si scrive per ciascun paziente il nome, il trattamento da lui scelto e il voto. Se un paziente decide per un trattamento inadeguato, ci si complimenta con lui per lo sforzo fatto, ma gli verrà assegnato un voto basso, come ad esempio un 6. Si invitano gli altri pazienti ad aiutarlo nel formulare la prescrizione corretta.

Slide 6

Esempi di farmaci psichiatrici

Quali farmaci state assumendo? State assumendo alcuni dei seguenti farmaci?

Invitare a fare domande.

Sollecitare l'intervento di tutti.

Slide 7

Farmacodinamica degli psicofarmaci

Secondo voi come agiscono gli psicofarmaci?

Invitare a fare domande.

Sollecitare l'intervento di tutti.

Gli psicofarmaci agiscono regolando i neurotrasmettitori. I principali neurotrasmettitori coinvolti sono la noradrenalina, che generalmente facilita la razione agli eventi stressanti della vita, la serotonina, presente nelle aree del cervello che regolano i ritmi dell'organismo,

come il sonno e la veglia, la temperatura, l'appetito e i comportamenti sessuali, la dopamina, che ha un ruolo molto importante nel movimento. I neurotrasmettitori hanno un ruolo quindi particolarmente importante, in quanto permettono alle cellule del SNC di comunicare. In realtà, nel nostro organismo le cose sono più complesse di come sembrano, perché questi neurotrasmettitori interagiscono tra di loro e con altre sostanze chimiche e hanno effetto anche in altre zone del nostro organismo. Per questo motivo, ad esempio, gli antidepressivi riducono i sintomi della depressione, ma possono dare anche effetti collaterali, legati alla loro azione su altri sistemi.

Slide 8

Durata del trattamento

Secondo voi per quanto tempo si dovrebbe assumere la terapia?

Invitare a fare domande.

Sollecitare l'intervento di tutti.

Rispondere ad eventuali domande o, se non si conosce la risposta, prendere appunti, assicurando ai presenti che se ne parlerà la prossima volta.

La durata del trattamento farmacologico varia a seconda di numerosi fattori: il tipo di disturbo, la gravità della malattia, la risposta alla terapia ed eventuali trattamenti associati. Il trattamento non deve mai essere sospeso autonomamente ed ogni cambiamento posologico o del tipo di farmaco deve essere concordato con lo psichiatra di riferimento. Ad esempio, nel caso della depressione chi ha avuto un primo episodio depressivo, il rischio di averne un altro è particolarmente alto nei sei mesi successivi. Per questo, dopo un primo episodio, conviene continuare la terapia per almeno sei mesi dalla scomparsa dei sintomi, senza modificare le dosi. Se si smette di prendere gli antidepressivi prima di 6 mesi dalla scomparsa dei sintomi, il rischio di avere una ricaduta nell'anno successivo è tra il 35 e il 60%, mentre se queste medicine sono prese regolarmente per almeno sei mesi, il rischio è più basso, tra il 10 e il 25%. Le persone che, invece, hanno avuto tre o più ricadute devono continuare la cura per almeno 2 anni per evitare ulteriori ricadute.

Slide 9

L'importanza dell'assunzione regolare della terapia

L'assunzione in maniera regolare della terapia permette di mantenere stabili i livelli nel sangue del farmaco. Questo aiuta a ridurre gli effetti collaterali, migliora l'efficacia del farmaco e riduce le ricadute e le ospedalizzazioni. Risulta importante in questo caso l'uso della terapia Long Acting (LAI) che permette di mantenere molto più stabile il livello di

farmaco nel sangue, evitando anche gli effetti di picco della terapia e allo stesso tempo migliorando la compliance e l'aderenza.

A qualcuno è stato proposto un LAI? Lo avete mai assunto?

Slide 10

L'importanza dell'assunzione regolare della terapia

In questo grafico vedete come si modifica nel tempo il livello nel sangue del farmaco con l'assunzione giornaliera della terapia. I livelli tendono ad aumentare fino a raggiungere un certo livello che si manterrà stabile se il farmaco viene assunto in maniera regolare.

Slide 11

Sospensione della terapia

Perché a volte vengono sospesi autonomamente i farmaci?

Invitare a fare domande.

Sollecitare l'intervento di tutti.

Rispondere ad eventuali domande o, se non si conosce la risposta, prendere appunti, assicurando ai presenti che se ne parlerà la prossima volta.

Nell'esperienza comune le risposte tipiche sono:

- perché sto bene
- perché è assurdo prendere per sempre farmaci, fanno male! perché nel prendere farmaci mi sento un malato
- perché me ne dimentico e così poi lascio perdere e non li prendo più
- perché mi danno dei problemi, dei fastidi, degli effetti spiacevoli eccetera.

Il conduttore chiarisce che queste argomentazioni sono tipiche e comuni a tutte le persone che devono assumere farmaci per lungo periodo, indipendentemente dalla natura del di- sturbo, se psichiatrico o malattia fisica. Fornisce quindi esempi di persone che soffrono di ipertensione, di disturbi alla tiroide, di diabete, epilessia, Fa presente che ci sono disturbi per quali le terapie sono cicliche o per determinati periodi.

Chiarisce che tutte i motivi forniti sono così importanti da meritare una maggiore attenzione e, pertanto, le affronta una per una.

Invita, così, gli stessi pazienti a dare validi motivi per "contrastare" le risposte date e le sintetizza nel modo seguente,

- "Perché sto bene". È, invece, il motivo principale per continuare a prenderle

- ....! Gli psicofarmaci sono tra i farmaci più sicuri e maneggevoli se si effettuano i controlli che prescrive il medico.
- ....! Può essere utile discutere di questo aspetto con lo stesso psichiatra o con uno psicologo.
- ....! Ci sono molte strategie per superare le dimenticanze. È importante parlarne con il medico o con un operatore del CSM. Inoltre, alcuni farmaci hanno una formulazione che permette una somministrazione a lunga azione.
- ....! Purtroppo, tutti i farmaci possono avere effetti collaterali. Anche per questo caso è importante parlarne con il medico.

Secondo voi cosa si rischia a sospendere autonomamente la terapia?

Invitare a fare domande.

Sollecitare l'intervento di tutti.

I rischi legati alla sospensione possono essere riassunti nei seguenti: brusco peggioramento del quado clinico, ospedalizzazione, riduzione dell'efficacia del farmaco durante una successiva esposizione dopo la sospensione.

Slide 12

Effetti collaterali della terapia

La terapia farmacologica ha degli effetti collaterali come qualsiasi altro farmaco. L'effetto del farmaco è spesso ritardato rispetto agli effetti collaterali, quindi è possibile che si presentino prima dell'effetto terapeutico. Perciò è fondamentale non sospendere il farmaco quando si presentano nelle fasi iniziale. Possono coinvolgere diversi distretti corporei. I principali sono: Bocca secca, sudorazione eccessiva, sonnolenza, astenia, alterazioni del ritmo cardiaco, insonnia, disturbi sessuali, mal di testa, problemi di vista, nausea, vomito, aumento o diminuzione di peso, stitichezza o diarrea, palpitazioni, pressione bassa, vertigini, irritabilità, tremori.

Avete mai avuto degli effetti collaterali? Cosa avete fatto? Come li avete gestiti?

Invitare a fare domande.

Sollecitare l'intervento di tutti.

Slide 13

Cosa fare quando si sperimentano alcune di queste collateralità?

Quando insorge uno degli effetti collaterali farmacologici la prima cosa da fare è quella di contattare lo psichiatra di riferimento, il quale metterà in atto una delle possibili strategie per ridurre il sintomo. In particolare, la prima cosa che si consiglia è aspettare perché molti

effetti collaterali sono passeggeri e tendono ad attenuarsi da soli nel tempo o a scomparire del tutto. Per questo motivo non conviene interrompere il trattamento se si hanno dei fastidi, ma parlarne con il proprio medico e aspettare qualche giorno prima di decidere cosa fare.

Vi è capitato di avere effetti collaterali che sono scomparsi o si sono attenuati da soli dopo qualche tempo?

Invitare a fare domande.

Sollecitare l'intervento di tutti.

Riassumere quanto detto dai presenti.

Per alcune terapie farmacologiche c'è la possibilità di dosare il livello ematico del sangue. Conseguentemente questo permetterebbe di valutare si i livelli ematici sono troppo elevati, portando così allo sviluppo di alcuni effetti collaterali. Un'altra metodica consisterebbe nel ridurre la dose farmacologica in comune accordo con lo specialista di riferimento. Infatti, La risposta di una persona a un farmaco è individuale, cioè ognuno reagisce un po' diversamente dagli altri. Il medico prescriverà all'inizio una dose che probabilmente avrà bisogno di piccoli aggiustamenti a seconda di come l'organismo reagisce. Per esempio, i disturbi sessuali tendono ad essere dose-dipendenti oltre che a scomparire con la sospensione del farmaco.

Le è mai capitato di avere effetti collaterali che si sono ridotti o sono scomparsi riducendo la dose di antidepressivi?

Invitare a fare domande.

Sollecitare l'intervento di tutti.

Riassumere quanto detto dai presenti.

Alcune persone sono più sensibili a sviluppare un effetto collaterale piuttosto che un altro. Se dopo aver aspettato almeno un mese, o aver ridotto la dose, gli effetti collaterali non si riducono, il medico potrà decidere di sostituire il farmaco con un altro. Nella fase di cambiamento, però, è possibile che la persona abbia più effetti collaterali, quelli propri del farmaco che si sta gradualmente sospendendo e quelli del nuovo.

È mai successo che il medico abbia dovuto cambiare un antidepressivo con un altro perché dava effetti collaterali?

Invitare a fare domande. Sollecitare l'intervento di tutti.

Riassumere quanto detto dai presenti.

Per alcuni effetti collaterali è possibile usare dei rimedi molto semplici. Per esempio, la sonnolenza, che in genere scompare in pochi giorni da sola, se è molto fastidiosa può essere superata prendendo l'antidepressivo la sera, così da sfruttare questo effetto per l'insonnia. Viceversa, se l'antidepressivo dà insonnia, conviene prenderlo al mattino.

Per le vertigini dovute a bassa pressione, spesso basta alzarsi lentamente, mentre per la bocca secca può essere utile mangiare ogni tanto una caramella. La stitichezza è un effetto molto frequente. Il metodo più efficace è quello di fare attenzione alla dieta mangiando più verdura e frutta. Se il problema non si risolve, si può chiedere al medico di prescrivere una tisana lassativa o un altro preparato simile. Anche la diarrea tende ad attenuarsi da sola nel tempo e risponde bene ai fermenti lattici.

A volte per determinati effetti collaterali, il medico prescriverà dei farmaci aggiuntivi. Per esempio, se l'ansia è molto forte, prescriverà degli ansiolitici. Questi farmaci comunque vanno presi per brevi periodi perché può essere difficile riuscire a sospenderli se li si prende per molti mesi di seguito.

Il medico ha mai dovuto prescrivere altri farmaci, a parte gli antidepressivi, per gli effetti collaterali? Quali?

Invitare a fare domande.

Sollecitare l'intervento di tutti.

Riassumere quanto detto dai presenti.

Prima di lasciarci, volete chiedermi qualcosa? Ci sono cose non chiare? Volete una copia del materiale che ho usato in questo incontro?

Sollecitare l'intervento di tutti

Rispondere ad eventuali domande

Ringraziare i presenti per la loro partecipazione, cercando di mettere in evidenza il contributo di ognuno.

Modulo 7

"Abilità comunicative"

Guida per gli operatori

Saper comunicare in maniera efficace rappresenta un'abilità fondamentale per mantenere buone relazioni interpersonali.

Inoltre, comunicare in maniera adeguata aiuta a stare bene con gli altri e rappresenta un elemento fondamentale per mantenere il benessere psicologico.

La comunicazione efficace aiuta a gestire gli eventi stressanti e favorisce una rapida risoluzione dei problemi della vita quotidiana.

Nel presente modulo vengono affrontate in particolare le abilità che permettono di migliorare il rapporto con gli altri: l'abilità di esprimere sentimenti piacevoli, l'abilità di fare richieste in maniera positiva, l'abilità di esprimere sentimenti spiacevoli e la capacità di ascolto attivo.

Gli obiettivi dell'incontro sono i seguenti:
1. apprendere modalità di comunicazione efficace
2. saperle mettere in pratica nella vita quotidiana

Il conduttore saluta i presenti e li invita a parlare dei progressi fatti e delle difficoltà incontrate nel raggiungimento degli obiettivi personali e prende informazioni sull'andamento degli incontri psicoeducativi.

Slide 1
Abilità comunicative

Come abbiamo già discusso negli incontri precedenti, possiamo trovarci ad affrontare diversi problemi nella vita di tutti i giorni.

Slide 2
Abilità comunicative

Tali problemi possono essere sia di tipo pratico che di natura interpersonale come, ad esempio, incomprensioni che si possono creare tra due persone.
Avere un problema con un'altra persona, specialmente se è una persona cara, genera uno stato di tensione che contribuisce allo stress.
Per migliorare il nostro benessere, dunque, è importante apprendere come comunicare in maniera efficace.

Slide 3
Abilità comunicative

Viene spiegato ai partecipanti che le relazioni interpersonali sono influenzate positivamente o negativamente dalla capacità di comunicare e che il vantaggio del miglioramento di tale abilità crea una maggiore sensazione di benessere.

Slide 4
Emozioni piacevoli
Iniziamo ad approfondire, nel dettaglio, le abilità di cui tratteremo oggi.

Perchè secondo voi è importante esprimere sentimenti piacevoli? (favorire gli interventi)

Slide 5
Emozioni piacevoli

Nella vita di tutti i giorni c'è sempre qualcuno che ha fatto qualcosa di buono e positivo per l'altro, anche se spesso non gli diamo troppa importanza.

Slide 6
Role-play (emozioni piacevoli)

Vi invito a pensare ad un episodio da portare come esempio.
(Il conduttore favorisce il role-play con due interlocutori)

Slide 7
Comunicare emozioni piacevoli

Come dire ad una persona che ci è piaciuto il suo modo di fare?

- *Guardala negli occhi*
- *Assumi una mimica e una postura adeguata al sentimento provato*
- *Dille cosa ha fatto che ti è piaciuto*
- *Dille come ti sei sentito (comunicare l'emozione, il sentimento, la sensazione)*

Slide 8
Homework

Il conduttore invita a mettere in pratica questa abilità nei giorni successivi consegnando l'apposita scheda per annotare i comportamenti degli altri che ci hanno suscitato emozioni piacevoli

(consegnare scheda di lavoro e raccogliere eventuali richieste)

Slide 9
Emozioni spiacevoli

Ora ci occuperemo della prossima abilità, che ci permetterà di apprendere come comunicare in maniera efficace le emozioni spiacevoli.

Perché è importante secondo voi saper esprimere emozioni spiacevoli? (favorire gli interventi)

Slide 10
Emozioni spiacevoli

Saper esprimere in maniera efficace i sentimenti spiacevoli, favorisce nel futuro che il comportamento dell'altro che ci ha ferito non si ripeta e dimostra che siamo disponibili e predisposti a chiarire la situazione.

Così facendo, dimostriamo che teniamo a lui/lei.

Slide 11
Emozioni spiacevoli

Nella vita di tutti i giorni possono capitare episodi in cui possiamo sentirci amareggiati, irritati, delusi dal comportamento di qualcun altro.

Alcuni, per esempio, tendono a rimuginare e a non parlare con il diretto

interessato dei sentimenti spiacevoli e questo genera spesso un crescente risentimento verso quella persona.

Slide 12
Role-play (emozioni spiacevoli)

Vi invito a pensare ad un episodio da portare come esempio.
(Il conduttore favorisce il role-play con due interlocutori)

Slide 13
Comunicare emozioni spiacevoli

Come dire ad una persona che non ci è piaciuto il suo modo di fare?
- *Guardala negli occhi*
- *Assumi una mimica e una postura adeguata al sentimento provato*
- *Dille cosa ha fatto che non ti è piaciuto*
- *Dille come ti sei sentito (comunicare l'emozione, il sentimento, la sensazione)*
- Proponi una soluzione per evitare che la situazione accada in futuro;
- Suggerisci anche come poter modificare quel comportamento

Slide 14
Homework

Il conduttore invita a mettere in pratica questa abilità nei giorni successivi consegnando l'apposita scheda per annotare i comportamenti degli altri che hanno suscitato emozioni spiacevoli.

(consegnare scheda di lavoro e raccogliere eventuali richieste)

Slide 15
Richieste

L'abilità successiva riguarda come porre richieste in maniera positiva agli altri.

Perché è importante secondo voi saper fare richieste in maniera positiva? (favorire gli interventi)

Slide 16
Richieste

Può capitare che quando chiediamo un favore o facciamo una richiesta a un amico o un familiare, questa venga interpretata come un ordine, oppure non venga compresa come reale richiesta perchè fatta in maniera indiretta.

Pertanto, migliorare questa abilità permette anche di evitare incomprensioni o fraintendimenti.

Slide 17
Role-play (richieste)

Vi invito a pensare ad un episodio da portare come esempio.

(Il conduttore favorisce il role-play con due interlocutori)

Slide 18
Richieste

Come fare richieste in maniera positiva?
- *Guarda la persona negli occhi*
- *assumi una mimica e una postura adeguata*
- *digli in modo preciso cosa vorresti facesse per te*
- *digli cosa proveresti e come ti sentiresti se facesse quanto richiesto*

Slide 19
Homework

Il conduttore invita a mettere in pratica questa abilità nei giorni successivi consegnando l'apposita scheda per annotare le situazioni in cui si è messa in atto una richiesta positiva.

(consegnare scheda di lavoro e raccogliere eventuali richieste)

Slide 20
Ascolto attivo

Ora ci occuperemo dell'ultima abilità comunicativa di questo modulo: l'ascolto attivo.

Perché, secondo voi, è importante saper ascoltare l'altro in modo attivo?

Slide 21
Ascolto attivo

Saper ascoltare in modo attivo, cioè con attenzione e partecipazione, migliora la relazione tra le persone e fa capire che siamo interessati a lui/lei.

Slide 22
Role-play (ascolto attivo)

Scegliete un breve episodio da riportare al vostro compagno poi invertitevi, cercando nel ruolo dell'ascoltatore, di mettere in atto l'ascolto attivo.

Slide 23
Ascolto attivo

Come saper ascoltare l'altro in maniera attiva?
- *Guardalo negli occhi*
- *assumi una mimica facciale ed una postura adeguate (es. Fare cenni con il capo)*
- *fagli capire che sei attento a ciò che dice*
- *fai domande di chiarimento*
- *sintetizza quanto detto con le parole dell'altro*

Slide 24
Homework

Il conduttore invita a mettere in pratica questa abilità nei giorni successivi consegnando l'apposita scheda per annotare le situazioni nelle quali si è messo in atto l'ascolto attivo.

(consegnare scheda di lavoro e raccogliere eventuali richieste finali)

Intervento Psicoeducativo

Modulo 8

"Problem Solving"

Guida per gli operatori

All'interno della guida il *corsivo* viene utilizzato per indicare quello che l'operatore deve pronunciare.

Slide 1

Introduzione all'incontro

Oggi parleremo delle tecniche di problem solving, le quali vi aiuteranno ogni volta che dovete prendere una decisione. Successivamente proveremo a metterle in pratica attraverso degli scenari fittizi. Anche su questo argomento, è molto importante che tutti voi e partecipiate attivamente a questo incontro.

Per non fare confusione, cercherò di seguire una scaletta degli argomenti da trattare. Alla fine di questo incontro, se vi sono punti importanti di cui non abbiamo parlato, li vedremo insieme.

Prima di cominciare vorrei chiedervi se avete domande da fare sugli argomenti di cui abbiamo parlato nello scorso incontro.

Volete chiedermi qualcosa prima di cominciare?

Sollecitare l'intervento di tutti.

Rispondere ad eventuali domande o, se non si conosce la risposta, prendere appunti, assicurando i presenti che se ne parlerà la prossima volta.

Slide 2

Obiettivi dell'incontro

L'unico obiettivo dell'incontro di oggi è quello di imparare la tecnica del problem solving, vi aiuterà a risolvere più facilmente i problemi di tutti i giorni o a raggiungere i vostri obiettivi.

Avete mai sentito parlare di questa tecnica? L'avete mai utilizzata?

Sollecitare l'intervento di tutti.

Invitare a fare domande.

Slide 3

Come risolvere un problema

La tecnica del problem solving si compone delle seguenti tappe:

- *Precisare qual è il problema o l'obiettivo, scegliere inizialmente degli obiettivi semplici e poi renderli sempre più complessi.*
- *Elencare più soluzioni senza commentarle, bisogna sospendere il giudizio e includere nella lista anche le soluzioni più bizzarre o strane.*
- *Discutere brevemente i vantaggi e gli svantaggi di ogni soluzione.*
- *Scegliere la soluzione "migliore", sia in termini di risorse impiegabili che di attuabilità.*
- *Fare un piano per metterla in pratica.*
- *Verificare se il piano funziona.*

Invitare a fare domande.

Sollecitare l'intervento di tutti.

Riassumere quanto detto dai presenti.

Slide 4

Prova pratica

Ora proveremo a mettere in pratica questa tecnica usando degli scenari fittizi.

Scegliere gli scenari più adatti alla tipologia di pazienti:

- Un compagno ti disturba mentre tu vuoi stare attento.
- Un compagno ti prende in giro in modo antipatico.
- Quando vai a casa della tua amica, la trovi in compagnia di un'altra ragazza e non ti fanno partecipare ai loro discorsi. Tu ti senti esclusa da loro.
- Una bambina vuole che il fratellino smetta di romperle le bambole o altri oggetti personali.
- Tua mamma o tuo papà danno spesso ragione al tuo fratellino più piccolo, anche quando lui ha torto, solo perché "Tu sei più grande, devi capire".
- La tua migliore amica si è appena messa con un ragazzo e tu non riesci più a vederla spesso come prima perché lei è molto presa da lui.
- I tuoi genitori pretendono che tu prenda sempre voti molto alti, altrimenti ti rimproverano o ti mettono in punizione.

- A scuola ti prendono in giro perché sei un po' grasso. Non vorresti sembrare così diverso da loro.
- Sullo scuolabus qualcuno si comporta spesso in modo prepotente con i più piccoli.
- Hai confidato un segreto a qualcuno di cui avevi fiducia, ma questa persona l'ha riferito a qualcun altro.
- Un tuo amico ti chiede di fare una cosa che tu non vuoi fare. Non hai il coraggio di dire di no, perché lui minaccia di non essere più amico tuo.
- Un insegnante ti accusa di aver fatto una cosa grave, come aver rubato o rotto qualcosa a scuola. Non sei stato tu ma sai che è stato un tuo compagno.
- Un insegnante ti mette in punizione per qualcosa che non hai fatto. Hai Cosa potresti dire o fare? l'impressione che ce l'abbia con te. Cosa potresti dire o fare?
- Un tuo compagno apre la sua cartella e tu noti che contiene diversi oggetti che i tuoi amici credevano di avere perso. Cosa potresti dire o fare? Nei confronti dei tuoi amici? Nei confronti di questo compagno?
- Un insegnante ti mette in punizione per qualcosa che non hai fatto. Il giorno seguente ti chiede di aspettarlo dopo la fine delle lezioni. Cosa potresti dire o fare?
- Hai appena iniziato ad andare in una nuova scuola. Alcuni compagni ti prendono in giro e ti affibbiano nomignoli imbarazzanti. Cosa potresti dire o fare?
- Il tuo fratellino o la tua sorellina ti confida di essere vittima di suoi compagni/e bulli/e. Cosa potresti dire o fare?
- Un tuo compagno ti riferisce che qualcuno a scuola sta diffondendo bugie sul tuo conto. Cosa potresti dire o fare, nei confronti di questo compagno? E nei confronti delle persone che starebbero dicendo bugie su di te?
- I tuoi genitori sono partiti per il week-end lasciandoti a badare alla casa. I tuoi amici lo vengono a sapere e ti chiedono di organizzare una festa a casa tua. Tuo fratello più piccolo minaccia di riferire tutto ai tuoi genitori, in caso succeda qualcosa. Cosa potresti fare?
- Stai tornando a casa dopo una festa. È mezzanotte passata, sta piovendo e sei a 10 chilometri da casa. Sei preoccupato di fare tardi. Sei con 3 amici e uno di loro suggerisce di rubare un'auto. Cosa potresti fare?
- Un tuo amico ha 2 biglietti per un concerto. Tu gli chiedi di darne uno a te, anche se era stato acquistato per un altro amico. Incontri la persona a cui dovrebbe andare il biglietto. Cosa potresti fare?
- Senti un tuo compagno raccontare che la tua ragazza è stata vista con un tuo amico (o il tuo ragazzo è stato visto con una tua amica) a una festa. Cosa potresti fare?
- Tua madre ti chiede di badare alla tua sorellina sabato sera, ma tu vuoi uscire. Cosa potresti fare?

- Qualcuno sta diffondendo notizie false su di te. Cosa potresti fare? Hai prestato il tuo CD preferito a un amico, che ti dice di averlo perso. Cosa potresti fare?
- Un amico ti riferisce che piaci a una ragazza (o a un ragazzo). Incontri per strada questa persona a cui ti dicono che piaci. Cosa potresti fare?

Slide 5

Homework

Per la successiva sessione provate ad usare la tecnica del problem solving e riportatela nella scheda allegata.

Prima di lasciarci, volete chiedermi qualcosa? Ci sono cose non chiare? Volete una copia del materiale che ho usato in questo incontro?

Sollecitare l'intervento di tutti

Rispondere ad eventuali domande

Ringraziare i presenti per la loro partecipazione, cercando di mettere in evidenza il contributo di ognuno.

Intervento Psicoeducativo

Modulo - "Social Skill"

Guida per gli operatori

All'interno della guida il *corsivo* viene utilizzato per indicare quello che l'operatore deve pronunciare.

Slide 1

Introduzione all'incontro

Oggi proveremo a sviluppare le vostre abilità sociali, queste infatti possono essere alterate quando una persona soffre di depressione e ansia. È molto importante che tutti voi e partecipiate attivamente a questo incontro.

Per non fare confusione, cercherò di seguire una scaletta degli argomenti da trattare. Alla fine di questo incontro, se vi sono punti importanti di cui non abbiamo parlato, li vedremo insieme.

Prima di cominciare vorrei chiedervi se avete domande da fare sugli argomenti di cui abbiamo parlato nello scorso incontro.

Volete chiedermi qualcosa prima di cominciare?

Sollecitare l'intervento di tutti.

Rispondere ad eventuali domande o, se non si conosce la risposta, prendere appunti, assicurando i presenti che se ne parlerà la prossima volta.

Il training è condotto in un gruppo formato dal condutture **in base alle caratteristiche dei singoli membri e alle finalità dell'intervento** (ad esempio in base alle specifiche abilità nelle quali i soggetti sono carenti).

Deve essere sviluppato seguendo un modello di otto passi.

1. Presentazione e definizione del problema

Il conduttore innanzitutto presenta le principali situazioni problematiche. per poi aiutare il gruppo a definire l'effettivo problema. In seguito, guida il gruppo a generare diverse ipotesi di soluzioni.

2. Individuazione delle soluzioni

Dopo che il gruppo ha definito il problema e ha trovato alcune soluzioni alternative appropriate, il conduttore aiuta i membri a individuare le componenti delle varie soluzioni

costituite da abilità sociali. Una volta individuata la soluzione migliore, il conduttore fornisce adeguate istruzioni al gruppo su come mettere in atto il comportamento sociale desiderato.

3. Modelling

Prima che i membri del gruppo mettano in atto personalmente le nuove abilità, il conduttore deve modellarle correttamente. Trattandosi di un training a base cognitivo-comportamentale, il modelling deve includere le due componenti: quella cognitiva e verbale, nella quale il conduttore pensa a voce alta i passi della messa in atto dell'abilità; e quella comportamentale, in cui dimostra la vera e propria esecuzione della stessa. Grazie al modelling attuabile sulle due componenti, i ragazzi possono osservare non solo il corretto processo di autoistruzioni, ma anche la corretta esecuzione del comportamento relativo alla nuova abilità.

4. Ripetizione del comportamento e role-playing

Una volta che il conduttore ha mostrato le autoistruzioni verbali dell'abilità sociale desiderata e un esempio comportamentale, ciascun membro del gruppo esegue la stessa prova. È importante che il ragazzo sia sollecitato a mettere in atto il comportamento desiderato per mezzo di role-playing realistici e per lui rilevanti.

5. Feedback sulla performance

Dopo che il soggetto ha ripetuto il comportamento e ha eseguito il role-playing dei comportamenti desiderati, gli va subito dato un feedback sulla sua performance. Se la prova è stata adeguata, lo si deve rinforzare (lodandolo), mentre se non lo è stata è necessario dargli indicazioni su come migliorare l'esecuzione e fornire un ulteriore esempio pratico. Il soggetto deve ripetere il comportamento fino a che non lo esegue correttamente. Ogni feedback dato sul comportamento del soggetto deve descrivere concretamente ciò che va bene e ciò che deve essere corretto, così che egli possa comprendere esattamente cosa fare.

6. Gestione dei comportamenti problematici

Non sempre questo passo è necessario. Lo diventa quando i membri del gruppo, durante l'incontro, si comportano in modi inadeguati: fanno qualcosa che esula dall'attività in corso, non obbediscono, hanno comportamenti antisociali e così via. Sembra essere molto efficace in tal senso l'applicazione di un piano comportamentale basato sul rinforzo, come ad esempio un sistema di token economy. Può anche essere indicato dedicare un po' di tempo del primo incontro a stendere una serie di regole di comportamento, che verranno esposte in modo visibile nelle sessioni successive. Se si rivelassero necessarie, perciò, andrebbero usate procedure di rinforzo e correttive, per ridurre i comportamenti inadeguati e insegnare le regole di gruppo.

7. Autoistruzioni e autovalutazione

Ai partecipanti viene espressamente richiesto di "pensare ad alta voce" durante gli incontri del training, così come ha fatto per primo il conduttore nella fase di modelling. Ciò serve anche a individuare eventuali autoaffermazioni distorte o sistemi di credenze disfunzionali.

Con il procedere del training, le istruzioni esplicite e le valutazioni espresse dal conduttore andrebbero gradualmente trasformate in autoistruzioni e in autovalutazioni. In ultima analisi, i partecipanti dovrebbero sviluppare le capacità di gestirsi da soli nelle situazioni problematiche e di darsi un feedback autonomamente.

8. Training per la generalizzazione e il mantenimento delle abilità apprese

Questo ultimo passo è fondamentale per la completa riuscita dell'intervento. In caso contrario, gli eventuali progressi dovuti al training resterebbero confinati alla stanza in cui il gruppo ha lavorato.

Per favorire la generalizzazione degli apprendimenti è bene che le situazioni, i comportamenti e gli esercizi di role-playing scelti nel corso dell'intervento siano il più possibile realistici e comunque simili alle situazioni sociali che i partecipanti vivono nel loro ambiente naturale: a scuola, a casa e negli altri contesti.

Anche se tutti questi accorgimenti non costituiscono una garanzia assoluta che i progressi ottenuti saranno generalizzati ad altri contesti o mantenuti nel tempo, è certo che senza di essi sarà molto difficile che ciò avvenga.

Esempio di applicazione degli otto passi fondamentali del training di abilità sociali

Si riporta qui di seguito, a mo' di esempio, l'applicazione degli otto pasi del training di abilità sociali condotta con un gruppo di ragazzi con ansia sociale. L'abilità affrontata nella seduta riguarda come iniziare una conversazione con qualcuno.

1. Presentazione e definizione del problema

«Oggi impareremo le abilità necessarie per iniziare una conversazione con un'altra persona. Cos'è una conversazione?» Avviare un discussione di gruppo. Possibili risposte: 1. parlare con un'altra persona di cose che interessano a entrambi; 2. comunicare con un'altra persona, sia parlando che ascoltando. Vi è mai capitato di notare che qualcuno avesse iniziato una conversione in un modo sbagliato? Cosa è successo in quel caso?» Avviare una discussione sui modi sbagliati di iniziare una conversazione. «Perché è importante sapere come iniziare una conversazione in modo adeguato?»

Aiutare i partecipanti a trovare alcune risposte. Possibili risposte: 1. così si può fare amicizia; 2. così si possono avere informazioni dagli altri; 3. così puoi far sapere ad altri qualcosa che per te è importante.

2. Individuazione delle soluzioni

«Ecco come iniziare una conversazione:

1. cercate un buon momento per iniziare (ad esempio non interrompete qualcuno che sta parlando);

2. salutare l'altro ("Ciao", "Buongiorno", "Salve");

3. guardate l'altro negli occhi;

4. verificate che l'altro vi stia prestando attenzione (vi sta guardando?);

5. ditegli ciò che intendevate dirgli.

Bene, a questo punto rivediamo insieme i punti di cui ho parlato finora, vorrei capire se vi sono chiari: cosa è necessario che voi facciate per dare il via a una conversazione?»

Aiutare tutti i partecipanti a individuare i vari punti e assicurarsi che nessuno resti escluso da questa verifica.

3. Modelling

«Osservate come io inizio una conversazione con un'altra persona secondo punti che abbiamo appena rivisto."

Fare un esempio che ripercorra i 5 punti con un co-terapeuta o con uno dei partecipanti che recita il ruolo dell'interlocutore, mettendo in atto sia il comportamento sia la verbalizzazione dei propri pensieri (pensare ad alta voce).

4. Ripetizione del comportamento e role-playing

«Ora voglio che ciascuno di voi faccia pratica di queste abilità. Prima ditutto, ripassiamo ancora una volta i 5 punti.»

Si ripassano brevemente i 5 punti con l'aiuto di un tabellone.

«Adesso è il momento di provare.»

Ogni partecipante, a turno, seguirà la scaletta suggerita, prendendo come interlocutore uno dei compagni (ciascuno avrà la possibilità di interpretare a turno i due ruoli). Se necessario, il conduttore darà suggerimenti per aiutare ogni soggetto a compiere bene la sua esercitazione. Aiutare i partecipanti a scegliere situazioni realistiche per il role-playing; l'interlocutore verrà scelto anche in base alla sua somiglianza, per alcuni aspetti, con persone che realmente si incontrano nelle situazioni quotidiane.

5. Feedback sulla performance

Se la simulazione è stata eseguita correttamente, rinforzare il soggetto, specificando concretamente ciò che ha eseguito bene: «Bella prova!» o «Molto bene!». Se i passi non sono

stati eseguiti tutti in modo corretto, dare un feedback correttivo: «È stato un buon tentativo, ma occorre migliorare alcune cose». Descrivere i punti che il soggetto ha eseguito bene e quelli che deve fare meglio. Il conduttore ripeterà la simulazione, dando di nuovo un esempio di come vanno eseguiti i punti non eseguiti correttamente. Il partecipante ripeterà la sequenza fino a che non la farà correttamente.

6. Gestione dei comportamenti problematici

Non è stato necessario perché non si sono presentati comportamenti problematici.

7. Autoistruzioni e autovalutazione

«Ora, vediamo come imparare a gestire da soli questi 5 punti.»

Modellare ad alta voce le autoistruzioni e i suggerimenti utili a eseguire ciascun punto. I partecipanti faranno la stessa cosa a turno. Come possiamo sapere se abbiamo iniziato la conversazione nel modo giusto?" Fornire indicazioni per allenarsi all'autovalutazione: «Sono stati eseguiti tutti i 5 punti? La conversazione è andata bene? Ho avuto un buon risultato?».

8. Training per la generalizzazione e il mantenimento delle attività apprese

«Avete fatto un buon lavoro su come iniziare una conversazione. Ciascuno di voi deve esercitare queste abilità nei prossimi giorni e quando ci rivedremo racconterete al gruppo come siete andati. In quali situazioni potreste allenarvi?»

Aiutare i partecipanti a formulare alcune idee di situazioni pratiche in cui esercitarsi a iniziare una conversazione. Si daranno precise disposizioni a ciascun membro del gruppo su ciò che dovrà impegnarsi a fare nei giorni successivi e di cui dovrà riferire nell'incontro seguente.

Slide 2

Obiettivi dell'incontro

Gli obiettivi dell'incontro di oggi sono di incrementare le abilità sociali di ciascuno, prendendo in considerazione diversi aspetti della socialità.

Slide 3

Iniziare una conversazione con uno sconosciuto

Sono molte le situazioni in cui si desidera iniziare una conversazione con un'altra persona. Può trattarsi di una persona che non conoscete bene o di una persona che non avete mai incontrato ma che vorreste conoscere. A volte le persone si sentono timide nell'iniziare una conversazione. Ci accorgiamo che le cose vanno meglio quando si tengono a mente alcuni passaggi specifici:

- *Scegliete il momento e il luogo giusto.*

- *Se non conoscete la persona, presentatevi. Se si conosce la persona, dite "Ciao".*
- *Scegliete un argomento di cui volete parlare o fate una domanda.*
- *Giudicate se l'interlocutore vi ascolta e vuole parlare.*

Esempi di role-play da usare:

- Una nuova persona inizia a frequentare il centro diurno.
- Le persone stanno aspettando l'inizio di un'attività nella comunità residenziale o nel centro diurno.
- Siete a una riunione di famiglia.
- Si è seduti con un'altra persona a pranzo.
- State incontrando per la prima volta il vostro nuovo case manager.

Slide 4

Continuare le conversazioni

A volte si vuole andare oltre una breve conversazione; si desidera parlare più a lungo con una persona perché questa ci piace o perché siamo interessati a ciò che è stato detto. Spesso le persone non sanno come mantenere una conversazione o si sentono a disagio. Un modo per continuare a parlare è fare domande. Un altro modo è quello di fornire informazioni concrete all'altra persona. Questo permette alle persone di conoscere meglio l'altro e le cose che potrebbero avere in comune. Le informazioni concrete sono quelle che dicono chi, cosa, dove, quando e come. Un ulteriore modo è quello di dire a qualcuno come ci si sente. Questo permette alle persone di conoscere meglio i sentimenti dell'altro e di capire se hanno più cose in comune di cui parlare. Esempi di sentimenti che possono essere espressi sono: felice, triste, eccitato, deluso, contento, turbato e irritato. Seguite i seguenti passaggi:

- *Salutate la persona.*
- *Usate le seguenti tecniche per continuare la conversazione:*
    - *Fate una domanda su qualcosa che vorreste sapere.*
    - *Condividete alcune informazioni su un argomento che vorresti discutere.*
    - *Fate una breve descrizione di come vi fa sentire qualcosa.*
- *Valutare se la persona sta ascoltando ed è interessata a proseguire la conversazione.*

Esempi di role-play da usare:

- Guardate un programma televisivo con un'altra persona che sembra apprezzare il programma.
- Vedete il proprio compagno di stanza dopo che ha trascorso una giornata in famiglia.
- Prendete un caffè con un amico del centro diurno.
- Condividete un compito (come pulire dopo cena) con qualcuno.

- Parlate con un consulente di un programma di inserimento lavorativo assistito.
- Raccontate a un amico di un film o di un programma televisivo visto di recente.
- Raccontate a un consulente o a un membro del personale ciò che si è discusso in gruppo.
- Raccontate a qualcuno un articolo letto sul giornale.
- Raccontate a un membro del personale un piatto che vi è piaciuto al ristorante.
- Raccontate a un membro dello staff che non vi piacciono i compiti assegnati nella residenza comunitaria.
- Dite al vostro case manager che vi è piaciuto l'ultimo gruppo.
- Dite a un familiare che siete entusiasti di andare al cinema questo fine settimana.
- Dite a un membro del personale che si è delusi per l'annullamento di una festa del centro diurno.

Slide 5

Concludere una conversazione

Le conversazioni non vanno avanti per sempre, prima o poi qualcuno dovrà terminarla. Molte volte può capitare che spetti proprio a voi concluderla. Ci sono molte ragioni per terminare una conversazione, tra cui la mancanza di tempo, la necessità di andare da qualche altra parte o la mancanza di cose da dire. È possibile terminare le conversazioni in modo più agevole se si tengono a mente alcuni passi:

- *Aspettate che l'interlocutore abbia finito di parlare.*
- *Utilizzate un segnale non verbale, come uno sguardo distratto o un'occhiata all'orologio.*
- *Fate un commento conclusivo come "Beh, ora devo proprio andare".*
- *Dite: "Arrivederci".*

Esempi di role-play da usare:

- Parlate di un programma televisivo con qualcuno in comunità, ma arriva l'ora del gruppo serale.
- State finendo di pranzare con un'altra persona al centro diurno, ma arriva l’ora di incontrare il proprio consulente.
- Parlate con un amico prima dell'inizio del gruppo.
- Parlate con una nuova persona al centro di accoglienza e non avete più niente da dire.
- Parlate con un amico durante la colazione, ma è ora di andare al lavoro.

Slide 6

Partecipare ad una conversazione già in corso

Ci sono momenti in cui tutti vogliono partecipare ad una conversazione in corso. Può trattarsi di argomenti interessanti, del desiderio di condividere un'opinione o forse solo del bisogno di far parte di un gruppo. Molte persone hanno difficoltà a sapere esattamente come farlo in modo da non interrompere il "flusso" della conversazione. I seguenti passi sono utili per entrare in una conversazione in corso:

- *Aspettate una pausa nel flusso della conversazione.*
- *Dite qualcosa come "Posso unirmi a voi?".*
- *Decidete se le persone impegnate nella conversazione sono d'accordo che vi uniate a loro.*
- *Dite cose attinenti all'argomento della conversazione.*

Esempi di role-play da usare:

- Siete a una festa e siete interessati a partecipare ad una conversazione in corso.
- Siete al vostro centro diurno e sentite diverse persone che parlano della partita di baseball di ieri sera. Anche voi avete visto la partita e volete partecipare alla conversazione.
- Durante il colloquio sul caso clinico sembra che tutti, tranne voi, stiano discutendo dei vostri obiettivi. Volete condividere le vostre idee su nuovi obiettivi.
- Siete a cena con i vostri familiari e stanno discutendo di diverse opzioni per le vacanze estive. Avete alcune idee e vorreste condividerle.
- Gli amici stanno decidendo quale film vedere e voi volete dare un suggerimento.

Slide 7

Rimanere sull'argomento stabilito da un'altra persona

Quando si è in una conversazione con un'altra persona, è importante dimostrare di prestare attenzione a ciò che viene detto. Riuscire a rimanere concentrati sull'argomento in discussione dimostra all'interlocutore che si sta ascoltando e che si è interessati a ciò che viene detto. I seguenti passi sono utili per rimanere sull'argomento di discussione in corso:

- *Decidere quale sia l'argomento ascoltando la persona che sta parlando.*
- *Se dopo l'ascolto non si capisce quale sia l'argomento, chiedere all'interlocutore.*
- *Dite cose correlate all'argomento.*

Esempi di role-play da usare:

- Un membro del personale della residenza comunitaria vi parla della nuova lista delle faccende da sbrigare.
- Un consulente del centro diurno vi parla di un nuovo gruppo avviato.
- Un amico vi parla di un film che ha visto.

- Il vostro compagno di stanza vi spiega come dipingere la vostra stanza di un nuovo colore.
- Il vostro medico vi propone di mangiare cibi sani.

Slide 8

Cosa fare quando una persona va fuori argomento

Per sostenere una conversazione è necessario che entrambe le persone capiscano l'argomento, permettendo a ciascuno di contribuire con il proprio pensiero e rendendola più significativa per entrambi. A volte, però, ci si ci troviamo in una situazione in cui l'altra persona si è improvvisamente allontanata dall'argomento discussione, lasciandoci confusi. Quando ciò accade, è meglio far capire immediatamente all'interlocutore che siamo confusi e poi cercare di tornare all'argomento originale:

- *Dite qualcosa del tipo: "È interessante; possiamo parlarne dopo aver finito questa discussione?".*
- *Se la persona ha dimenticato qual è l'argomento, ricordateglielo gentilmente.*
- *Valutate se l'interlocutore è ancora interessato all'argomento iniziale.*
- *Se l'interlocutore è interessato, continuate la discussione. Se non è interessato, chiudete gentilmente la conversazione o parlate di qualcosa di nuovo.*

Esempi di role-play da usare:

- Siete nel bel mezzo di una discussione con un amico su un film che avete visto entrambi, quando il vostro amico inizia improvvisamente a parlare del tempo.
- State parlando a vostra madre del nuovo lavoro che avete appena iniziato, quando inizia a parlarvi di vostro cugino che si è appena arruolato nell'esercito.
- Il vostro case manager sta discutendo con voi dei progressi fatti nel programma di inserimento lavorativo assistito, quando viene interrotto da una telefonata. Dopo la telefonata, riprende la conversazione con voi e inizia a parlare di quando potrete andare a comprare un cappotto.
- Il vostro coinquilino vi chiede indicazioni per il museo. Mentre iniziate a fornirgli le indicazioni, lui cambia improvvisamente argomento, passando allo shopping.
- State pranzando con un'amica che vi sta parlando di un programma televisivo che ha visto ieri sera, quando, nel bel mezzo della sua descrizione, inizia a parlarvi di una nuova persona che ha appena conosciuto.

Slide 9

Formulare una lamentela

Molte situazioni spiacevoli possono essere evitate esprimendosi chiaramente e facendo richieste in modo positivo. Tuttavia, spesso si verificano situazioni in cui accade qualcosa di spiacevole. In questi casi è necessario presentare un reclamo. Una lamentela di solito funziona meglio se si può anche suggerire una soluzione:

- *Guardate la persona.*
- *Parlate con fermezza e calma.*
- *Esprimete il vostro reclamo. Siate specifici in merito alla situazione.*
- *Dite all'interlocutore come si potrebbe risolvere il problema.*

Esempi di role-play da usare:

- Si perdono soldi in un distributore automatico.
- Qualcuno vi interrompe mentre state parlando.
- Ordinate un cheeseburger, ma la cameriera vi porta un hamburger semplice.
- Comprate un abbonamento dell'autobus e l'impiegato vi dà il resto sbagliato.
- Qualcuno in un'area non fumatori si accende una sigaretta.

Slide 10

Rispondere ad una lamentela

Per quanto possiate cercare di essere attenti e premurosi, ci saranno momenti in cui qualcuno dovrà presentare una lamentela nei vostri confronti. Ad esempio, si urta per sbaglio qualcuno o si dimentica un appuntamento. Se vi arrabbiate quando qualcuno si lamenta con voi, non farete altro che peggiorare la situazione. Seguendo le indicazioni che seguono si potrà affrontare la situazione in modo pacato:

- *Guardate la persona e mantenete la calma.*
- *Ascoltate la lamentela, mantenendo una mentalità aperta.*
- *Riformulare ciò che la persona ha detto.*
- *Accettare la responsabilità e scusarsi, se necessario.*

Esempi di role-play da usare:

- Qualcuno si lamenta con voi perché lo avete interrotto.
- Qualcuno si lamenta perché avete acceso una sigaretta sull'autobus.
- Il vostro case manager si lamenta del vostro ritardo all'appuntamento.
- Il vostro consulente alla residenza comunitaria si lamenta che non avete fatto le vostre faccende settimanali.
- Il vostro compagno di stanza si lamenta che la vostra musica è troppo alta.

Slide 11

Far sapere a qualcuno che non ci si sente sicuri e chiedere aiuto

Tutti noi, in qualche momento della nostra vita, ci sentiamo insicuri. Condividere le nostre paure con qualcuno di cui ci fidiamo di solito rende le cose meno spaventose. Questa persona potrebbe avere dei suggerimenti che vi aiuteranno ad affrontare la sensazione di insicurezza o che vi aiuteranno a cambiare la situazione di cui avete paura. La maggior parte delle persone, una volta o l'altra, si trova in situazioni che non può gestire da sola, situazioni in cui ha bisogno di chiedere aiuto agli altri. Spesso le persone si sentono a disagio o timide nel chiedere aiuto. Secondo la nostra esperienza, nella maggior parte dei casi le persone sono più che disposte a fornire aiuto quando glielo si chiede.

Come far sapere a qualcuno che non ci si sente sicuri:

- *Scegliete una persona di fiducia con cui parlare.*
- *Ditegli cosa vi fa sentire insicuri. Cercate di essere specifici sulle vostre paure.*
- *Chiedete a questa persona un consiglio.*

Come chiedere aiuto:

- *Scegliete una persona di cui sentite di potervi fidare.*
- *Usate una voce calma e chiara.*
- *Dite all'interlocutore di cosa avete bisogno di aiuto. Siate specifici.*
- *Ascoltate attentamente ciò che la persona vi suggerisce.*
- *Ringraziate la persona per il suo aiuto.*

Esempi di role-play da usare:

- Il paziente dice al suo case manager di sentirsi insicuro al centro diurno.
- Dite al membro dello staff che non vi sentite sicuri in mezzo alla folla e che non volete partecipare alla gita programmata.
- Dite al vostro compagno di stanza che non vi sentite sicuri a camminare nel quartiere di notte.
- Dite ai vostri familiari che non vi sentite sicuri con una nuova persona che è venuta a vivere nella vostra abitazione.
- Dite al vostro medico che non vi sentite sicuri nell'assumere i nuovi farmaci prescritti.
- Avete difficoltà a compilare una domanda per un lavoro di volontariato.
- Avete appena comprato un oggetto per la vostra stanza e vi rendete conto che è troppo pesante da trasportare da soli.
- Avete preso la metropolitana in centro e improvvisamente vi rendete conto di essere scesi alla fermata sbagliata e di esservi persi.
- State camminando per strada e qualcuno vi ruba il portafoglio o la borsa.

- State facendo una passeggiata ed inciampate, slogandovi la caviglia. Vi rendete conto che non sarete in grado di camminare fino a casa.

Slide 12

Abbandonare le situazioni di stress

A volte ci troviamo in situazioni che consideriamo stressanti. Per esempio, quando gli altri ci criticano o quando facciamo qualcosa che non piace agli altri. Spesso, rimanere in situazioni stressanti non fa altro che farci sentire peggio e a volte può addirittura aggravare la situazione. Spesso accade che andarsene fino a quando non ci si è calmati e affrontare la situazione in un secondo momento sia il modo più produttivo di gestire una situazione stressante. I seguenti passi sono utili per abbandonare una situazione di stress:

- *Determinare se la situazione è stressante (cioè, sintonizzarsi sui propri pensieri, sentimenti e sensazioni fisiche).*
- *Dite all'altra persona che la situazione è stressante e che dovete andarvene.*
- *Se c'è un conflitto, dite alla persona che ne parlerete in un altro momento.*
- *Abbandonate la situazione.*

Esempi di role-play da usare:

- Un parente vi ha accusato ingiustamente di aver rubato 10€.
- Un'amica è arrabbiata perché non volete andare al bar con lei.
- Un parente è arrabbiato perché ha ritrovato della droga nella tua stanza.
- Un membro dello staff della comunità residenziale è arrabbiato perché sei tornato a casa tardi e hai dimenticato di chiamarla per avvisarla.
- Il vostro compagno di stanza è arrabbiato perché avete indossato la sua camicia senza chiederla in prestito.

Slide 13

Dissentire dall'opinione altrui senza discutere

Non tutte le persone con cui entriamo in contatto saranno d'accordo con le nostre idee o opinioni, così come noi non siamo d'accordo con le loro. Il disaccordo non deve necessariamente portare a cattivi sentimenti o a una discussione. Anzi, la vita sarebbe noiosa se tutti avessero le stesse idee. Quando si è in disaccordo con l'opinione di un'altra persona, spesso le cose filano più lisce se si tengono a mente alcune punti:

- *Esprimete brevemente il vostro punto di vista.*
- *Ascoltate l'opinione dell'altro senza interromperlo.*

- *Se non siete d'accordo con l'opinione dell'altro, dite semplicemente che è giusto non essere d'accordo.*
- *Chiudete la conversazione o passate a un altro argomento.*

Esempi di role-play da usare:

- Voi e un amico avete opinioni diverse su un film che avete appena visto.
- Voi e il vostro compagno di stanza avete opinioni diverse su quale gruppo musicale sia migliore.
- Voi e un membro dello staff della comunità residenziale avete opinioni diverse sul tipo di abbigliamento che più vi dona.
- Voi e un membro della famiglia avete opinioni diverse su come festeggiare il vostro compleanno.
- Voi e un consulente non siete d'accordo su quale sia stata la cosa più utile per farvi trovare un lavoro.

Slide 14

Rispondere ad accuse non veritiere

La maggior parte di noi si è trovata in situazioni in cui è stata accusata di aver fatto qualcosa di non vero. Di solito, quando ciò accade, la persona che ci accusa crede davvero che abbiamo compiuto l'atto e non è in grado di ascoltare la ragione. Per questo è importante mantenere la calma e non litigare o discutere quando ciò accade. Riteniamo che ci siano alcuni accorgimenti specifici che possono aiutarvi a mantenere la calma quando si è accusati ingiustamente di qualcosa:

- *Con voce calma, negate semplicemente l'accusa.*
- *Se l'altra persona continua ad accusarvi, chiedetele di smettere.*
- *Se la persona non smette di accusarvi, ditele che chiederete a un membro del personale di aiutarvi a risolvere la situazione.*
- *Allontanarsi e chiedere assistenza, se necessario.*

Esempi di role-play da usare:

- Un coinquilino vi accusa di aver rubato i suoi vestiti dall'asciugatrice comune.
- Un coinquilino vi accusa di non aver svolto le faccende domestiche assegnate.
- Una persona al centro durno vi accusa di ascoltare le sue conversazioni.
- Un membro del personale della comunità residenziale vi accusa di aver iniziato una lite con un altro residente.
- Un parente vi accusa di aver rubato del denaro durante la vostra ultima visita.

Slide 15

Chiedere scusa

Anche quando le persone sono molto attente, a volte fanno cose che infastidiscono o disturbano gli altri. Piuttosto che ignorare la situazione o litigare, abbiamo riscontrato che in genere le cose vanno meglio se la persona si scusa per il suo comportamento il prima possibile. Questo vale indipendentemente da chi abbia commesso l'errore. I seguenti passi vi forniscono un modello di come scusarsi:

- *Guardare la persona.*
- *Dichiarate le vostre scuse: "Mi dispiace per_______ ".*
- *Se è realistico, assicurate alla persona che non succederà più in futuro.*

Esempi di role-play da usare:

- Arrivare in ritardo al gruppo perché si parla con un amico.
- Urtare qualcuno mentre si usa il distributore automatico.
- Interrompere qualcuno che sta parlando durante la cena.
- Prendere in prestito un CD senza chiedere al proprietario.
- Urlare a qualcuno quando si è di cattivo umore.

Slide 16

Fare ed accettare complimenti

Fare complimenti specifici è un buon modo per esprimere sentimenti positivi. Di solito i complimenti sono rivolti a qualcosa di visibile, come un capo di abbigliamento, un taglio di capelli o un paio di scarpe. Fare e ricevere complimenti fa sentire bene le persone. Oltre a saper fare i complimenti, è importante saper ricevere o accettare i complimenti dagli altri. Se accettate bene un complimento, è più probabile che le persone vi facciano altri complimenti in futuro. È importante non minimizzare o annullare un complimento.

Come fare dei complimenti:

- *Guardate la persona.*
- *Usate un tono positivo e sincero.*
- *Siate specifici su ciò che vi piace.*

Come accettare dei complimenti:

- *Guardate la persona.*
- *Ringraziate la persona.*
- *Rispondete al complimento:*

a. *Dicendo come vi ha fatto sentire*
b. *Esprimendo i propri sentimenti in merito all'oggetto del complimento.*

Esempi di role-play da usare:

- La persona A dice alla persona B che le piacciono le sue scarpe. La persona B accetta il complimento.
- La persona B dice alla persona C che le piace il colore della sua camicia. La persona C accetta il complimento.
- La persona C dice alla persona D che le piacciono i suoi jeans. La persona D accetta il complimento.
- La persona D dice alla persona E che le piace il suo taglio di capelli. La persona E accetta il complimento.
- La persona E dice alla persona A che le piace la sua acconciatura. La persona A accetta il complimento.

Prima di lasciarci, volete chiedermi qualcosa? Ci sono cose non chiare? Volete una copia del materiale che ho usato in questo incontro?

Sollecitare l'intervento di tutti

Rispondere ad eventuali domande

Ringraziare i presenti per la loro partecipazione, cercando di mettere in evidenza il contributo di ognuno.
