# Supplementary material for: Study protocol for a pilot randomized controlled trial on the feasibility and preliminary efficacy of an integrated psychoeducational intervention for transition-age youths in acute psychiatric settings
Source: Front Psychiatry. 2026 Feb 13;17:1768016. doi: 10.3389/fpsyt.2026.1768016 (PMC12946135; doi:10.3389/fpsyt.2026.1768016)
Supplement: Supplementary file 2 [file DataSheet2.pdf]

Supplementary Material 2. Satisfaction questionnaire.

Please indicate your level of agreement with each statement by circling the appropriate number: 1 = Not at all, 2 = A little, 3 = Fairly, 4 = Much, 5 = Very much

	**Not at all**	**A little**	**Fairly**	**Much**	**Very much**
**I found the group meeting interesting**	1	2	3	4	5
**I found the group meeting useful**	1	2	3	4	5
**The meeting helped me understand more about myself**	1	2	3	4	5
**The meetings helped me understand more about my disorder**	1	2	3	4	5
**What I learned will help me better manage moments of crisis**	1	2	3	4	5
**What I learned encouraged me to change some of my behaviors**	1	2	3	4	5
**I think participating in the meetings will have a positive impact on my life**	1	2	3	4	5
