# Supplementary material for: Study protocol for a pilot randomized controlled trial on the feasibility and preliminary efficacy of an integrated psychoeducational intervention for transition-age youths in acute psychiatric settings
Source: Front Psychiatry. 2026 Feb 13;17:1768016. doi: 10.3389/fpsyt.2026.1768016 (PMC12946135; doi:10.3389/fpsyt.2026.1768016)
Supplement: Supplementary file 3 [file DataSheet3.pdf]

AZIENDA OSPEDALIERO-UNIVERSITARIA
DELLE MARCHE
UNIVERSITÀ POLITECNICA DELLE MARCHE
OSPEDALI RIUNITI di ANCONA
DIPARTIMENTO DI SCIENZE NEUROLOGICHE
CLINICA di PSICHIATRIA

Modulo 1
"Emozioni di base"

Informazioni per gli utenti

In questa dispensa parleremo delle emozioni di base (o "primarie") e della loro funzione. Le emozioni si possono in generale definire fenomeni complessi e transitori che coinvolgono la mente ed il corpo e determinano comportamenti, funzionali o disfunzionali. Le emozioni accadono in risposta ad eventi esterni o interni, in quanto possono essere scatenate sia da particolari situazioni che dalla produzione di pensieri o ricordi.

Lo psicologo statunitense Paul Ekman ha definito 5 emozioni di base, caratterizzate del manifestarsi in tutti gli esseri umani, indipendentemente dal contesto culturale.

Esse possiedono un significato adattativo-evolutivo, in quanto hanno permesso ai nostri antenati di sopravvivere ed adattarsi, in maniera efficace e funzionale, all'ambiente in cui vivevano.

La teoria evoluzionistica di Charles Darwin considera le emozioni come processi adattivi che permettono di valutare il pericolo, agire, comunicare con i conspecifici ed adattarsi all'ambiente. Le emozioni, dunque, risultano fondamentali per fornire informazioni e garantire la sopravvivenza dell'individuo, assumendo un valore necessario e positivo per tutta la specie animale.

Le emozioni, inoltre, compaiono prima del linguaggio e del pensiero come forma di comunicazione (es. pianto di un neonato affamato) ed hanno una funzione adattiva, sono fondamentali cioè per la nostra sopravvivenza.

Le emozioni primarie sono :

- **gioia**
- **tristezza**
- **rabbia**
- **disgusto**
- **paura**

La combinazione delle emozioni primarie produce le emozioni "secondarie", fra le quali

riconosciamo vergogna, offesa, perdono, delusione, rimorso, speranza, e molte altre.
Le emozioni hanno un correlato nella mimica e nell'espressività facciale, permettendoci in tal modo di comunicare anche all'esterno il nostro stato d'animo.

Il riconoscimento e la regolazione delle emozioni giocano un ruolo importante per la nostra vita e per la salute fisica e mentale, in quanto condiziona in modo diretto il nostro benessere e la relazione con gli altri.

Quali sono le funzioni specifiche delle emozioni primarie?

**Emozione**	**Scopo**
**Gioia**	segnala che uno scopo è stato raggiunto<br>*esempio: ho ottenuto un buon voto a scuola*
**Tristezza**	segnala una perdita o un fallimento<br>*esempio: non ho superato un esame*
**Rabbia**	segnala un ostacolo, un danno, un torto o un'ingiustizia subita<br>*esempio: un amico ha rivelato una mia confidenza privata ad altre persone*
**Paura**	segnala una minaccia dalla quale difendersi o fuggire<br>*esempio: avverto una scossa di terremoto*
**Disgusto**	segnala la necessità di porre attenzione verso un oggetto/sostanza potenzialmente nocivi<br>*esempio: cibo che emana cattivo odore*

Tutte le emozioni necessitano di essere adeguatamente regolate, per non determinare eccessiva sofferenza o comportamenti maladattivi.
Per tale motivo, spesso le persone con difficoltà di regolazione emotiva, quando percepiscono emozioni spiacevoli e intollerabili, mettono in atto comportamenti poco vantaggiosi. Ad esempio, quando l'intensità della nostra rabbia è molto alta, potremmo mettere in atto comportamenti impulsivi e/o aggressivi, esponendo in situazioni di pericolo noi stessi o gli altri.

Riconoscere in che modo si esprimono le emozioni è fondamentale per il nostro benessere e per le nostre relazioni, perché ci permettono di comprendere quali sono i nostri bisogni e se stiamo o meno raggiungendo obiettivi per noi importanti.

Le emozioni possiedono **5 componenti**:

- cognitiva (pensiero)
- fisiologica (attivano il corpo)
- motivazionale (spingono all'azione)
- espressivo-motoria (attivano l'espressività facciale e i movimenti corporei)
- esperienziale (sono riferite ad una situazione/contesto specifici)

È del tutto naturale sperimentare tutta la gamma di emozioni, da quelle più piacevoli (ad esempio, gioia) a quelle meno piacevoli (ad esempio, rabbia), in quanto tutte le esperienze emotive sono utili, ci guidano nell'esplorazione dell'ambiente e nelle decisioni ed informano gli altri sui nostri stati interni.

Il **modello** "**ABC**" (dall'inglese: *antecedent*, *behaviour*, *consequences*), in tal senso, ci aiuta a mettere ordine all'interno delle nostre esperienze e ad annotare quali pensieri ci passano per la testa mentre proviamo una determinata emozione e come questa ci faccia reagire a livello emotivo e comportamentale.

Cosa ci insegna il modello ABC?

Le nostre emozioni non dipendono tanto da quello che ci accade, dalle situazioni o dagli eventi, quanto più dai nostri pensieri, dalle frasi che ci diciamo mentre siamo in una specifica situazione attivante. Le nostre emozioni dipendono, quindi, dall'interpretazione che noi facciamo dell'evento e non dall'evento in sé.

Bibliografia

- Beck, A.T. *Cognitive Therapy and the Emotional Disorders. Intl Universities Press, 1975.*

- Charles Darwin (1998). The Expression of the Emotions in Man and Animals, Oxford

University Press.

- Ellis, A. *(1957). Rational psychotherapy and individual psychology. Journal of Individual Psychology, 13, 38-44.*

- Ekman, P. (1984). Expression and the nature of emotion, in *Approaches to Emotion*, eds K. Scherer and P. Ekman (Hillsdale, NJ: Erlbaum).

- Ekman, P. (1970). Universal Facial Expressions of Emotions. *California Mental Health Research Digest.*

Modulo 2
"Ansia e tristezza"

Informazioni per gli utenti

In questa dispensa parleremo di *ansia e tristezza*, due emozioni che necessitano di essere adeguatamente regolate.

Le emozioni possono essere definite come stati affettivi intensi, di breve durata, che possono avere una causa interna o esterna, un contenuto cognitivo e comportamentale ed hanno la funzione di riorientare l'attenzione**.** Tutte le emozioni hanno un inizio, una durata ed una fase di attenuazione.

Ansia

L'ansia è l'emozione che si attiva in risposta ad una sensazione di minaccia reale o ipotetica. Essa rappresenta una risposta normale e innata di attivazione, caratterizzata da un aumento della vigilanza e dell'attenzione, che ha l'obiettivo di prepararci ad affrontare il pericolo percepito, predisponendoci alla cosiddetta risposta di attacco o fuga (fight-or-flight response).

L'ansia può essere fisiologica oppure patologica. L'ansia fisiologica ci prepara ad affrontare in maniera adattiva una possibile situazione minacciosa, mentre l'ansia patologica è disfunzionale perché, essendo persistente e intensa, interferisce con la nostra prestazione e può essere associata a eventi neutri, che non sono realmente pericolosi. Alcuni esempi di pensieri legati all'ansia sono*: riuscirò a parlare all'interrogazione? prenderò l'autobus in tempo? mi inviteranno alla festa?*

I sintomi dell'ansia sono i seguenti:

- *irrequietezza, sentirsi tesi, con i "nervi a fior di pelle"*
- *facile affaticamento*
- *difficoltà di concentrazione (o vuoti di memoria)*
- *irritabilità*
- *tensione muscolare*
- *alterazioni del sonno*
- *tachicardia*
- *sudori*
- *vertigini*

L'ansia è un'emozione naturale e fisiologica, ma diventa un problema quando:

- *inizia ad interferire con le nostre attività quotidiane*
- *ci impedisce di raggiungere i nostri obiettivi*
- *crea sintomi psico-fisici che durano nel tempo*

Quando si diventa eccessivamente ansiosi, accade inoltre che la capacità di risolvere problemi diminuisce, perchè l'ansia riduce la capacità di pensare ed agire in modo lucido e ragionevole.

Tristezza

L'emozione della tristezza segnala la perdita o il fallimento di uno scopo importante.

La sua funzione è quella di permettere all'organismo di ritrovare il proprio equilibrio e di proteggere la persona nei momenti di maggiore vulnerabilità.

Inoltre essa permette di recuperare le forze fisiche e psicologiche e richiama l'attenzione degli altri sul nostro accudimento.

La tristezza è un'emozione naturale e fisiologica, ma diventa un problema quando è presente:

- *umore depresso per la maggior parte del giorno, quasi tutti i giorni*
- *perdita di interesse o piacere per le attività che prima si trovavano piacevoli*
- *significativo cambiamento nel peso (perdita o aumento)*
- *insonnia o ipersonnia*
- *agitazione o rallentamento psicomotorio*
- *faticabilità o mancanza di energia*
- *sentimenti di autosvalutazione o di colpa eccessivi o inappropriati*
- *ridotta capacità di pensare o concentrarsi*
- *pensieri ricorrenti di morte*

La depressione è una tristezza profonda protratta nel tempo, caratterizzata da sentimenti e pensieri autosvalutanti, mancanza di speranza e senso di vuoto.

La depressione, inoltre, può esprimersi nella nostra mente con:

- *demotivazione*
- *pensieri negativi*
- *problemi di concentrazione*
- *difficoltà di memoria*
- *visione pessimistica della realtà*
- *senso di scarso valore e fallimento*

Alcuni pensieri tipici della depressione**:** s*ono un fallito/a, non valgo niente, non guarirò mai, è tutta colpa mia, niente sarà più come prima.*

Vi sono, inoltre, alcuni indicatori che aiutano a capire quando le emozioni sono fuori controllo e necessitano di attenzione medica:

- *intensità*
- *sproporzione rispetto alla situazione*
- *impedimento nel vivere la solita quotidianità*
- *durano a lungo*

Bibliografia

- American Psychiatric Association (2013). Diagnostic and statistical manual of mental disorders (5th ed.). Washington, DC: Author.

Intervento Psicoeducativo

Modulo 3

“Esperienze insolite”

Informazioni per utenti

In questa dispensa parleremo di alcuni sintomi delle malattie psichiatriche.

Con dissociazione si intende un meccanismo di difesa con cui alcuni elementi dei processi psichici rimangono "disconnessi" o separati dal restante sistema psicologico dell'individuo. Si verifica durante i disturbi d’ansia, i disturbi dell’umore, a seguito di traumi.

La derealizzazione si caratterizza per il sentimento di irrealtà, come se «nulla fosse reale». Si verifica durante i disturbi d’ansia ed i disturbi dell’umore.

La depersonalizzazione si caratterizza per una perdita della percezione di sé stessi. Può inoltre capitare che il soggetto veda sé stesso dall’alto o dall’esterno mentre svolge le attività quotidiane. Si verifica durante i disturbi d’ansia ed i disturbi dell’umore.

I disturbi psicotici rappresentano una delle grandi classi delle malattie psichiatriche. La schizofrenia ha rappresentato da sempre la malattia regina di questa branca della medicina, però non è l’unico disturbo facente parte di questa classe. Nei disturbi psicotici sono presenti anche altre affezioni: disturbo delirante, disturbo schizofreniforme, bouffée delirante e il disturbo schizoaffettivo. Si caratterizzano per la presenza dei seguenti sintomi:

- Sintomi positivi: ideazione delirante, allucinazioni (principalmente voci, rumori, suoni), alterazioni della forma del pensiero (pensiero rallentato, illogico)
- Sintomi negativi: affettività appiattita (provare meno sentimenti), ritiro sociale, difficoltà relazionali.

La presenza di sintomi di carattere psicotico però non indica necessariamente la presenza di un disturbo psicotico, i sintomi psicotici possono infatti essere rinvenuti nel contesto dei disturbi dell’umore o nei disturbi di personalità, ad esempio si possono avere episodi depressivi con sintomi psicotici o durante le riacutizzazioni del disturbo bipolare.

Le allucinazioni sono delle alterazione della percezione della realtà esterna. Si tratta della percezione di qualcosa non presente nell’ambiente, sperimentabile mediante uno dei cinque sensi (raramente possono essere più sensi). In genere nel contesto delle malattie psichiatriche, le allucinazioni più frequenti sono quelle uditive, si parla infatti spesso di voci. Ciò che differenzia un’allucinazione da una illusione è la presenza dell’oggetto esterno. Nelle illusioni, infatti, è la percezione di qualcosa reale ad essere alterata. Ad esempio, un oggetto può essere percepito più grande o più piccolo del normale.

Non tutte le allucinazioni sono patologiche. In particolare, esistono le allucinazioni ipnagogiche ed ipnopompiche, che si verificano rispettivamente durante la fase dell’addormentamento e durante il

risveglio. Esistono anche le allucinazioni che si verificano durante l'uso di sostanze, le quali però svaniscono una volta che l'effetto della droga termina. Le allucinazioni uditive sono un indice di profonda sofferenza psicologica e tendono a peggiorare sotto stress. Lo stress è infatti una fattore precipitante per questo tipo di sintomatologia. A volte sono un modo per far fronte alla solitudine o all'isolamento prolungato. Succede infatti che persone isolate da lungo tempo, possano iniziare a esperire questo tipo di allucinazioni.

Distrarsi aiuta a ridurre le presenza di tali dispercezioni. Ridurre o evitare i fattori stressanti, infatti, aiuta a prevenire il peggioramento di questa sintomatologia. È fondamentale parlare con qualcuno di cui ci si fida, questo aiuta ad attenuare la presenza delle voci e a riconoscerle come manifestazione derivante dal nostro pensiero.

Ridurre le voci è fondamentale per ridurre il dolore che queste provocano e favorire la comunicazione con gli altri.

Le strategie che possiamo attuare per la ridurre le voci sono:

1. Distrarsi ed impegnarsi in attività piacevoli (canticchiare, parlare con te stesso, ascoltare musica, pregare, meditare, usare un mantra, dipingere, camminare all'aria aperta, chiamare un amico, fare esercizio fisico, usare un cd di rilassamento, fare yoga, fare un bagno caldo, chiamare il proprio psichiatra, frequentare un centro diurno, guardare la tv, fare un cruciverba o un puzzle, giocare ad un gioco al computer, provare un nuovo hobby),
2. Scrivere o disegnare le voci, dialogare con le voci, focalizzarsi su quelle positive,
3. Dare alle voci uno spazio di dieci minuti in uno specifico orario ogni giorno,
4. Ricorda a te stesso che nessun altro può sentire la voce, usa una spiegazione normalizzante
5. Usa risposte razionali per ridurre la rabbia,
6. Elencare le prove contro il contenuto delle voci,
7. Riconoscere i fattori stressanti per poterli evitare o prevenire,
8. Assumere correttamente la terapia farmacologica,
9. Rimanere in contatto con lo psichiatra.

Con il termine delirio si intende un'idea o una convinzione completamente falsa con cui nessun altro può essere d'accordo e a cui qualcuno crede fermamente.

Ecco alcuni esempi di pensiero delirante:

- Pensare che una forza o un'altra persona controlli i suoi pensieri e/o le azioni,
- Pensare che ciò che vede o legge nasconda un messaggio segreto indirizzato a lui,
- Pensare di essere un persona speciale molto diversa da quella che è o di avere capacità veramente straordinarie.

Le principali cause che possono portare allo sviluppo di sintomi psicotici sono: un forte stress e l'uso di sostanze stupefacenti.

Le risposte sono ovvie e in linea con quelle già descritte per le allucinazioni:

- farmaci antipsicotici per favorire quelle condizioni mentali che consentono di restituire al paziente la capacità di riconoscere le proprie paure distinguendole dalla realtà (ricordiamo che il termine psicosi nel nostro lavoro è utilizzato per indicare la perdita di tale capacità della mente);
- contatto continuo con lo psichiatra per stabilire e verificare comportamenti efficaci;
- riconoscimento dei segni precoci di crisi; riconoscimento e gestione dello stress.

Bibliografia:

- Colom F. e Vieta E., Manuale di psicoeducazione per il disturbo bipolare, Giovanni Fioriti Editori, 2016
- Faloon I., Intervento psicoeducativo integrato in psichiatria, Erikson, 1992
- Siracusano A., Manuale di psichiatria, Il Pensiero Scientifico Editore, 2014

Intervento Psicoeducativo

Modulo 4

"Dipendenze"

Informazioni per gli utenti

In questa dispensa parleremo delle dipendenze.

Con il termine droga si intendono tutte quelle sostanze (legali o illegali) che hanno la capacità di modificare lo stato di coscienza, il comportamento, le emozioni, i pensieri e, allo stesso tempo, di creare condotte di dipendenza o abuso.

I motivi per cui inizialmente una persona può decidere di assumere una sostanza psicotropa possono essere tutti riconducibili a una qualche forma di piacere o all'evitamento di un disagio. In particolare, i motivi possono essere: rilassarsi, desiderare una gradevole eccitazione, svagarsi un po', disinibirsi e magari darsi coraggio, sentirsi maggiormente prestante, creativo o efficiente. Qualcuno potrebbe invece ricercare un temporaneo sollievo dall'ansia, dalla tristezza, dalla rabbia o da altri sentimenti indesiderati. Provare piacere è una capacità innata, mediata del cervello e comune agli umani. Così quando ad esempio mangi la torta che prediligi, quando raggiungi con impegno e sacrificio un obiettivo per te molto importante si attivano delle aree del cervello che costituiscono il cosiddetto della gratificazione e del piacere. Le cellule nervose di questo sistema rilasciano dopamina. Le sostanze psicotrope fanno invece un "trucco chimico", possono azionare questo meccanismo determinando il rilascio (o rallentando il riassorbimento) della dopamina nel sistema della gratificazione. In questo caso però, la disponibilità di dopamina sarà notevolmente aumentata e quindi sarà anche più intensa la stimolazione dei circuiti cerebrali.

Le sostanze più utilizzate sono: Alcol, Cannabinoidi, Cocaina, Eroina ed altri oppiodi, Stimolanti (MDMA, anfetamine etc.), Allucinogeni (LSD, psilocibina), Caffè, Tabacco, Sostanze sintetiche.

L'alcol genera una depressione a medio termine, portando anche ad un aumento delle quote ansiose, una destrutturazione del sonno, un discontrollo degli impulsi, un deterioramento cognitivo ed una slatentizzazione di sintomi psicotici. La cannabis genere quella che viene chiamata sindrome amotivazionale con conseguente apatia, depressione, alterazione del sonno, abulia, astenia. Può inoltre provocare uno switch maniacale nei pazienti bipolari o causare episodi psicotici. La cocaina, oltre ad avere degli effetti devastanti come l'eroina sul piano fisico, porta ad un aumento di ansia, aggressività, sintomi psicotici, deterioramento cognitivo, switch maniacali, alterazioni del sonno. L'eroina ha un quadro sovrapponibile a quello della cocaina come effetti a lungo termine. Gli allucinogeni e le droghe di sintesi possono causare l'insorgenza di episodi psicotici ed eventualmente indurre uno switch maniacale. I sintomi allucinatori inoltre possono persistere per lunghissimo periodo, generando dei fenomeni detti "flashback". Il caffè ha come effetto quello di alterare il ritmo del sonno, elemento fondamentale per una buona salute mentale. Può anche incrementare l'ansia e indurre attacchi di panico. Il tabacco non ha grandi rischi sul piano psichiatrico, ma sono soprattutto di natura fisica.

Il piacere è una qualità emotiva che ci motiva a ripetere ciò che lo ha procurato. Sembra infatti che, nel corso dell'evoluzione della specie, la selezione naturale abbia fatto emergere il piacere come meccanismo per incentivare comportamenti importanti per la sopravvivenza (come ad esempio mangiare, riprodursi, raggiungere un obiettivo). Il piacere e l'assunzione indotto droghe funge da incentivo (rinforzo) per le assunzioni successive, favorendo il ripetersi del consumo. Tuttavia, le conseguenze possono essere deleterie per la vita e col tempo possono ridurre, paradossalmente, la possibilità di provare piacere. Questo meccanismo determina (per motivi genetici, di genere, biologici, psicologici e ambientali) l'instaurarsi della dipendenza. Se l'assunzione delle sostanze continua, il cervello riduce la sua sensibilità alla dopamina quando la concentrazione diventa eccessiva: di conseguenza se ne produce meno, rendendo meno piacevoli tutte quelle attività che prima erano considerate gratificanti e piacevoli. L'unico modo per aumentare i livelli di dopamina sarà assumere la sostanze psicoattiva. Questo spinge la persona ad un progressivo disinteresse verso tutte le attività piacevoli della vita, portando allo sviluppo di sintomi depressivi. A questo si associa il fenomeno di tolleranza, per cui è necessario assumere dosi sempre maggiori della droga per ottenere lo stesso effetto. Tutto questo porta poi a problematiche familiari, lavorative, sociali, finanziarie, legali e mediche, che a loro volta spingono alla nuova assunzione di sostanza (generando così un circolo vizioso).

Nel tempo, il piacere della sostanza tende a diminuire per effetto della tolleranza mentre potrebbero presentarsi delle sensazioni fisiche indesiderate (ad esempio nausea, tremori, mal di testa, sudorazione profusa) e delle emozioni spiacevoli (come ansia, nervosismo, depressione) se le droghe o l'alcol non sono assunte costantemente. Sono i sintomi astinenziali: effetti fastidiosi o dolorosi che possono rappresentare un ulteriore stimolo al consumo della sostanza nel tentativo di riceverne sollievo.

Con il progressivo uso la corteccia prefrontale (deputata a modulare ed inibire le emozioni ed i comportamenti impulsivi) viene alterata, facilitando gli automatismi e l'impulsività. Le sostanze, inoltre, possono attivare delle aspettative, le quali determinano l'insorgenza di craving. L'autocontrollo verrà così annullato da una serie di pensieri che giustificheranno e/o minimizzeranno l'idea di assumere la sostanza (ad esempio "Questa è l'ultima", "ogni tanto ci può stare"). Viene inoltre compromessa la capacità di orientare e mantenere l'attenzione, valutare razionalmente i vantaggi e gli svantaggi di una scelta, organizzare pensieri e attività ed, infine, di prendere decisioni.

Le tecniche da utilizzare per affrontare il problema delle dipendenze sono le seguenti: Ripetersi frequentemente quali sono gli svantaggi fisici, sociali e psicologici del bere, Frequentare gruppi di supporto, Chiedere aiuto a qualcuno di cui ci si fida in caso di difficoltà, Rivolgersi ad uno psichiatra e/o psicologo.

Esistono però anche dipendenze sul piano comportamentale. Le dipendenze comportamentali sono: gioco d'azzardo, shopping compulsivo, dipendenze digitali, da lavoro, sessuale, da esercizio fisico.

Le dipendenze digitali stanno diventando sempre più frequenti negli anni. L'uso dei vari dispositivi diventa una dipendenza quando questo altera il funzionamento della persona.

Bibliografia:

- Colom F. e Vieta E., Manuale di psicoeducazione per il disturbo bipolare, Giovanni Fioriti Editori, 2016
- Lovato M. e Maddalon D., Affrontare la dipendenza. Strategie cognitivo-comportamentali per fronteggiare il disturbo da uso di sostanze

Intervento Psicoeducativo

Modulo 5

"Sonno"

Informazioni per utenti

In questa dispensa parleremo del sonno, un processo importante del quale prendersi cura, che condiziona il nostro umore e la nostra qualità di vita.

Il sonno può essere definito come una necessità fisiologica, uno stato di riposo contrapposto alla veglia. Esso riguarda una periodica sospensione dello stato di coscienza, durante il quale l'organismo recupera energia e risorse.

Il sonno, inoltre, è caratterizzato dal distaccamento temporaneo della coscienza e della volontà, dal rallentamento delle funzioni neurovegetative, nonchè dall'interruzione parziale dei rapporti sensomotori del soggetto con l'ambiente.

Alcuni accorgimenti comportamentali possono facilitare il sonno, migliorarne la qualità ed aiutare a prevenire l'insorgenza dei disturbi correlati.

Conoscere le regole di igiene del sonno, permette ai pazienti di adottare buone abitudini che consentono di aumentare la qualità del sonno, sia dal punto di vista qualitativo che quantitativo.

Le regole di igiene del sonno sono le seguenti:

1) Mettersi a letto soltanto se si ha veramente sonno.

2) Associare il letto unicamente al sonno e creare delle routine pre-addormentamento.

3) Mantenere orari di addormentamento e risveglio regolari.

4) Evitare sonnellini diurni.

5) Non assumere alcolici nelle 2-3 ore precedenti il periodo di sonno.

6) Evitare l'assunzione di sostanze eccitanti 6 ore prima di andare a dormire. Devono essere evitate tutte le sostanze stimolanti (coca cola, thè, caffè, etc.).

7) Non fumare nell'ultima mezz'ora prima di andare a dormire.

8) Non mangiare cioccolata/zuccheri.

9) Non bere grandi quantità di liquidi prima di andare a dormire.

10) Non praticare attività fisica prima di andare a dormire.

11) Rendi la camera confortevole e priva di rumori e/o luci fastidiose.

12) Mantieni la temperatura dell'ambiente moderata.

13) Non guardare l'orologio.

14) Se si è ancora completamente svegli 15-20 minuti circa dopo essersi messi a letto, alzarsi per fare qualcosa di diverso (ad esempio leggere un libro noioso).

15) Quando possibile, eliminare l'esposizione alla luce artificiale brillante.

Bibliografia

- Carskadon, M. A., & Dement, W. C. (2005). Normal human sleep: an overview. *Principles and practice of sleep medicine*, *4*(1), 13-23.
- Hauri, P. J. (1991). Sleep hygiene, relaxation therapy, and cognitive interventions. *Case studies in insomnia*, 65-84.

Intervento Psicoeducativo

Modulo 6

"Le Cure"

Informazioni per utenti

In questa dispensa parleremo dei principali trattamenti farmacologici.

Le terapie attualmente in uso nel contesto psichiatrico sono le seguenti:

- Psicoterapia,
- Terapia farmacologica,
- ECT, TMS, fototerapia,
- VNS, stimolazione intracranica
- Terapie di tipo «ludico» (es. musicoterapia, art-therapy etc.)

Il trattamento dei disturbi psichiatrici giova particolarmente di un trattamento combinato tra psicoterapia e terapia farmacologica. Non tutti gli approcci psicoterapici sono scientificamente validi. Quelli con maggiori evidenze scientifiche sono rappresentati dal cognitivo-comportamentale, sistemico-relazionale ed interpersonale. Allo stesso tempo la sola psicoterapia non è adatta al trattamento di disturbi psichiatrici quali disturbi psicotici o disturbi bipolare, patologie che hanno necessariamente bisogno di una terapia. La combinazione di entrambi questi interventi terapeutici è sinergica, in quanto lo psicofarmaco può aiutare a stabilizzare il farmaco consentendo allo psicoterapeuta di agire. Allo stesso tempo, la psicoterapia induce un miglioramento della compliance al farmaco e consolida il miglioramento ottenuto farmacologicamente.

Gli psicofarmaci possono essere classificati in quattro principali categorie: gli antipsicotici, che vanno ad agire sui sintomi psicotici; gli antidepressivi, che migliorano l'umore; gli ansiolitici, il cui ruolo è quello di ridurre la sintomatologia ansiosa e l'agitazione, ed infine i regolatori dell'umore, i quali aiutano a ridurre le oscillazioni del tono dell'umore. È fondamentale però non fossilizzarsi sui nomi, in quanto farmaci di una specifica classe possono avere degli effetti appartenenti ad altre categorie. Ad esempio, la quetiapina a seconda del dosaggio può essere ansiolitico, regolatore dell'umore e antipsicotico.

Gli psicofarmaci agiscono regolando i neurotrasmettitori. I principali neurotrasmettitori coinvolti sono la noradrenalina, che generalmente facilita la razione agli eventi stressanti della vita, la serotonina, presente nelle aree del cervello che regolano i ritmi dell'organismo, come il sonno e la veglia, la temperatura, l'appetito e i comportamenti sessuali, la dopamina, che ha un ruolo molto importante nel movimento. I neurotrasmettitori hanno un ruolo quindi particolarmente importante, in quanto permettono alle cellule del SNC di comunicare. In realtà, nel nostro organismo le cose sono più complesse di come sembrano, perché questi neurotrasmettitori interagiscono tra di loro e con altre sostanze chimiche e hanno effetto anche in altre zone del nostro organismo. Per questo motivo, ad esempio, gli antidepressivi riducono i sintomi della depressione, ma possono dare anche effetti collaterali, legati alla loro azione su altri sistemi.

La durata del trattamento farmacologico varia a seconda di numerosi fattori: il tipo di disturbo, la gravità della malattia, la risposta alla terapia ed eventuali trattamenti associati. Il trattamento non deve mai essere sospeso autonomamente ed ogni cambiamento posologico o del tipo di farmaco deve essere concordato con lo psichiatra di riferimento. Ad esempio, nel caso della depressione chi ha avuto un primo episodio depressivo, il rischio di averne un altro è particolarmente alto nei sei mesi successivi. Per questo, dopo un primo episodio, conviene continuare la terapia per almeno sei mesi dalla scomparsa dei sintomi, senza modificare le dosi. Se si smette di prendere gli antidepressivi prima di 6 mesi dalla scomparsa dei sintomi, il rischio di avere una ricaduta nell'anno successivo è tra il 35 e il 60%, mentre se queste medicine sono prese regolarmente per almeno sei mesi, il rischio è più basso, tra il 10 e il 25%. Le persone che, invece, hanno avuto tre o più ricadute devono continuare la cura per almeno 2 anni per evitare ulteriori ricadute.

L'assunzione in maniera regolare della terapia permette di mantenere stabili i livelli nel sangue del farmaco. Questo aiuta a ridurre gli effetti collaterali, migliora l'efficacia del farmaco e riduce le ricadute e le ospedalizzazioni. Risulta importante in questo caso l'uso della terapia Long Acting (LAI) che permette di mantenere molto più stabile il livello di farmaco nel sangue, evitando anche gli effetti di picco della terapia e allo stesso tempo migliorando la compliance e l'aderenza.

<u>**Non bisogna mai sospendere autonomamente la terapia psicofarmacologica**</u>.

I rischi legati alla sospensione possono essere riassunti nei seguenti:

- Brusco peggioramento del quado clinico,
- Ospedalizzazione,
- Riduzione dell'efficacia del farmaco durante una successiva esposizione dopo la sospensione.

Gli effetti collaterali più comuni degli psicofarmaci sono:

- Bocca secca, sudorazione eccessiva,
- Sonnolenza, astenia, alterazioni del ritmo cardiaco, insonnia,
- Disturbi sessuali,
- Mal di testa, problemi di vista,
- Nausea, vomito, aumento o diminuzione di peso, stitichezza o diarrea,
- Palpitazioni, pressione bassa, vertigini,
- Irritabilità, tremori.

Quando insorge uno degli effetti collaterali farmacologici la prima cosa da fare è quella di contattare lo psichiatra di riferimento, il quale metterà in atto una delle possibili strategie per ridurre il sintomo. In particolare, la prima cosa che si consiglia è di aspettare perché molti effetti collaterali sono passeggeri e tendono ad attenuarsi da soli nel tempo o a scomparire del tutto. Per questo motivo non conviene interrompere il trattamento se si hanno dei fastidi, ma parlarne con il proprio medico e aspettare qualche giorno prima di decidere cosa fare. Per alcune terapie farmacologiche c'è la possibilità di dosare il livello ematico del sangue. Conseguentemente questo permetterebbe di valutare si i livelli ematici sono troppo elevati, portando così allo sviluppo di alcuni effetti collaterali. Un'altra metodica consisterebbe nel ridurre la dose farmacologica in comune accordo con lo specialista di riferimento. Infatti, La risposta di una persona a un farmaco è individuale, cioè ognuno

reagisce un po' diversamente dagli altri. Il medico prescriverà all'inizio una dose che probabilmente avrà bisogno di piccoli aggiustamenti a seconda di come l'organismo reagisce. Per esempio, i disturbi sessuali tendono ad essere dose-dipendenti oltre che a scomparire con la sospensione del farmaco.

Alcune persone sono più sensibili a sviluppare un effetto collaterale piuttosto che un altro. Se dopo aver aspettato almeno un mese, o aver ridotto la dose, gli effetti collaterali non si riducono, il medico potrà decidere di sostituire il farmaco con un altro. Nella fase di cambiamento, però, è possibile che la persona abbia più effetti collaterali, quelli propri del farmaco che si sta gradualmente sospendendo e quelli del nuovo.

Per alcuni effetti collaterali è possibile usare dei rimedi molto semplici. Per esempio, la sonnolenza, che in genere scompare in pochi giorni da sola, se è molto fastidiosa può essere superata prendendo l'antidepressivo la sera, così da sfruttare questo effetto per l'insonnia. Viceversa, se l'antidepressivo dà insonnia, conviene prenderlo al mattino.

Per le vertigini dovute a bassa pressione, spesso basta alzarsi lentamente, mentre per la bocca secca può essere utile mangiare ogni tanto una caramella. La stitichezza è un effetto molto frequente. Il metodo più efficace è quello di fare attenzione alla dieta mangiando più verdura e frutta. Se il problema non si risolve, si può chiedere al medico di prescrivere una tisana lassativa o un altro preparato simile. Anche la diarrea tende ad attenuarsi da sola nel tempo e risponde bene ai fermenti lattici.

A volte per determinati effetti collaterali, il medico prescriverà dei farmaci aggiuntivi. Per esempio, se l'ansia è molto forte, prescriverà degli ansiolitici. Questi farmaci comunque vanno presi per brevi periodi perché può essere difficile riuscire a sospenderli se li si prende per molti mesi di seguito.

Bibliografia:

- Colom F. e Vieta E., Manuale di psicoeducazione per il disturbo bipolare, Giovanni Fioriti Editori, 2016
- Stahl S. M., Psicofarmacologia essenziale. Guida alla prescrizione, 2021
- Stahl S. M., Neuro Psicofarmacologia essenziale. Basi neuroscientifiche e applicazioni pratiche, 2016

Modulo 7

“Abilità comunicative”

Informazioni per gli utenti

In questa dispensa parleremo delle abilità comunicative, in particolare del saper comunicare emozioni piacevoli e spiacevoli, di come fare richieste in maniera positiva e di come mettere in atto l'ascolto attivo.

I problemi che incontriamo nella nostra vita quotidiana possono essere sia di tipo pratico che di natura interpersonale, come ad esempio le incomprensioni che si possono creare tra due persone.

Avere un problema con un'altra persona, genera uno stato di tensione che contribuisce a generare stress.

Le relazioni interpersonali sono influenzate positivamente o negativamente dalla capacità di comunicare e il vantaggio del miglioramento di tale abilità crea una maggiore sensazione di benessere.

La relazione tra due persone ha buona possibilità di migliorare se si utilizza una comunicazione adeguata. Infatti, è sempre possibile esprimere qualsiasi tipo di opinione, critica o richiesta, senza mai esprimere giudizi sulla persona.

Comunicare emozioni piacevoli

Nella vita di tutti i giorni c'è sempre qualcuno che ha fatto qualcosa di buono e positivo per l'altro, anche se spesso non gli diamo troppa importanza.

Tuttavia, risulta importante, saper comunicare le emozioni piacevoli all’altro in modo da valorizzare il suo comportamento e massimizzare la possibilità che venga di nuovo messo in atto.

Come dire ad una persona che ci è piaciuto il suo modo di fare?

- guardala negli occhi
- assumi una mimica e una postura adeguata al sentimento provato
- dille cosa ha fatto che ti è piaciuto
- dille come ti sei sentito (comunicare l'emozione)

Comunicare emozioni spiacevoli

Saper esprimere in maniera efficace i sentimenti spiacevoli, favorisce nel futuro che il comportamento dell'altro che ci ha ferito non si ripeta e dimostra che siamo disponibili e predisposti a chiarire la situazione.

Nella vita quotidiana, possono capitare spesso episodi in cui possiamo sentirci amareggiati, irritati, delusi dal comportamento altrui.

Alcune persone, per esempio, tendono a rimuginare e a non parlare con il diretto interessato e questo genera spesso un crescente risentimento verso quella persona.

Come dire ad una persona che non ci è piaciuto il suo modo di fare?

- guardala negli occhi
- assumi una mimica facciale e una postura adeguate al sentimento che stai per esprimere
- dille cosa ha fatto che non ti è piaciuto
- dille come ti sei sentito (comunicare l'emozione)
- proponi una soluzione per evitare che la situazione accada in futuro; suggerisci anche come poter modificare quel comportamento.

Fare richieste in maniera positiva

Saper avanzare una richiesta in maniera positiva è un'abilità che permette di ottenere ciò che si desidera, senza farsi fraintendere dall'altra persona.

In tal modo si massimizza la possibilità di ottenere quanto richiesto e si mantiene una relazione positiva con l'altro.

Come fare richieste in maniera positiva?

- guarda la persona negli occhi
- assumi una mimica e una postura adeguate
- digli in modo preciso cosa vorresti facesse per te
- digli cosa proveresti e come ti sentiresti se facesse quanto gli/le hai richiesto

Ascolto attivo

Saper ascoltare in modo attivo, cioè con attenzione e partecipazione, migliora la relazione tra le persone e fa capire che siamo interessati all'altro.

Come mettere in atto l'ascolto attivo?

- guardala la persona negli occhi
- assumi una mimica facciale ed una postura adeguate (es. cenni con il capo)
- fagli capire che sei attento a ciò che dice
- fai domande di chiarimento
- sintetizza quanto detto con le parole dell'altro

Bibliografia

• Falloon, I. (1993). *Intervento psicoeducativo integrato in psichiatria*. Edizioni Erickson.

Intervento Psicoeducativo

Modulo 8

"Problem Solving"

Informazioni per gli utenti

In questa dispensa parleremo della tecnica del problem solving, la quale vi aiuterà ogni volta che dovete prendere una decisione.

I problemi che incontriamo nella nostra vita quotidiana possono essere sia di tipo pratico che di natura interpersonale. Avere un piano di azione da seguire per far fronte a tali difficoltà vi aiuterà a non prendere decisioni in maniera impulsiva.

La tecnica si compone dei seguenti passi:

- Precisare qual è il problema o l'obiettivo, scegliere inizialmente degli obiettivi semplici e poi renderli sempre più complessi.
- Elencare più soluzioni senza commentarle, bisogna sospendere il giudizio e includere nella lista anche le soluzioni più bizzarre o strane.
- Discutere brevemente i vantaggi e gli svantaggi di ogni soluzione.
- Scegliere la soluzione "migliore", sia in termini di risorse impiegabili che di attuabilità.
- Fare un piano per metterla in pratica.
- Verificare se il piano funziona.

Cerca di memorizzare i punti che lo compongono e di utilizzare la tecnica nel contesto della vita quotidiana.

Intervento Psicoeducativo

Modulo 9

"Social Skill"

Informazioni per gli utenti

In questa dispensa parleremo delle tecniche da utilizzare per migliorare le vostre abilità sociali. Queste, infatti, possono essere alterate quando una persona soffre di depressione e ansia.

Iniziare una conversazione con uno sconosciuto

Sono molte le situazioni in cui si desidera iniziare una conversazione con un'altra persona. Può trattarsi di una persona che non conoscete bene o di una persona che non avete mai incontrato ma che vorreste conoscere. A volte le persone si sentono timide nell'iniziare una conversazione. Ci accorgiamo che le cose vanno meglio quando si tengono a mente alcuni passaggi specifici:

- Scegliete il momento e il luogo giusto.
- Se non conoscete la persona, presentatevi. Se si conosce la persona, dite "Ciao".
- Scegliete un argomento di cui volete parlare o fate una domanda.
- Giudicate se l'interlocutore vi ascolta e vuole parlare.

Continuare le conversazioni

A volte si vuole andare oltre una breve conversazione; si desidera parlare più a lungo con una persona perché questa ci piace o perché siamo interessati a ciò che è stato detto. Spesso le persone non sanno come mantenere una conversazione o si sentono a disagio. Un modo per continuare a parlare è fare domande. Un altro modo è quello di fornire informazioni concrete all'altra persona. Questo permette alle persone di conoscere meglio l'altro e le cose che potrebbero avere in comune. Le informazioni concrete sono quelle che dicono chi, cosa, dove, quando e come. Un ulteriore modo è quello di dire a qualcuno come ci si sente. Questo permette alle persone di conoscere meglio i sentimenti dell'altro e di capire se hanno più cose in comune di cui parlare. Esempi di sentimenti che possono essere espressi sono: felice, triste, eccitato, deluso, contento, turbato e irritato. Seguite i seguenti passaggi:

- Salutate la persona.
- Usate le seguenti tecniche per continuare la conversazione:
  - Fate una domanda su qualcosa che vorreste sapere.
  - Condividete alcune informazioni su un argomento che vorresti discutere.
  - Fate una breve descrizione di come vi fa sentire qualcosa.
- Valutare se la persona sta ascoltando ed è interessata a proseguire la conversazione.

Concludere una conversazione

Le conversazioni non vanno avanti per sempre, prima o poi qualcuno dovrà terminarla. Molte volte può capitare che spetti proprio a voi concluderla. Ci sono molte ragioni per terminare una conversazione, tra cui la mancanza di tempo, la necessità di andare da qualche altra parte o la mancanza di cose da dire. È possibile terminare le conversazioni in modo più agevole se si tengono

a mente alcuni passi:

- Aspettate che l'interlocutore abbia finito di parlare.
- Utilizzate un segnale non verbale, come uno sguardo distratto o un'occhiata all'orologio.
- Fate un commento conclusivo come "Beh, ora devo proprio andare".
- Dite: "Arrivederci".

Partecipare ad una conversazione già in corso

Ci sono momenti in cui tutti vogliono partecipare ad una conversazione in corso. Può trattarsi di argomenti interessanti, del desiderio di condividere un'opinione o forse solo del bisogno di far parte di un gruppo. Molte persone hanno difficoltà a sapere esattamente come farlo in modo da non interrompere il "flusso" della conversazione. I seguenti passi sono utili per entrare in una conversazione in corso:

- Aspettate una pausa nel flusso della conversazione.
- Dite qualcosa come "Posso unirmi a voi?".
- Decidete se le persone impegnate nella conversazione sono d'accordo che vi uniate a loro.
- Dite cose attinenti all'argomento della conversazione.

Rimanere sull'argomento stabilito da un'altra persona

Quando si è in una conversazione con un'altra persona, è importante dimostrare di prestare attenzione a ciò che viene detto. Riuscire a rimanere concentrati sull'argomento in discussione dimostra all'interlocutore che si sta ascoltando e che si è interessati a ciò che viene detto. I seguenti passi sono utili per rimanere sull'argomento di discussione in corso:

- Decidere quale sia l'argomento ascoltando la persona che sta parlando.
- Se dopo l'ascolto non si capisce quale sia l'argomento, chiedere all'interlocutore.
- Dite cose correlate all'argomento.

Cosa fare quando una persona va fuori argomento

Per sostenere una conversazione è necessario che entrambe le persone capiscano l'argomento, permettendo a ciascuno di contribuire con il proprio pensiero e rendendola più significativa per entrambi. A volte, però, ci si ci troviamo in una situazione in cui l'altra persona si è improvvisamente allontanata dall'argomento discussione, lasciandoci confusi. Quando ciò accade, è meglio far capire immediatamente all'interlocutore che siamo confusi e poi cercare di tornare all'argomento originale:

- Dite qualcosa del tipo: "È interessante; possiamo parlarne dopo aver finito questa discussione?".
- Se la persona ha dimenticato qual è l'argomento, ricordateglielo gentilmente.
- Valutate se l'interlocutore è ancora interessato all'argomento iniziale.
- Se l'interlocutore è interessato, continuate la discussione. Se non è interessato, chiudete gentilmente la conversazione o parlate di qualcosa di nuovo.

Formulare una lamentela

Molte situazioni spiacevoli possono essere evitate esprimendosi chiaramente e facendo richieste in modo positivo. Tuttavia, spesso si verificano situazioni in cui accade qualcosa di spiacevole. In questi

casi è necessario presentare un reclamo. Una lamentela di solito funziona meglio se si può anche suggerire una soluzione:

- Guardate la persona.
- Parlate con fermezza e calma.
- Esprimete il vostro reclamo. Siate specifici in merito alla situazione.
- Dite all'interlocutore come si potrebbe risolvere il problema.

Rispondere ad una lamentela

Per quanto possiate cercare di essere attenti e premurosi, ci saranno momenti in cui qualcuno dovrà presentare una lamentela nei vostri confronti. Ad esempio, si urta per sbaglio qualcuno o si dimentica un appuntamento. Se vi arrabbiate quando qualcuno si lamenta con voi, non farete altro che peggiorare la situazione. Seguendo le indicazioni che seguono si potrà affrontare la situazione in modo pacato:

- Guardate la persona e mantenete la calma.
- Ascoltate la lamentela, mantenendo una mentalità aperta.
- Riformulare ciò che la persona ha detto.
- Accettare la responsabilità e scusarsi, se necessario.

Far sapere a qualcuno che non ci si sente sicuri e chiedere aiuto

Tutti noi, in qualche momento della nostra vita, ci sentiamo insicuri. Condividere le nostre paure con qualcuno di cui ci fidiamo di solito rende le cose meno spaventose. Questa persona potrebbe avere dei suggerimenti che vi aiuteranno ad affrontare la sensazione di insicurezza o che vi aiuteranno a cambiare la situazione di cui avete paura. La maggior parte delle persone, una volta o l'altra, si trova in situazioni che non può gestire da sola, situazioni in cui ha bisogno di chiedere aiuto agli altri. Spesso le persone si sentono a disagio o timide nel chiedere aiuto. Secondo la nostra esperienza, nella maggior parte dei casi le persone sono più che disposte a fornire aiuto quando glielo si chiede.

Come far sapere a qualcuno che non ci si sente sicuri:

- Scegliete una persona di fiducia con cui parlare.
- Ditegli cosa vi fa sentire insicuri. Cercate di essere specifici sulle vostre paure.
- Chiedete a questa persona un consiglio.

Come chiedere aiuto:

- Scegliete una persona di cui sentite di potervi fidare.
- Usate una voce calma e chiara.
- Dite all'interlocutore di cosa avete bisogno di aiuto. Siate specifici.
- Ascoltate attentamente ciò che la persona vi suggerisce.
- Ringraziate la persona per il suo aiuto.

Abbandonare le situazioni di stress

A volte ci troviamo in situazioni che consideriamo stressanti. Per esempio, quando gli altri ci criticano o quando facciamo qualcosa che non piace agli altri. Spesso, rimanere in situazioni stressanti non fa altro che farci sentire peggio e a volte può addirittura aggravare la situazione. Spesso accade che

andarsene fino a quando non ci si è calmati e affrontare la situazione in un secondo momento sia il modo più produttivo di gestire una situazione stressante. I seguenti passi sono utili per abbandonare una situazione di stress:

- Determinare se la situazione è stressante (cioè, sintonizzarsi sui propri pensieri, sentimenti e sensazioni fisiche).
- Dite all'altra persona che la situazione è stressante e che dovete andarvene.
- Se c'è un conflitto, dite alla persona che ne parlerete in un altro momento.
- Abbandonate la situazione.

Dissentire dall'opinione altrui senza discutere

Non tutte le persone con cui entriamo in contatto saranno d'accordo con le nostre idee o opinioni, così come noi non siamo d'accordo con le loro. Il disaccordo non deve necessariamente portare a cattivi sentimenti o a una discussione. Anzi, la vita sarebbe noiosa se tutti avessero le stesse idee. Quando si è in disaccordo con l'opinione di un'altra persona, spesso le cose filano più lisce se si tengono a mente alcune punti:

- Esprimete brevemente il vostro punto di vista.
- Ascoltate l'opinione dell'altro senza interromperlo.
- Se non siete d'accordo con l'opinione dell'altro, dite semplicemente che è giusto non essere d'accordo.
- Chiudete la conversazione o passate a un altro argomento.

Rispondere ad accuse non veritiere

La maggior parte di noi si è trovata in situazioni in cui è stata accusata di aver fatto qualcosa di non vero. Di solito, quando ciò accade, la persona che ci accusa crede davvero che abbiamo compiuto l'atto e non è in grado di ascoltare la ragione. Per questo è importante mantenere la calma e non litigare o discutere quando ciò accade. Riteniamo che ci siano alcuni accorgimenti specifici che possono aiutarvi a mantenere la calma quando si è accusati ingiustamente di qualcosa:

- Con voce calma, negate semplicemente l'accusa.
- Se l'altra persona continua ad accusarvi, chiedetele di smettere.
- Se la persona non smette di accusarvi, ditele che chiederete a un membro del personale di aiutarvi a risolvere la situazione.
- Allontanarsi e chiedere assistenza, se necessario.

Chiedere scusa

Anche quando le persone sono molto attente, a volte fanno cose che infastidiscono o disturbano gli altri. Piuttosto che ignorare la situazione o litigare, abbiamo riscontrato che in genere le cose vanno meglio se la persona si scusa per il suo comportamento il prima possibile. Questo vale indipendentemente da chi abbia commesso l'errore. I seguenti passi vi forniscono un modello di come scusarsi:

- Guardare la persona.
- Dichiarate le vostre scuse: "Mi dispiace per_______ ".
- Se è realistico, assicurate alla persona che non succederà più in futuro.

Fare ed accettare complimenti

Fare complimenti specifici è un buon modo per esprimere sentimenti positivi. Di solito i complimenti sono rivolti a qualcosa di visibile, come un capo di abbigliamento, un taglio di capelli o un paio di scarpe. Fare e ricevere complimenti fa sentire bene le persone. Oltre a saper fare i complimenti, è importante saper ricevere o accettare i complimenti dagli altri. Se accettate bene un complimento, è più probabile che le persone vi facciano altri complimenti in futuro. È importante non minimizzare o annullare un complimento.

Come fare dei complimenti:

- Guardate la persona.
- Usate un tono positivo e sincero.
- Siate specifici su ciò che vi piace.

Come accettare dei complimenti:

- Guardate la persona.
- Ringraziate la persona.
- Rispondete al complimento:
    a. Dicendo come vi ha fatto sentire
    b. Esprimendo i propri sentimenti in merito all'oggetto del complimento.

Bibliografia:

- Giuseppe Nicolò e Laura Bernabie, Social skills training. Una guida operativa nei disturbi mentali gravi, Edi Ermes, 2022
